# A taxonomic revision of *Liogenys* occurring in Brazil with an interactive key and remarks on New World Diplotaxini (Coleoptera, Melolonthidae)

**DOI:** 10.3897/zookeys.699.12031

**Published:** 2017-09-19

**Authors:** Mariana Alejandra Cherman, Kleber Makoto Mise, Miguel Angel Morón†, Fernando Z. Vaz-de-Mello, Lúcia Massutti de Almeida

**Affiliations:** 1 Laboratório de Sistemática e Bioecologia de Coleoptera, Departamento de Zoologia, Universidade Federal do Paraná, Caixa Postal 19030, 81581-980, Curitiba, Paraná, Brazil; 2 Universidade Federal de Mato Grosso, Instituto de Biociências, Departamento de Biologia e Zoologia. Av. Fernando Correa da Costa, 2367. Boa Esperança. Cuiabá MT 78060-900, Brazil; 3 Fellow of the Conselho Nacional de Desenvolvimento Científico e Tecnológico (CNPq); 4 Red de Biodiversidad y Sistemática, Instituto de Ecologia, A.C., Xalapa, Veracruz, México

**Keywords:** Description, Diplotaxini, lectotype, morphology, Neotropical fauna, systematics

## Abstract

*Liogenys* Guérin-Méneville, 1831 is the major genus of Neotropical Diplotaxini, with 78 species distributed from Panama to southern Argentina and Chile, except for Ecuador. Due to the large numbers of both described and undescribed species, as well as its agricultural importance, mainly of those in Brazil, *Liogenys* was redefined and redescribed. Nine new species are described: *L. cavifrons* Cherman, **sp. n.**, *L. femella* Cherman, **sp. n.**, *L. piauiensis* Cherman, **sp. n.**, *L. rotundicollis* Cherman, **sp. n.**, *L. pseudosanctaecrucis* Cherman, **sp. n.**, *L. grossii* Cherman, **sp. n.**, *L. pseudospiniventris* Cherman, **sp. n.**, *L. sulcoventris* Cherman, **sp. n.**, and *L. freyi* Cherman, **sp. n.** All the new species are Brazilian, except for the last one, which is Argentinian. Twenty-three Brazilian species are redescribed and illustrated. Five new synonyms are proposed, and 19 lectotypes are designated. New geographical distribution records for 19 species are presented, as well as a key to New World Diplotaxini and Brazilian species of *Liogenys*.

## Introduction

*Liogenys* Guérin-Méneville, 1831 is the largest Neotropical genus within Diplotaxini, encompassing 78 known species, which occur from Panama to southern Chile and Argentina, and approximately 50 more await description (Evans and Smith 2009, Krajčík 2012, Cherman and Almeida 2015). The most recent melolonthine checklists listed 28 species from Brazil (Morón 2004, Evans and Smith 2009).

*Liogenys fusca* Blanchard, *Liogenys suturalis* (Blanchard) and *Liogenys bidenticeps* Moser are the most common species, occurring mainly in the “Cerrado”, a savannah of central Brazil, but they are also present in southern Brazil (Cherman et al. 2011). The two former species are important pests of grain crops due to their rhizophagous habits (Santos et al. 2008, Costa et al. 2009, Santos and Ávila 2009). Less is known about the biology of all other *Liogenys* species, although some adults were found feeding on shrubs causing injury to some fruit trees, and they are attracted by lights at night (Gutiérrez 1951, Silva et al. 1968, Frey 1969). In the Planalto Region of Rio Grande do Sul state, *Liogenys* is the most diverse Melolonthidae genus associated with grain crops (Cherman et al. 2011, 2013, 2014).

According to the latest review (Frey 1969), *Liogenys* is distinguished by its concave clypeus emarginate anteriorly, forming two or four teeth and with lateral margins sinuous; maxillae with four or five teeth and maxillary palpi with a rounded or elongated sensorial fovea on the distal palpomere; mandibles with a bifid tooth apically; labium trapezoidal with a sinuous apical margin, postmentum and mentum fused; pronotum trapezoidal, wider than long; the scutellum with ogival apex; elytra glabrous or setose, with five ribs including the sutural one; metasternum generally setose; protibiae tridentate in almost all of the species; abdomen scaly or bristled laterally; pygidium sub-angled, wider than long, punctured. In males, the metatibia is slender, elongate, with an inconspicuous transverse carina posteriorly, which may be absent; pro- and mesotarsi with the first three tarsomeres flattened, widened and densely setose ventrally; tarsi in females are cylindrical.

*Liogenys* was originally included in Macrodactylini (Dalla Torre 1913, Blackwelder 1944). Then, based on analyses of larvae that suggested similarity with *Diplotaxis* Kirby, Evans (2003) transferred the genus to Diplotaxini together with *Pachrodema* Blanchard. Recently, cladistic analyses of Cherman et al. (2016) based on morphology recovered the monophyly of *Liogenys* within Diplotaxini by including *Hilarianus anguliceps* Blanchard and *Homoliogenys tarsalis* (Moser) as well as placing *Liogenys ferrugata* (Mannerheim) in *Phyllophaga* Harris and *Liogenys micropyga* Blanchard in *Diplotaxis*. In the last revision of *Liogenys* (Frey 1969), many taxonomic errors were found, plus the identification keys (Gutiérrez 1951, Frey 1969) are outdated (Table 1). The taxonomic stability of numerous species must be determined in order to check synonymies, assess availability of type specimens, verify lectotype designations, and make identifications of non-type material deposited in museums during the past two centuries.

**Table 1.** History of *Liogenys* nomenclature.

**1831**: Guérin-Méneville named *Liogenys* for the first time, in a legend of a drawing that says: “*Liogenys* marron, *Liogenys castaneus* Guér”. This drawing is in a plate (N°3), part of the “Voyage de La Coquille”.
**1838**: Guérin-Méneville described *Liogenys* for the first time, based on the species illustrated seven years before. The author also commented the similarity of this species with *Melolontha palpalis* Eschscholtz, 1822, also from Concepción (Chile). He saw differences in the maxillary palp.
**1851**: Blanchard described 12 species collected by A. Saint Hilaire and M. D’Orbigny through South America: *L. concolor*, *L. pallidicornis*, *L. pallens*, *L. fulvescens*, *L. obscurus*, *L. santae-crucis*, *L. denticeps*, *L. quadridentatus*, *L. parvus*, another *L. pallidicornis* (Harold 1869b designated homonymy naming it as *L. xanthocerus*), *L. fuscus* and *L. gayanus* (today synonym of *Pacuvia castanea* Curtis, 1845). Blanchard (1851) also created *Hilarianus* based on *H. anguliceps* (Type-species) and 7 more species: *H. rufinus*, *H. ovalis*, *H. uniformis*, *H. suboblongus*, *H. suturalis*, *H. punctaticollis*, and *H. concolor* (today *L. unicolor* homonymy designated by Evans (2003)). The three latter species were transferred to *Liogenys* by Frey (1969; 1974). There were no additions to that genus later, and the remaining four species were recently transferred to other melolontine genera (Cherman et al. 2016).
**1855**: In “Handbuch der Entomologie”, Burmeister made the following changes: transferred *M. palpalis* in *Liogenys* and redescribed the species; synonymized *L. palpalis* (Eschscholtz) with *L. castaneus* Guérin-Méneville, as he considered that there are no differences in the maxillary palp; transferred *Liogenys* within the “Macrodactilides”, and synonymized *Amphicrania* Dejean, 1833 with *Liogenys*. The author also described 8 species: *L. palmata*, *L. bidentata*, *L. diodon*, *L. micropyga*, *L. morio*, *L. obesa* and more two species today located in *Dilpotaxis*. *Melolontha quadridens* Fabricius, 1798 was transferred to *Liogenys*.
**1856**: Lacordaire redescribed *Liogenys* within the subtribe “Clavipalpides”, confirms synonymy between *L. palpalis* and *L. castaneus*, and synonymized *L. gayanus* Blanchard, 1851 with *Pacuvia castanea* Curtis, 1845.
**1864**: Philippi and Philippi described *L. grandis*.
**1869**: In Coleopterorum Catalogus, Tome IV (Scarabaeidae), Harold synonymized *Pacuvia* with *Liogenys*, adding *L. castaneus* (Curtis) in his *Liogenys* checklist and solved the homonymy of two species created by Blanchard (1851) naming one of them as *L. xanthocerus*. In the checklist, the author put a wrong type-locality for *L. quadridentatus* (Montevideo) and that mistake was repeated in all the subsequent catalogs.
**1873**: LeConte mentioned a similarity of *Liogenys*, *Homalochilus*, and *Hilarianus* with *Diplotaxis*. According to the author, those genera shares propygidium connate with ventrite V.
**1887**: In “Biologia Centrali Americana”, Bates described *L. macropelma*, the only species from Panama. He also suggested the synonymy between *L. quadridentatus* Blanchard and *L. quadridens* (Fabricius).
**1891**: Nonfried described *L. elegans*.
**1892**: Fairmaire described *L. opacicollis* and *L. bidentulus*. Recently, Smith and Ruiz-Manzanos (2010) synonymized *L. bidentulus* with *Pseudoliogenys flavidus* Moser, 1919 and became the type-species of *Pseudoliogenys*.
**1903**: Germain described *L. reichei* based on only one specimen from the Chilean island La Mocha, synonymized with *L. palpalis* by Gutiérrez (1951).
**1913**: In “Coleopterorum Catalogus” Volumen XX, Pars 50 (Melolonthinae IV), Dalla Torre transferred *Liogenys* to Macrodactylini and published an updated checklist. The author suggested that *L. quadridentata* Blanchard, 1851 is a junior synonym of *L. quadridens* Fabricius, 1798.
**1917**: Ohaus transferred *Geniates ferrugatus* Mannerheim, 1829 to *Liogenys*.
**1918**: Reitter created the new genus *Peritryssus* based on *P. excisus*, species found in Sicily.
**1918–1924**: Moser contributed largely in the knowledge of *Liogenys*. He described 31 species and 25 of them are nowadays accepted names: *L. acutidens* Moser, 1919, *L. bidenticeps* Moser, 1919, *L. boliviensis* Moser, 1919, *L. corumbanus* Moser, 1921, *L. densicollis* Moser, 1921, *L. denticulatus* Moser, 1918, *L. excisus* Moser, 1919, *L. flaveola* Moser, 1924, *L. flavidus* Moser, 1918, *L. gebieni* Moser, 1921, *L. kuntzeni* Moser, 1921, *L. laminiceps* Moser, 1919, *L. latipalpus* Moser, 1919, *L. latitarsis* Moser, 1918, *L. mendozanus* Moser, 1918, *L. minutus* Moser, 1924, *L. nigrofuscus* Moser, 1918, *L. pilosipennis* Moser, 1918, *L. tibialis* Moser, 1918, *L. rufocastanteus* Moser, 1918, *L. rufoflavus* Moser, 1918, *L. sinuaticeps* Moser, 1918, *L. spiniventris* Moser, 1918, *L. tarsalis* Moser, 1921, *L. testaceipennis* Moser, 1918.
**1944**: In “Checklist of the coleopterous insects of Mexico, Central America, West Indies and South America”, Blackwelder included all the species created since 1913.
**1951–1952**: Gutiérrez made two great contributions to the genus: in 1951 revised *Liogenys* from Chile and published a key to Chilean species which included four new species: *L. hirtus*, *L. penai*, *L. obesulus*, and *L. wagenknechti*. In the same publication, *Pacuvia* was revalidated after 95 years of being considered a synonym of *Liogenys*. In 1952, Gutiérrez created *Homoliogenys* based on the original description of *Liogenys tarsalis* Moser, 1921. The reasons were discussed in Cherman and Almeida (2016).
**1957**: Martínez described *L. seabrai*, a species collected in Floresta da Tijuca, Rio de Janeiro.
**1964–1975**: During this period, the major contribution to *Liogenys* was provided by Frey, through new species descriptions, revision, redescription of the genus, a key to species, and new synonymies. In total, Frey described 21 species, of which 19 are accepted names: *L. bilobatus* Frey, 1969; *L. calcaratus* Frey, 1970; *L. cartwrighti* Frey, 1969; *L. densatus* Frey, 1969; *L. flavicollis* Frey, 1964; *L. forcipatus* Frey, 1970; *L. forsteri* Frey, 1975; *L. hirtipennis* Frey, 1969; *L. kadleci* Frey, 1970; *L. leechi* Frey, 1967; *L. moseri* Frey, 1969; *L. obesinus* Frey, 1969; *L. opacipennis* Frey, 1969; *L. ophtalmicus* Frey, 1973; *L. parallelus* Frey, 1965; *L. rectangulus* Frey, 1969; *L. rugosicollis* Frey, 1969; *L. vicinus* Frey, 1969; and *L. zischkai* Frey, 1965. In 1969 Frey published a revision and redescription of *Liogenys* including a key to 54 species, with redescriptions and illustrations of male genitalia. He also transferred *Hilarianus suturalis* Blanchard to *Liogenys*. In 1974, Frey transferred *Hilarianus punctaticollis* Blanchard and *H. concolor* Blanchard to *Liogenys*.
**2003**: Evans published *Liogenys unicolor* as a replacement name for Liogenys (Hilarianus) concolor Blanchard, 1851. Keith and Lacroix synonymized *Peritryssus* with *Liogenys*, due to the similar descriptions between *Liogenys palpalis* (Eschscholtz) and *Perytrissus excisus* Reitter (*Peritryssus* species-type). As Baraud (1977, 1992), they mentioned about the probably precedence of *L. excisa* from South America, and occasionally introduced in Sicily.
**2004**: Keith solved the homonymy between *L. excisa* Moser, 1919 (Brazilian species) and *L. excisa* (Reitter, 1918) (Sicilian species), and proposed the name *L. peritryssoidea* for the former.
**2008**: Katovich published a phylogeny of Macrodactylini in which *Liogenys* was transferred to Diplotaxini, together with *Homalochilus*, *Pachrodema*, and *Pacuvia*.
**2009**: The checklist of the New World Melolonthinae by Evans and Smith (2005)which includes *Liogenys*, was updated. Since the last contribution of Frey (1975) there were no descriptions of new species in *Liogenys* until 2015.
**2015**: Cherman and Almeida redescribed *L. pilosipennis* and *L. hirtipennis*, and described *L. moroni*.
**2016**: Cherman et al. recovered a monophyly of *Liogenys* with the following taxonomic changes: *L. ferrugata* and *L. mycropyga* were transferred to other genera; *Liogenys tarsalis* was reallocated in *Liogenys*; and *Hilarianus anguliceps* was synonymized with *L. punctaticollis* Blanchard. As those species were species-types of *Homoliogenys* and *Hilarianus* respectively, both genera became junior synonyms of *Liogenys*.

The present study redefines *Liogenys*, redescribes all Brazilian species, describes new species, and presents an interactive key including, to date, the *Liogenys* described here plus the New World Diplotaxini genera. This type of key allows easier continuous updating of the other known and new *Liogenys* species not included until now.

## Materials and methods

### Material examined

Approximately 2000 specimens of *Liogenys* were studied during this work. The material is deposited in the following institutions, which acronyms follow Evenhuis (2016):

**AMNH** American Museum of Natural History, New York

**CEIOC** Fundação Instituto Oswaldo Cruz, Rio de Janeiro

**CEMT** Setor de Entomologia da Coleção Zoológica, Universidade Federal de Mato Grosso, Cuiabá

**MuBio** Museu da Biodiversidade da Universidade de Grande Dourados, Dourados

**CMNC** Canadian Museum of Nature, Ottawa

**EPGC** Coleção Entomológica Everardo and Paschoal Grossi, Nova Friburgo

**DZUP** Coleção Entomológica Pe. J.S. Moure, Curitiba

**MCNZ** Museu de Ciências Naturais da Fundação Zoobotânica do Rio Grande do Sul, Proto Alegre

**IBSP** Instituto Biológico de São Paulo, São Paulo

**IADIZA** Instituto Argentino de Investigaciones de Zonas Áridas, Mendoza

**INPA** Instituto Nacional de Pesquisas da Amazônia, Manaus

**MACN** Museo Argentino de Ciencias Naturales Bernardino Rivadavia, Buenos Aires

**MLPA** Museo de La Plata, La Plata

**MLUH** Martin-Luther-Universität, Wissenschaftsbereich Zoologie, Halle (Saale)

**MNHN** Muséum National d’Histoire Naturelle, Paris

**MNRJ** Museu Nacional do Rio de Janeiro, Rio de Janeiro

**MZSP** Museu de Zoologia da Universidade de São Paulo, São Paulo

**NHMB** Naturhistorisches Museum, Basel

**NHRS** Naturhistoriska riksmuseet, Stockholm

**SDEI** Senckenberg Deutsches Entomologisches Institut, Müncheberg

**ZMHB** Museum für Naturkunde der Leibniz Gemeinschaft Institut, Berlin

**ZMUC** University of Copenhagen Zoological Museum, Copenhagen

### Morphological study

**Figures 1–3.**
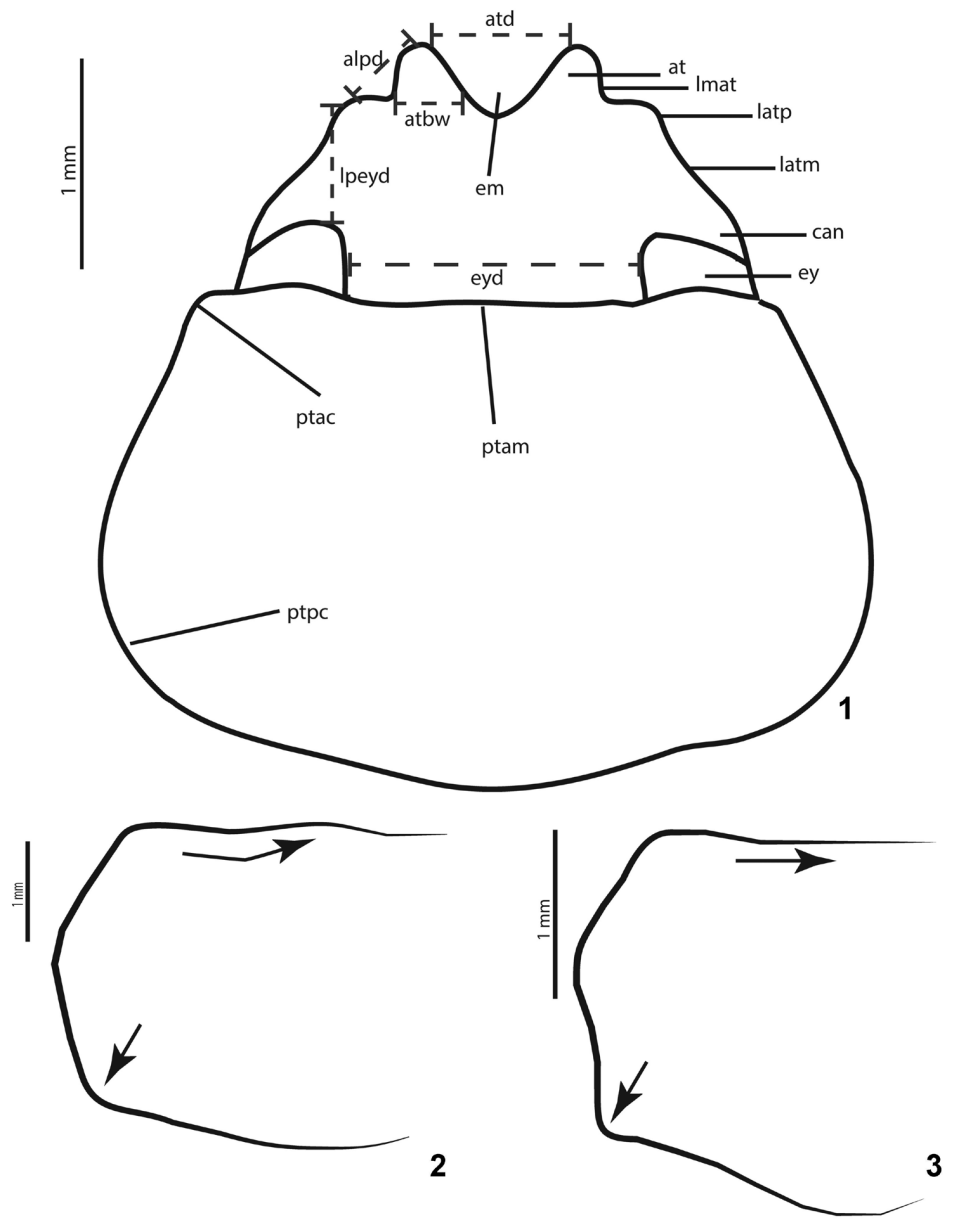
Main characters of *Liogenys* Guerin-Méneville, 1831. **1** Head and pronotum parts, pronotal posterior corners obsolete **2** Pronotal anterior margin barely produced and posterior corners rounded **3** Pronotal anterior margin straight and posterior corners angled. Abbreviations: alpd= anterior-to-lateral projection distance; at= anterior tooth; atbw= anterior tooth basal width; atd= anterior teeth distance; can= canthus; em= emargination; ey= eye; eyd= eye-to-eye distance; latm= lateral margin of clypeus; latp= lateral projection; lmat= lateral margin of anterior tooth; lpeyd= lateral projection-to-eye distance; ptac= prothorax anterior corner; ptam= prothorax anterior margin; ptpc= prothorax posterior corner.

Mouthparts and male genitalia, when available, were dissected and examined for all species. For describing species and identification keys, the taxonomic characters and terms used are those proposed by Moser (1918), Frey (1969), Cherman (2015), and Cherman et al. (2016). The standards used for characters followed Cherman and Almeida (2015), plus other features:

*Shape of outer sides of anterior clypeal teeth*: When the anterior teeth of the clypeus follow the lateral margin of the clypeus, it means that the outer sides of the anterior teeth and the lateral clypeal margin are continuous. The outer sides of anterior teeth being parallel or sub-parallel means that there is a clear difference in the direction of this margin compared with the lateral margin of the clypeus. In this case, a more or less pronounced angle appears between them (Fig. 1).

*Clypeus, emargination size*: This is the same feature as “Distance between clypeal teeth” in Cherman and Almeida (2015). In this case, “narrow” corresponds to “teeth close together”, while “wide” corresponds to “teeth separated” (Fig. 1).

*Clypeal lateral margin, shape*: As in Cherman and Almeida (2015). The lateral margin may be straight, concave, or convex. Occasionally, the convex lateral margin may show a projection more or less pronounced (Fig. 1).

*Metasternum, anterior margin, position*: Sometimes hardly visible, hidden beneath the mesofemur.

*Mesotibia, position and extension of carinae*: The apical carinae and, when present, also the basal one, are located on the posterior or postero-external surface. If the apical carina is extended across the entire width of the tibia, it is considered complete.

*Propygidium, setae*: The propygidium in *Liogenys* is partially visible, but in a few cases, it may not be visible. When it is visible, the surface may be described as glabrous or setose (scaly or bristled).

*Basal region of parameres*: The same structure as in Cherman and Almeida (2015), although in that work, there was a mistake in the abbreviation and the correct form is BR instead of RB.

*Parameral split, position*: The split point is where the parameres begin to be separated and corresponds to the end of the basal region. This point might be at different levels depending on the species. Taking into account the portion extended from the base up to the transverse midline of the parameres (Cherman et al. 2016), the split point might be placed at the third portion (near the midline), at 2/3 or at 1/3 (near the base of the parameres) (Figs 48, 49, and 50, respectively).

*Parameres, shape in lateral view*: The parameres show different shapes beyond the split point. They may be convex, concave, or straight. When straight, the parameres and the basal region may be coplanar or not coplanar, that is, not on the same plane (Figs 54, 55).

In the species’ descriptions and redescriptions, each species name is followed by author(s), publication year and figure(s) number. In cataloging lists, the following abbreviations are used:

**biol**. biological data;

**cat**. catalogue;

**check**. check list;

**orig. desc**. original description;

**red**. redescription;

**rev**. revision;

**Stat. Res**. revalidated name;

**syn**. synonymy;

**sys**. systematics.

Labels of the type material are arranged in sequence from top to bottom, where the data for each label are within double quotes (“ ”), a slash (/) separates the rows and “,” separates labels. Information between brackets ([]) provides additional details written on the labels. Biological notes are based on specimen labels or extracted from the literature.

The geographical distribution is based on previously published records and specimens examined. The abbreviations used in this work for states/departments from each country are as follows:

ARGENTINA: **CA**=Catamarca, **CH**=Chaco, **CO**=Córdoba, **CR**=Corrientes, **FO**=Formosa, **JU**=Jujuy, **MI** = Misiones, **SA**= Salta, **SE** = Santiago del Estero, **SF**=Santa Fé, **SL**= San Luis, **TU**= Tucumán.

BOLIVIA: **CB**=Cochabamba, **LP**= La Paz, **SC**= Santa Cruz, **TA**=Tarija.

BRAZIL: **AL**= Alagoas, **BA**=Bahia, **CE**=Ceará, **DF**=Distrito Federal, **ES**= Espírito Santo, **GO**=Goiás, **MA**= Maranhão, **MG** = Minas Gerais, **MT**=Mato Grosso, **MS**=Mato Grosso do Sul, **PA** = Pará, **PB**= Paraíba, **PE**= Pernambuco, **PI**=Piauí, **PR** = Paraná, **RJ**=Rio de Janeiro, **RN**=Rio Grande do Norte, **RS**=Rio Grande do Sul, **SC**=Santa Catarina, **SP**=São Paulo, **SE**=Sergipe.

PARAGUAY: **AS**= Asunción, **BQ**= Boquerón, **CN**= Concepción, **GU**= Guairá, **IT**= Itapúa, **PA** = Paraguarí, **PH**=Presidente Hayes, and **SP**= San Pedro.

New geographical distribution records are in bold type.

Species descriptions and redescriptions are presented following Ratcliffe's, (2013) recommendations. The author of all the new species contained in this manuscript is the first author of this work, Mariana Cherman, who was responsible for coining the name and for satisfying all other availability criteria.

Shapefiles from the Biogeographical regionalization of the Neotropical region (Morrone 2014) by Löwenberg-Neto (2014) were used to perform the distributional maps. As the geographical records known for *L. rufocastanea* Moser are only the ones of the type series (Paraguay, Brazil), not referring to locality/localities in those countries, we decided to not include this species in a map. In the case of *L. hirtipennis* Frey, *L. pilosipennis* Moser and *L. moroni* Cherman, which are included in the key, distributional maps are in Cherman and Almeida (2015).

An online interactive key was produced using the software LUCID 3.3, and the characters codified in the Lucid matrix were based on adult morphology. Interactive keys have some advantages in comparison with dichotomous keys, most notably being easier to update, as they are available online (Penev et al. 2009). Lucid Player has a layout with four windows. The upper-left window shows the characters that may be selected by the user during the identification process. The bottom-left window shows selected characters/states. The upper-right window shows the remaining taxa, while the bottom-right shows the discarded taxa. Therefore, when the user selects character states, the taxa that do not possess it are discarded until the identification is reached.

The database built in this work is available in the supplementary material of this article to enable anyone to use those data for further studies. For more information concerning Lucid keys, visit <http://www.lucidcentral.org>.

## Results

An interactive key to New World Diplotaxini is provided at: <http://keys.lucidcentral.org/keys/v3/diplotaxini/> including the following genera: *Diplotaxis*, *Pachrodema*, *Homalochilus* Blanchard, *Pacuvia* Curtis and *Liogenys*. For the latter genus, it includes, until now, all Brazilian species and the new species described in this work. This key will be updated continuously with known and new species of the entire genus.

### Taxonomy

#### Liogenys

Taxon classificationAnimaliaColeopteraMelolonthidae

Guérin-Méneville, 1831

Liogenys Guérin-Méneville, 1831: pl. 3 (orig. desc. [drawing of L. castaneus]); Guérin-Méneville 1838: 84 (red.); Blanchard 1851: 167 (rev.); Burmeister 1855: 13 (red. and rev.); Lacordaire 1856: 269 (red.); Harold 1869b: 1140 (check.); Dalla Torre 1913: 318 (check.); Blackwelder 1944: 228 (check.); Frey 1969: 38 (key); Evans 2003: 206 (check.); Evans and Smith 2005: 174 (check.); Evans and Smith 2009: 175 (check.); Cherman et al. 2016: 23 (sys.). Type-species. Liogenys castaneus Guérin-Méneville, 1831(monotypy).
Amphicrania Dejean, 1833: 163 (cat.); Burmeister 1855: 13 (syn.). Type-species: Amphicrania palpalis (Eschscholtz, 1822) (monotypy).
Hilarianus Blanchard, 1851: 168 (orig. desc.); Cherman et al. 2016: 23 (syn.) Type-species: Hilarianus anguliceps Blanchard, 1851.
Peritryssus Reitter, 1918: 77 (orig. desc.); Keith and Lacroix 2003: 48 (syn.) Type-species: Peritryssus excisus Reitter, 1918 (monotypy).
Homoliogenys Gutiérrez, 1952: 216 (orig. desc.); Cherman et al. 2016: 23 (syn.) Type-species: Liogenys tarsalis Moser, 1921a (monotypy).

##### Diagnosis.

*Liogenys* is distinguished from all other Diplotaxini genera by the following combination of features: frons and clypeus concave in dorsal and lateral view; basal protarsomere (I) shorter than tarsomere II; mesotibial transverse carina/ae provided with spines shorter or equal to those forming the apical crown; metacoxae with bristles or scales, never both at the same time, and pygidium with umbilicate punctures.

##### Redescription.

**Figures 4–16.**
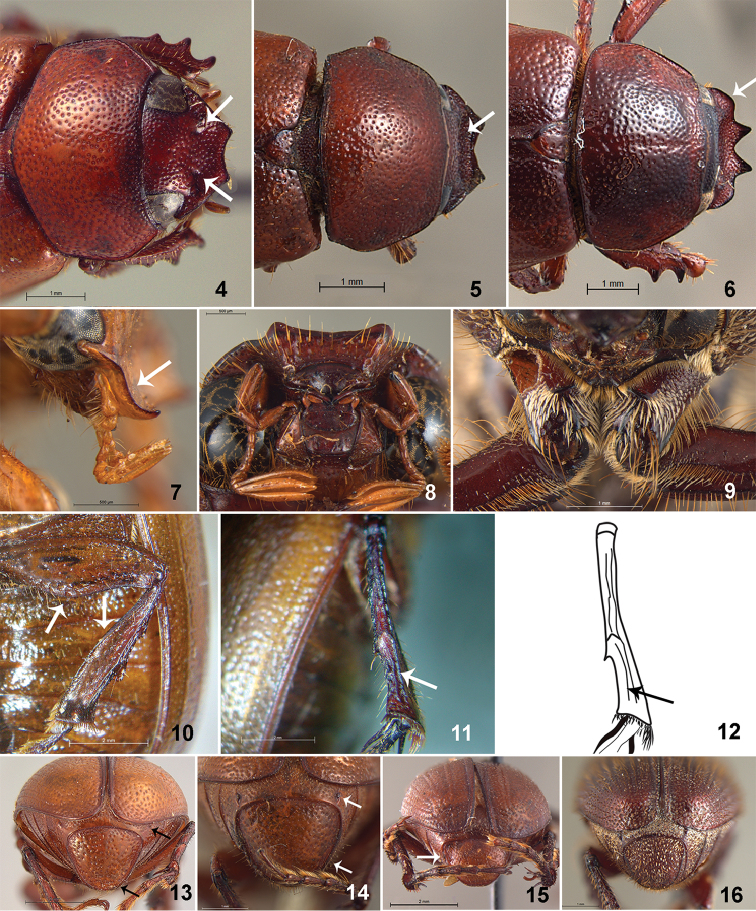
**4** *Liogenys bidenticeps* Moser fronto-clypeal impressions **5** *L. santaecrucis* Blanchard frons and clypeus concave **6** *L. forcipata* Frey clypeal lateral projection **7** *L. flavida* Moser clypeus S-shaped **8** *L. testaceipennis* Moser clypeus and labrum, ventral view **9** *L. fusca* Blanchard procoxae **10** *L. tibialis* Moser metafemur and tibia **11–12** *L. santaecrucis* Blanchard metatibia, posterior view **13–16** Pygidium: **13** *L. spiniventris* Moser **14** *L. quadridens* (Fabricius) **15** *L. cartwrighti* Frey **16** *L. densicollis* Moser.

**Figures 17–22.**
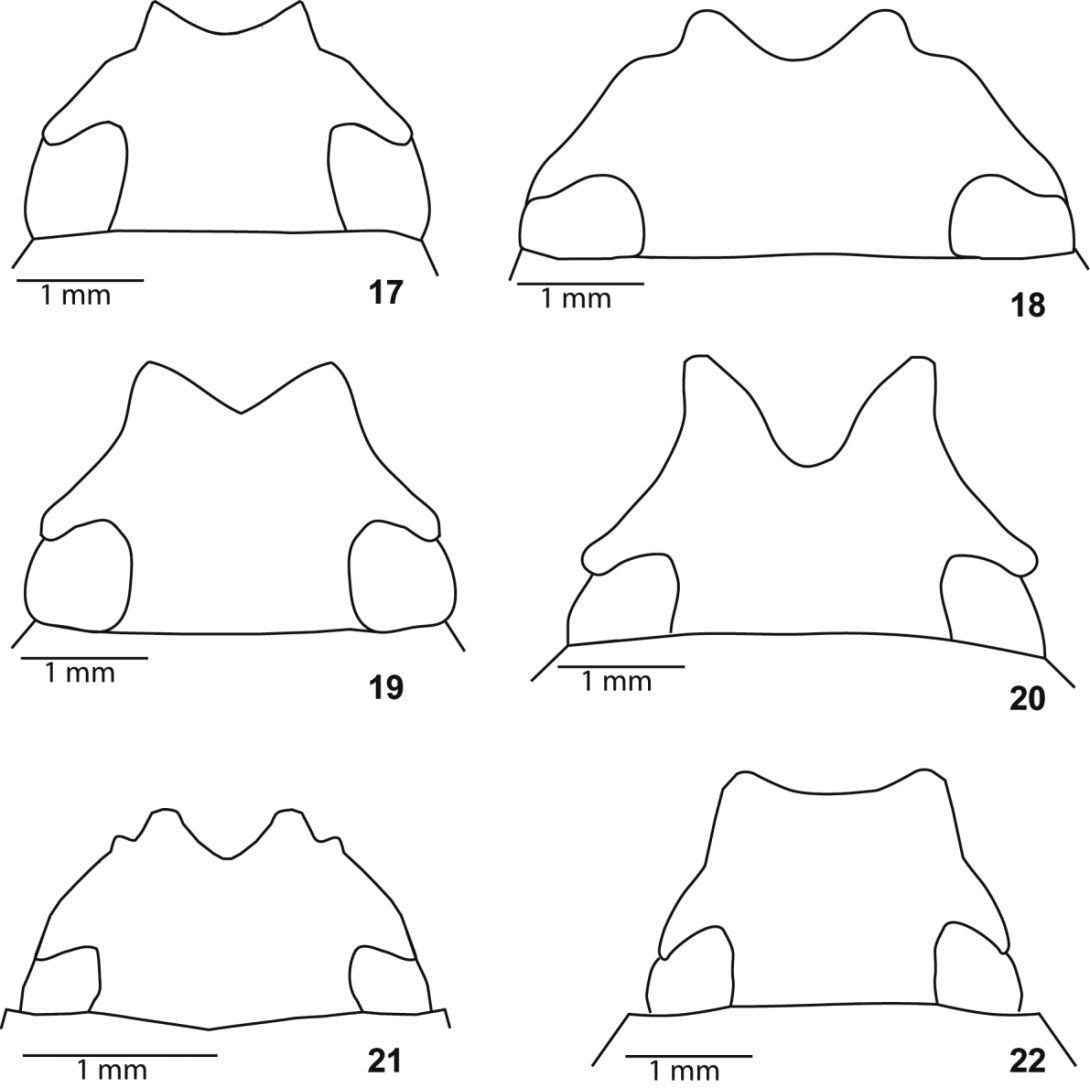
Clypeus. **17** *Liogenys acutidens* Moser **18** *L. fusca* Blanchard **19** *L. santaecrucis* Blanchard **20** *L. bilobata* Frey **21** *L. corumbana* Moser **22** *L. rectangula* Frey.

**Figures 23–33.**
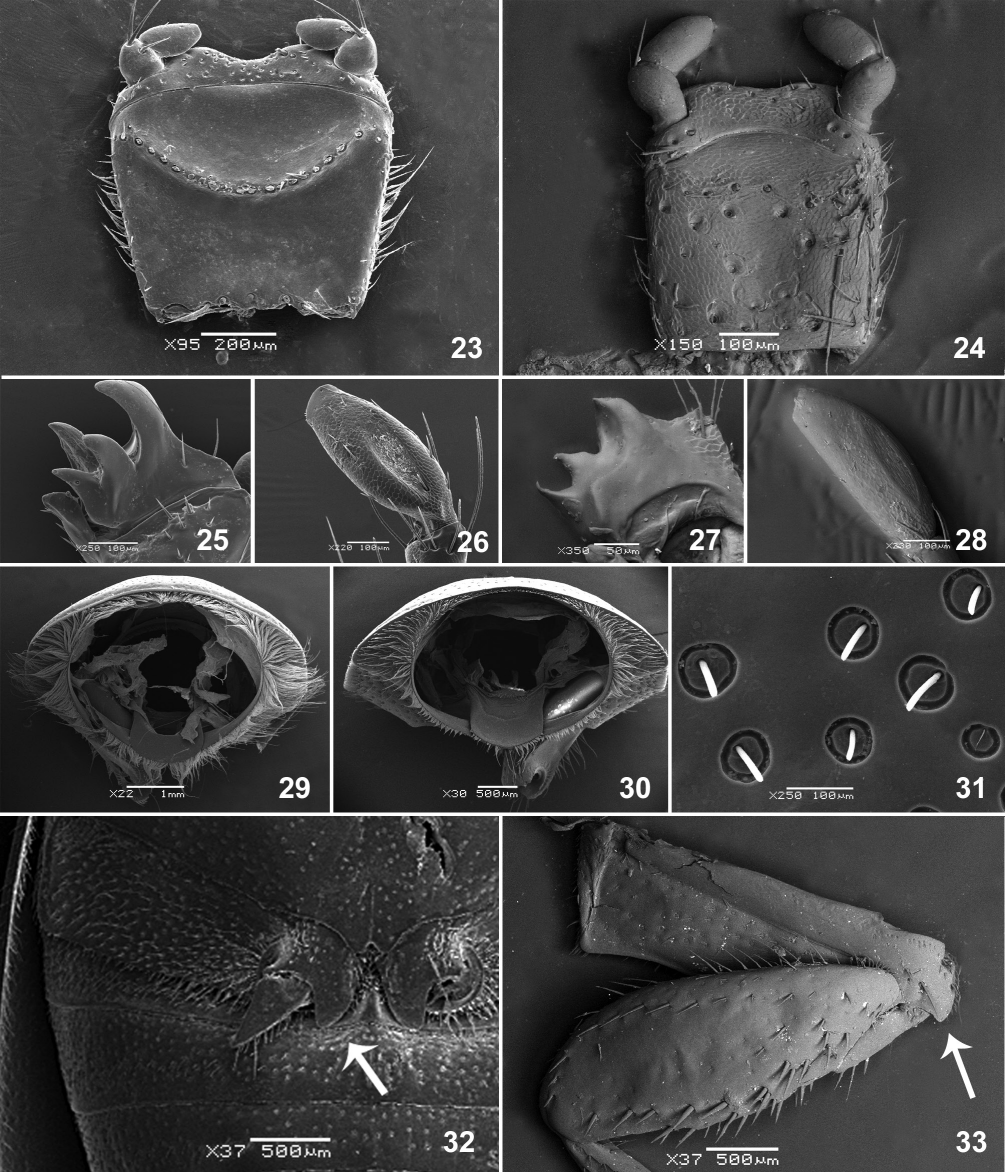
**23–24** Labium **23** *Liogenys palpalis* (Eschscholtz) **24** *L sinuaticeps* Moser **25–28** Maxilla teeth and distal palpomere **25–26** *L. bidenticeps* Moser **27–28** *L. sinuaticeps* Moser **29–30** Pronotum basal margin, posterior view **29** *L. palpalis* **30** *L. bidenticeps* **31** *L. bidenticeps* pygidial punctures **32–33** basal lobe of metacoxae **32** *L. bidenticeps* **33** *L. sinuaticeps*.

**Figures 34–47.**
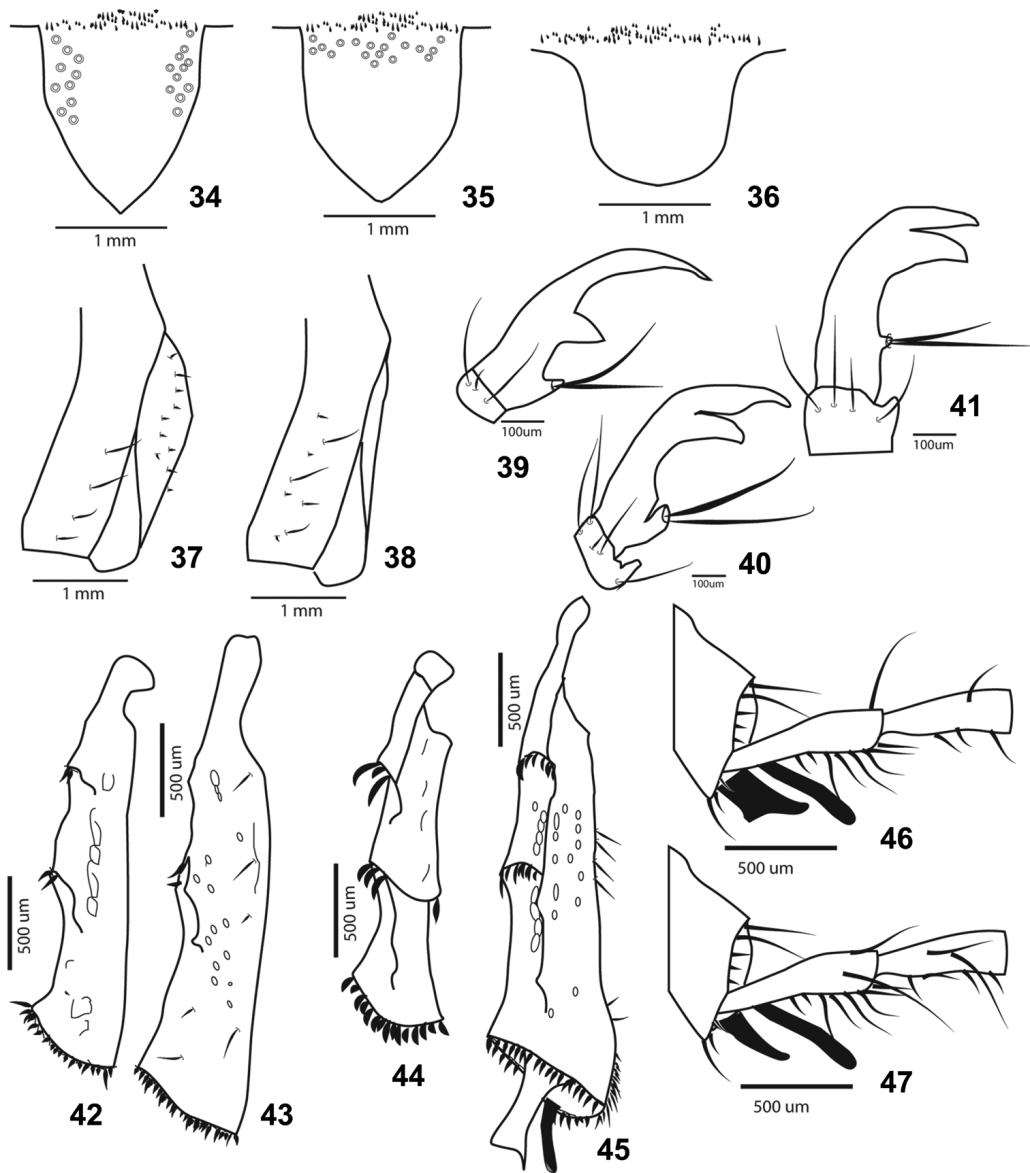
**34–36** Scutellum **37–38** Pygidium; lateral view **39–41** Claws **42–45** Meso-and metatibia: **42–43** Cylindrical **44–45** Quadrate in cross section **46–47** Metatibial spurs.

Length 6.5–16.3 mm; width: 3.5–8.3 mm. Body sub-parallel, sometimes wider in the posterior half; elytra and body may be unicolored or with different colors, which vary from yellowish, brownish, reddish-brown, purplish-red to black. *Head*: distance between eyes commonly twice the width of one eye, but occasionally three to five times; frons and clypeus forming together a concavity (Fig. 5); fronto-clypeal suture absent or barely distinguishable (as in *L. tarsalis* and *L. forcipata* Frey); fronto-clypeal impressions evident in frontal view (Fig. 4); clypeus anteriorly bent forward and emarginate (Fig. 1), clypeal emargination may be angled, sub-angled or rounded, wide or narrow, mostly forming two tooth-like projections (Figs 17–22); the lateral margin of the clypeus may form a projection, rounded or sharp, in this case it resembles a tooth (Figs 6, 18, 21); clypeus S-shaped in lateral view (Fig. 7); outer margin of the maxillae straight (see Cherman et al. 2016), with four or five teeth at the apex (Figs 25, 27); distal palpomere up to two-fold wider than its apex, sensorial surface generally forming a fovea (except in *L. sinuaticeps* Moser and *L. unicolor* Evans) deep or shallow and with variable length (Figs 26, 28); labium trapezoidal with maximum width at the apex (except in *L. sinuaticeps*); ligula emarginate anteriorly; mentum excavated on the disc (except in *L sinuaticeps*, Fig. 24); ligula length shorter than the excavation (Fig. 23); labrum in frontal view convex on upper margin and shorter than the ventral portion of clypeus (Fig. 8); antenna 9- or 10-articulated. *Prothorax*: pronotum wider medially, forming a lateral convexity more or less pronounced (Figs 1–3); anterior margin of pronotum may be straight or slightly produced medially (Figs 2, 3); sometimes flanged anteriorly (as in *L. corumbana* Moser and *L. fusca*; Fig. 61D, 64D); pronotal posterior corners right or obtuse-angled, rounded or obsolete (corners not distinguishable) (Figs 1–3); prothorax setose posteriorly, covered with short or long bristles (Figs 29–30); proepisternum setose, scaly or bristled, with short and/or long bristles. *Pterothorax*: Scutellum triangular, ogival, or rounded; normally with punctures grouped at the base and/or at the sides, or sometimes without any pattern (Figs 34–36); metasternum more or less setose laterally; distance between meso- and metacoxae up to twice the length of the metacoxa (except in *L. concolor*). *Elytra*: glabrous or setose; densely and coarsely punctured; elytral suture with non-uniform width; elevated or not, unicolored with the elytron or darker; three or four elytral ridges more or less noticeable, separated from each other by gaps that are twice as wide as one ridge, the first gap equal to or narrower than the second one; subapical callus of elytron closer to the elytral suture than to the external margin (feature of all Neotropical Diplotaxini; see Cherman et al. 2016). *Legs*: procoxae setose, bristled and/or scaly (Fig. 9), protibial inner margin concave and the outer one with three teeth perpendicular to the tibial axis (see Cherman et al. 2016), the basal tooth is always the smallest, in males the basal tooth is more reduced and may be absent depending on the species; the two other protibial teeth may be equal in size or not, when different, the apical one is commonly the longest, but in some cases the middle is the longest; protibial disc with two medial longitudinal carinae; protarsi longer than the head; protibial apical spur on inner margin commonly present; protarsal tarsomere I shorter than tarsomere II; basal apophysis of metacoxae produced beyond the outer margin of trochanter (Fig. 32) (except for *L. tarsalis*, *L. sinuaticeps* and *L. unicolor*, Fig. 33); mesotibiae with one or two transverse carinae bearing spines, mesotibial spines equally as long as the spines of the apical crown (Figs 42–45), the apical carina may be complete or incomplete (as described above, in “standards” of morphological study); pair of apical spurs on apex of meso- and metatibia equal to or different in size, in male metatibia the shorter spur varies in shape among the species (Figs 46–47), the gap between spurs is narrower or equal to the base of one spur; tarsi covered ventrally with setose pads; length of basal metatarsomere varies from equal up to one-half the length of the second; all claws bifid and generally symmetrical, they vary in the size of the upper tooth and in the distance between teeth (Figs 39–41). *Abdomen*: propygidium visible, slightly visible or hidden by the elytra; glabrous or setose, with scales and/or bristles; pygidium convex or flat (Figs 37–38), disc glabrous or setose, throughout the surface or only at apex; punctures, if present, umbilicate (Fig. 31); shape sub-quadrate or sub-trapezoidal (Figs 13–16), twice the length of ventrite V or more; depending on the species, the maximum width either exceeds or does not exceed the width between the propygidial spiracles (Figs 13–14).

##### Dimorphism.

**Figures 48–51.**
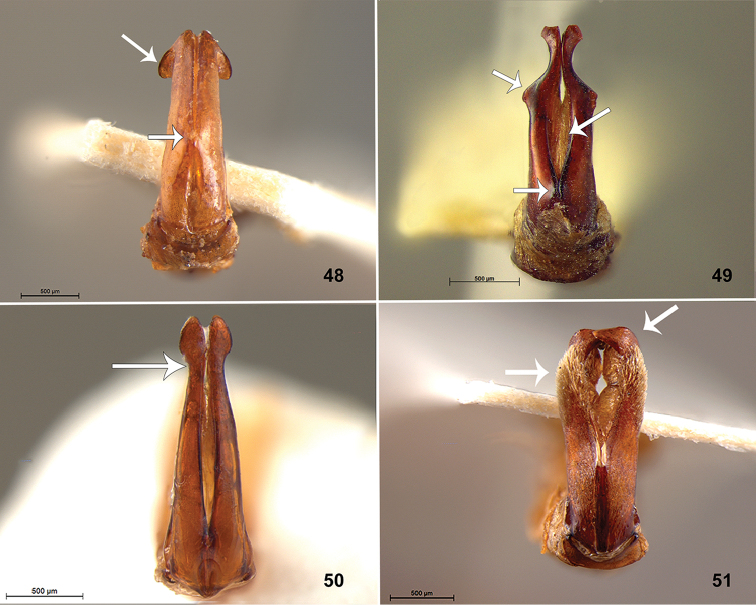
Parameres, dorsal view. **48** *Liogenys pallens* Blanchard **49** *L. acutidens* Moser **50** *L. freyi* Cherman, sp. n. **51** *L. forcipata* Frey.

**Figures 52–55.**
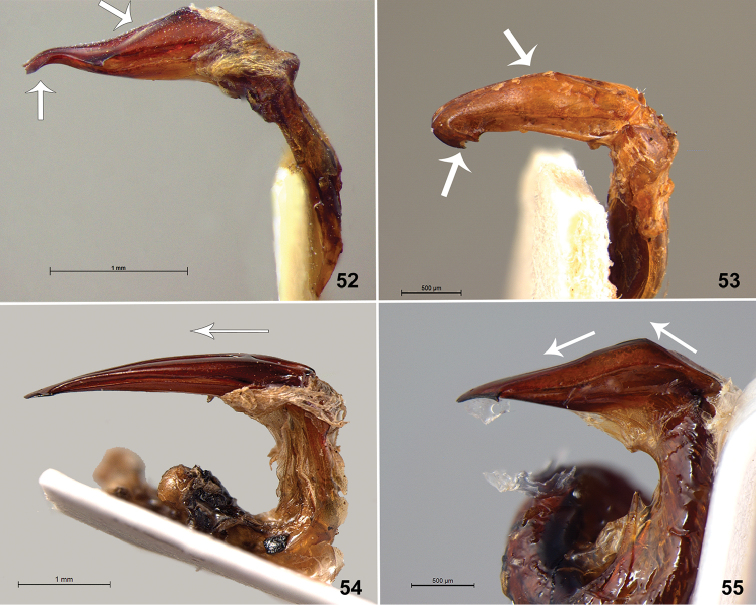
Parameres, lateral view. **52** *L. acutidens* Moser **53** *L. pallens* Blanchard **54** *L. bidentata* Burmeister **55** *L. suturalis* (Blanchard).

***Female***. Length: 6.7–16.3 mm; width: 3.7–8.3 mm. *Size and body-shape*: wider than males, mainly at the posterior third. *Head*: clypeus wider; distance between eyes slightly wider; punctures in head and pronotum with different distribution and deepness; lamellae equal to or shorter than flagellum. *Legs*: protibiae shorter and wider, teeth of outer margin wider; mesotibiae wider at the apex, mesotibial apical transverse carina at the postero-external surface commonly complete and prominent; metatibiae wider, mainly on the apex; not carinated on inner margin; tarsi cylindrical and equally wide in all legs. *Abdomen*: the pygidium may differ from the male in the shape and roundness, and this difference varies among the species. ***Male***. Length: 6.5–14.7 mm; width: 3.5–7.2 mm. *Head*: Lamellae generally longer or equal to flagellum, in a few cases shorter; clypeal lateral projection, when present, may be more pronounced than in the female of certain species. *Legs*: protibiae with two or three teeth; posterior margin of metafemur generally straight but may be produced medially in some species (Fig. 10), metatibiae widened towards apex; carinated along the inner margin entirely or excepting the apex; the inner margin commonly straight towards apex, in some cases is produced abruptly from the sub-basal or medial portion (Fig. 10); metatibial inner margin not carinated in a few cases; apex setose throughout the inner surface but in a few cases glabrous; on posterior surface, one or two transverse carinae and discontinuous longitudinal carina may be present or absent (Figs 11–12), pair of apical spurs equal to or different in size from each other, the shorter one may be fusiform or truncated (Figs 46–47); pro- and mesotarsi enlarged (in *L. tarsalis* also the metatarsi), protarsi commonly wider than mesotarsi and two-fold wider than metatarsi (except for *L. unicolor* and *L. macropelma* Bates); tarsi shiny or opaque. *Abdomen*: ventrites III, IV and/or V ornate in some species, with different-sized protuberances or sulcated (as in *L. testaceipennis* Moser, *L. spiniventris* Moser, *L. pseudospiniventris* Cherman, sp. n., *L. grossii* Cherman, sp. n. and *L. sulcoventris* Cherman, sp. n.); pygidium more or less angled than that of the female. *Parameres*: basal region (BR) grooved at its longitudinal midline; the groove width varies depending on the species; parameral split at three different levels: apical third, medially (2/3) or basally (1/3) (Figs 48, 49, 50); outer margin of parameres sometimes furnished with medial (Fig. 49) and/or apical projections (Fig. 48); may be apically narrowed (Fig. 51) or not; inner margins of parameres straight, divergent, or convergent; apex of parameres indistinct or with several distinctive shapes: harpoon-like; spatula-like, fusiform, etc.; in harpoon-like parameres, apical length varies in relation to the total length of the parameres; in lateral view parameres convex, concave, or straight from the parameral split (Figs 52–55); when straight, the parameres and the basal region may be coplanar or not coplanar.

##### Etymology.

The name *Liogenys* is composed of the Greek words λείος (*leios*: smooth) and γένυς (*genys*: mentum) referring to smooth or glabrous mentum (Guérin-Méneville 1838; Harold 1869a). The International Code of Zoological Nomenclature (ICZN 1999), Article 31.2, says that “A species-group name, if it is or ends in a Latin or Latinized adjective […], must agree in gender with the generic name with which it is at any time combined”. Also, Article 30.1.2 says that: “A genus-group name that is or ends in a Greek word transliterated into Latin without other changes takes the gender given for that word in standard Greek dictionaries”. *Liogenys* is feminine based on the Greek “génys, géneion” (Smith and Ruiz-Manzanos 2010). Historically, *Liogenys* was incorrectly treated as masculine by some authors (Guérin-Méneville 1831, Blanchard 1851, Harold 1869b, Moser 1918, Moser 1919, Moser 1921a, b, Moser 1924, Gutiérrez 1951, Frey 1965, Frey 1969, Frey 1970, Frey 1973). However, Burmeister (1855), Blackwelder (1944), Evans (2003) and Keith (2004) correctly considered the name to be feminine. According to this, all species names must be made feminine, and in this work we have changed them as required.

##### Distribution and habitats.

*Liogenys* is a South American genus with 69 species. Only one species has been collected in Sicily: *Liogenys excisa* (Reitter, 1918). However, according to Baraud (1977, 1992) this species must have been occasionally introduced from South America, as it has never been seen after that. Keith and Lacroix (2003) compared *Peritryssus excisus* Reitter with *L. palpalis* (Eschscholtz, 1822) and synonymized these genera. They mentioned that the description of *L. obesula* Gutiérrez matches that of *L. excisa*, but they did not synonymize these species as they had not examined the type of *L. obesula*.

*Liogenys* occurs from Panama, northern Colombia and northwestern Venezuela (10°N) through southern South America, including Chile (39°S) and Argentina (46°S). The species occur in almost all biogeographical environments and at altitudes up to 4,100 m in Bolivia (16°S) and 4,000 m in Chiriqui, Panama (9°N). The species richness is concentrated mainly in Brazil, with 34 species already recorded plus 13 for which descriptions are being prepared, and in Argentina, with 33 species in addition to 23 awaiting description. Bolivia holds ten species (plus three still undescribed); Paraguay, eleven species; Chile, seven species (plus one undescribed); Colombia, Panama and Venezuela, three species: *L. gebieni* Moser; *L. quadridens* Fabricius and *L. macropelma*) (plus three new species for which descriptions are being prepared); Peru, *L. leechi* Frey; and Uruguay *L. pallens* Blanchard. There are no records of *Liogenys* from Ecuador (Fig. 88).

##### Discussion.

The Neotropical Diplotaxini included in the interactive key form a well-established clade and the features shared among them are discussed in Cherman et al. (2016). Some of those features have exceptions, as follows: elytral suture generally narrowed at post-scutellar level (except in *Homalochilus punctatostriatus* Blanchard) and protibiae with an apical spur (except in *Liogenys tarsalis*). Furthermore, a common feature in Neotropical Diplotaxini, the enlarged tarsi in males, covered ventrally by pads of abundant bristles, is also seen in non Neotropical Diplotaxini as in *Apogonia* Kirby, *Ceratogonia* Kolbe, *Dichecephala* Brenske and in a few *Diplotaxis*.

##### *Liogenys* and related genera.

The genera most closely related to *Liogenys* are *Pacuvia*, *Pachrodema*, and *Homalochilus* (Cherman et al. 2016). Like species of *Pacuvia*, *Liogenys* has the anterior margin of the clypeus emarginate; metasternum with sparse scales laterally (as in *L. obscura* Blanchard, *L. densicollis* Moser; *L. pallens* and *L. forcipata*) and the outer margin of metacoxae parallel to the elytral margin. *Liogenys* is distinguished from *Pacuvia* in that the latter shows a coplanar shape of the frons and clypeus in dorsal view; the maxillae with less than four teeth (except in *P. philippiana* Gutiérrez); the labium longer than wide (in common with *L. sinuaticeps*), distal labial palpomere more globose than the preceding, the ligula straight anteriorly, surface of mentum smooth (without any excavation); pronotal length equal to the length of tarsomeres I, II and III together (in common with *L. unicolor* and *L. macropelma*); pronotum narrower than the base of elytra; prothorax bristled and/or scaly abundantly posteriorly; innermost gap between elytral ridges wider than the adjacent; distance between meso- and metacoxae more than two-fold the metacoxa length (in common with *L. kuntzeni* Moser); protibial apical tooth parallel to longitudinal axis while basal and/or medial tooth oblique to longitudinal axis, only one longitudinal carina on protibia; basal apophysis of metacoxae not produced posteriorly (beyond the margin of trochanter); basal metatarsomere less than one-half the size of tarsomere II; propygidium completely hidden by the elytra; pygidium twice as wider as it is long. In males, metatibiae narrowed subapically and inner margin not produced (in common with *L. sinuaticeps*, *L. tarsalis* and *L. forcipata*). With *Pachrodema*, *Liogenys* shares the trapezoidal shape of the labium and its distal palpomere not globose; ligula emarginate anteriorly, mentum surface excavated, ligula shorter than the mentum excavation; pronotum longer than the length of tarsomeres I, II and III together; pronotum anteriorly slightly narrowed forming a depressed ring (in common with *L. kuntzeni*, *L. flavida* Moser, *L. calcarata* Frey, *L. kadleci* Frey; *L. palpalis*, *L. hirta* Gutiérrez and *L. wagenknechti* Gutiérrez); the inner gap between elytral ridges narrower or equal in width as the adjacent; inner margin of protibiae concave, protibial basal and middle teeth perpendicular to the longitudinal axis, two longitudinal carinae. *Liogenys* is distinguished from *Pachrodema* in that the latter shows clypeus rounded or sub-emarginate, never forming anterior tooth-like projections; sensorial area of the maxillary distal palpomere grooved and in a dorso-lateral position (except *P. lucida* Blanchard); constriction between pro- and pterothorax prominent; distance between meso- and metacoxae equal to metacoxae length (in common with *L. concolor* (Blanchard)); apical tooth of protibiae oblique to the longitudinal axis; inner margin of mesotibiae concave; lateral margin of metacoxae oblique towards pygidium; metatibial inner margin bent and produced apically; apex surface convex at the insertion point of the tarsomere, gap between apical spurs wider than the base of a single spur. *Homalochilus* shares the same features with *Liogenys* as well as *Pachrodema*. Additionally, the lateral margin of metacoxae is parallel to the elytral margin. Males of *H. niger* Blanchard show metatibiae straight and produced apically. *Liogenys* is distinguished from *Homalochilus* in that the latter shows a shorter body, up to three times longer than the pronotum (in common with *L. concolor*); labrum longer than ventral face of clypeus; pronotum forming a visibly depressed ring anteriorly, pronotal anterior angles slightly (*H. niger*) or noticeably (*H. punctatostriatus*) produced; punctures of pronotum not umbilicate, pronotum wider than the base of elytra; distance between meso- and metacoxae equal to metacoxal length (in common with *L. concolor*); pygidium glabrous (in common with *L. sinuaticeps* and *L. unicolor*), but punctures not umbilicate. In males, basal region of parameres longer than the parameres length beyond the split point. The frons of *H. punctatostriatus* is longer than the clypeus, clypeus sublobed anteriorly (not emarginate) and ventrite V is longer than ventrite IV (an exception among the Diplotaxini, with all ventrites equally long).

##### Biological notes.

*Liogenys* species, as well as most of the Melolonthinae, are root-feeding as larvae, while adults feed on leaves and exhibit nocturnal activity (Gutiérrez 1951, Britton 1957, Morón et al. 1997, Evans 2002, Morón 2004). Biezanko et al. (1949) mentioned adults of *L. densicollis* damaging corn in Rio Grande do Sul (Brazil). However, we suggest that this record should be considered doubtful because among the huge number of *L. densicollis* specimens studied (60 specimens from 10 museums: AMNH, CEMT, CMNC, IADIZA, MLPA, MZSP, NHMB, SDEI, VMDZ and ZMHB), the geographical records found were only from Argentina and Bolivia. Silva et al. (1968) mentioned *L. tibialis* in Rio Grande do Sul feeding on leaves of peach-tree. Chilean *Liogenys* were recorded together with *Pacuvia* species feeding on the following shrubs: Peumo, *Cryptocarya alba* (Molina) Looser (Lauraceae), Litre, *Lithraea caustica* (Molina) Hook. et Arn. (Anacardiaceae) and Quillay, *Quillaja saponaria* Molina (Rosaceae) (Gutiérrez 1951).

Of the 34 species of *Liogenys* now known in Brazil, only five are associated with crops. In Rio Grande do Sul, Cherman et al. (2011) recorded four species: *Liogenys concolor* Blanchard (= *L. obesa* Burmeister, syn. n.) and *L. fusca* Blanchard in oat crops; *L. bidenticeps* Moser and *L. sinuaticeps* in ryegrass crops. According to Cherman et al. (2014), the abundance of *L. sinuaticeps* is higher in non-cultivated than in cultivated fields, unlike *L. bidenticeps* and *L. concolor*. In Mato Grosso do Sul state, adults of *L. bidenticeps* have been collected in light traps localized between cultivated and non-cultivated fields (Rodrigues et al. 2014), while larvae are associated with corn and soybean crops (Rodrigues et al. 2011). Only *L. fusca* and *L. suturalis* (Blanchard) have been recorded as agricultural pests, mainly in the “Cerrado”, a Brazilian savannah biome. *Liogenys fusca* was found damaging corn and soybean in Mato Grosso do Sul, Mato Grosso, and Goiás States (Rodrigues et al. 2008, Santos et al. 2008, Costa et al. 2009, Ávila et al. 2014). *Liogenys suturalis* damages corn, wheat, and oats in Mato Grosso do Sul (Santos and Avila 2009). Rodrigues et al. (2016) recorded adults of *L. fusca* feeding and mating in Anacardiaceae, suggesting that these plants have an important role in the reproduction of this species. These authors cited that the native plants Urundeuva, *Myracrodruon urundeuva* (Allemão) Engl.; Aroeira-vermelha, *Schinus terebinthifolius* Raddi and Gonçalo-alves, *Astronium fraxinifolium* Schott represent an important natural source of food and reproduction, whereas the Cajueiro plant, *Anacardium occidentale* Linnaeus, represents an alternative food source as it is introduced.

Although *Liogenys* richness is huge in Argentina, research concerning species occurrence is scarce, including that related to crop pests (Mojica 2014). This might be because the first record of *Liogenys* damaging soybean plants was recent, in 2006 in Cordoba Province (Fava et al. 2008). However, there is a record from 1940 of *L. cribricollis* Moser adults feeding on leaves of Aguaribay, *Schinus molle* Linnaeus (Anacardiaceae) in Villa Maria, Córdoba (Cordo et al. 2004). Some labels on the specimens studied during this work provided information about the collection method and/or collection environment (Table 2).

**Table 2.** Species with labeled data about sampling method (type of trap/manual).

Species	Light trap	FIT	Pitfall /feces	Pitfall /no agent	Malaise	Manual or at host plants
*L. bidentata* Burmeister					X	
*L. bidenticeps* Moser	X					X
*L. bilobata* Frey	X					
*L. cartwrighti* Frey	X					
*L. corumbana* Moser	X					X
*L. densata* Frey	X					
*L. densicollis* Moser	X		X			
*L. denticeps* Blanchard			X			
*L. diodon* Burmeister		X			X	
*L. fusca* Blanchard	X		X			X
*L. hirtipennis* Frey	X			X		
*L. moseri* Frey		X	X			
*L. obscura* Blanchard	X					
*L. pallens* Blanchard	X					
*L. pallidicornis* Blanchard					X	X
*L. paralella* Frey			X			
*L. rectangula* Frey	X					
*L. rufoflava* Moser	X					
*L. santaecrucis* Blanchard	X					
*L. suturalis* (Blanchard)	X			X		X
*L. testaceipennis* Moser	X					
*L. tibialis* Moser	X					
*L. unicolor* Evans	X					
*L. vicina* Frey	X					
*L. pseudosanctaecrucis* Cherman, sp. n.	X					
*L. cavifrons* Cherman, sp. n.	X					

### Species

#### Liogenys acutidens

Taxon classificationAnimaliaColeopteraMelolonthidae

Moser, 1919

Figs 56 , 93

Liogenys acutidens Moser, 1919: 14 (orig. desc.); Blackwelder 1944: 228 (check.); Frey 1969: 47 (key); Evans 2003: 207 (check.); Evans and Smith 2005: 171 (check.); Evans and Smith 2009: 175 (check.)

##### Type material.

*Liogenys acutidens* male holotype (ZMHB): [white printed] “Brasília/ [handwritten] Cuyaba”, [white handwritten] “Liogenys/acutidens/Mos./Typen”, [light red printed] “Typus”, “Liogenys/acutidens/Mos”, [red printed] “HOLOTYPUS/ Liogenys/acutidens Moser, 1919/ labelled by MNHUB 2011”. Genitalia mounted.

##### Non-type material.

BRAZIL. MT: Cuiabá, without date and collector, 1 ex. (ZMHB); MG: Unaí (Faz. Bolívia), 22-24/X/1964, Exp. Dep. Zool. col., 1 ex. (MZSP).

##### Diagnosis.

**Figure 56.**
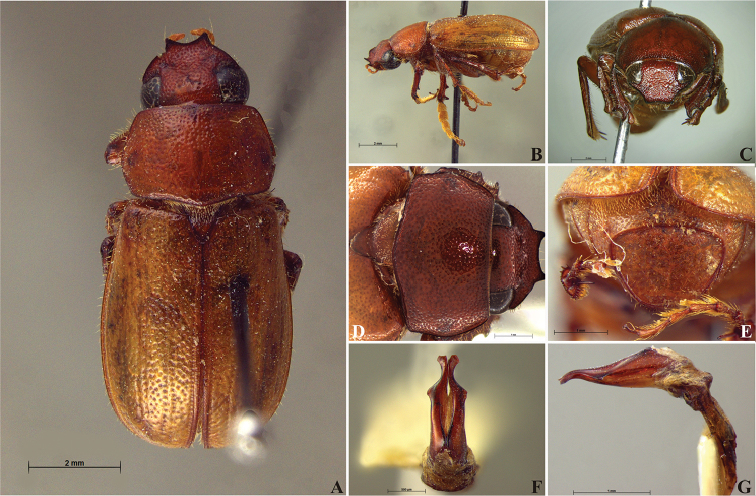
*Liogenys acutidens* Moser. **A** Dorsal view **B** Lateral view **C** Frontal view **D** Clypeus and pronotum **E** Pygidium **F** Parameres, dorsal view **G** Parameres, lateral view.

Body yellowish brown; elongate; elytra testaceous, lighter in color than pronotum; clypeal emargination wide and rounded; outer sides of anterior teeth parallel; lateral margin convex and slightly produced; distance between clypeal lateral projection and anterior tooth equal to basal width of anterior tooth, distance between clypeal lateral projection and anterior margin of eye shorter than one eye, obtuse angle between outer side of anterior teeth and clypeal lateral projection; mesotibia quadrate in cross section; pygidium convex, bristled throughout, reticulated punctures; in males, inner margins of parameres convergent; subapical projections on outer margins; parameres strongly narrowed between subapical projections and apex; apex spatula-like, sharp edges, curved outwards, not reaching the level of the parameres outer margin (Fig. 56F).

##### Redescription.

Length: 8.0–9.0 mm; width 3.7–4.2 mm. Yellowish to testaceous. *Head*: distance between eyes nearly twice the width of one eye; frons equal in length to clypeus; clypeus bristled anteriorly, emargination shallow, wide and rounded; outer sides of anterior teeth parallel; outer margin of anterior teeth shorter than the eye; lateral margin convex and slightly produced; distance between clypeal lateral projection and anterior tooth equal to the basal width of anterior tooth, distance between clypeal lateral projection and anterior margin of eye shorter than one eye, obtuse angle between outer side of anterior teeth and clypeal lateral projection; canthus not exceeding the outer margin of the eye; distal maxillary palpomere, maximum width less than twice width of apex; fovea deep, extending up to the transverse midline of the palpomere; labium transversely carinated, as wide as it is long; antenna 10-articulated, lamellae lighter in color than flagellum, and equal in length. *Thorax*: anterior margin of pronotum straight; maximum length of pronotum exceeding the length of tarsomeres I, II and III together; disc glabrous, punctures sparse and fine; pronotal posterior corners sub-angled, obtuse; proepisternum with short bristles; mesepisternum scaly, as are the sides of metasternum, also with few long bristles on the anterior margin; distance between meso- and metacoxae up to twice the metacoxa length; scutellum triangular, smooth or basally punctured at the sides. *Elytra*: shiny, glabrous, uniform yellowish to testaceous, barely lighter in color than the pronotum; elytra more than three times longer than the pronotum; elytral suture slightly darker than elytron and not elevated; all four elytral ridges barely noticeable. *Legs*: procoxa, sparse scales on infra-carinal surface, punctures visible at 12× magnification; three protibial teeth, middle and apical equal in size, distance between basal and middle teeth slightly longer than between middle and apical; protibial inner apical spur present; mesofemural disc setose, mesotibia quadrate in cross section, disc finely sculptured, two mesotibial transverse carinae, the apical one incomplete; basal apophysis of metacoxa produced beyond the outer margin of trochanter; inner margin of metatibia carinated towards apex, apical inner surface setose; metatibial disc finely sculptured; a metatibial transverse carina present posteriorly and posterior discontinuous longitudinal carina; basal metatarsomere and tarsomere II equal in size, protarsomere II short and wide; pro- and mesotarsomeres I to IV enlarged, protarsomeres slightly wider than the mesotarsomeres, less than twice as wide as the metatarsi; claw bifid, symmetrical, superior tooth narrower than the inferior and equal in length; distance between teeth shorter than the inferior tooth. *Abdomen*: ventrites bristled and on sides also scaly; propygidium visible, bristled and scaly; pygidium convex, sub-trapezoidal, wide; pygidial width not exceeding distance between spiracles of propygidium; pygidial disc bristled throughout, reticulated punctures; pygidial apex sub-quadrate. *Parameres*: width of basal region equal to the parameres together at its maximum width; parameral split at 2/3; inner margins of parameres convergent; outer margins with subapical projections; strongly narrowed between subapical projections and apex; apex spatula-like, edges sharp, curved outwards, not reaching the level of the parameral outer margin (Fig. 56F). In lateral view parameres concave; apex slightly curved downwards (Fig. 56G).

##### Type-locality.

BRAZIL, Cuiabá, Mato Grosso.

##### Geographical distribution.

BRAZIL (MT, **MG**).

##### Remarks.

*Liogenys acutidens* resembles *L. bidenticeps* (Fig. 58) in size and color, however, *L. acutidens* elytra are somewhat lighter in color; the clypeus is bristled anteriorly; the clypeal teeth are sharper and slightly longer-, the lateral margin of clypeus is slightly projected; the meso- and metatibia are quadrate in cross section with discs both finely sculptured; the mesotibial apical carina is incomplete; the pygidium is convex with reticulated punctures on disc alike *L. santaecrucis* (Fig. 71) and protarsomeres wider. Female of *L. acutidens* remains unknown.

#### Liogenys bidentata

Taxon classificationAnimaliaColeopteraMelolonthidae

Burmeister, 1855

Figs 57 , 89

L. bidentata Burmeister, 1855:13 (orig. desc.); Blackwelder 1944: 227 (check.); Evans 2003: 207 (check.); Evans and Smith 2005: 171 (check.); Evans and Smith 2009: 175 (check.)
L. bidentatus : Harold 1869a: 1140 (check.); Dalla Torre 1913: 319 (check.); Frey 1969: 43 (key).

##### Type material.

*Liogenys bidentata* male syntype (MLUH): [green handwritten] “bidentata/Burm/Bras/Kll”, [white handwritten] “Type/Liogenys/bidentatus/Burm/[printed] det. G. Frey, 1967/68”, [white printed] “prof. Hüsing/Halle”. This type is here designated the **lectotype**: [white, outlined in red, printed] “LECTOTYPE/*Liogenys bidentata*/Burmeister, 1851/des. M. A. Cherman 2014.” Female syntype (MLUH): [white handwritten] “124”, [white handwritten] “Type/Liogenys/bidentatus/Burm/[printed] det. G. Frey, 1967/68”, [white printed] “prof. Hüsing/Halle”. This type is here designated as the **paralectotype** [white, outlined in red, printed] “PARALECTOTYPE/*Liogenys bidentata*/Burmeister, 1851/des. M. A. Cherman 2014.”

##### Non-type material.

BRAZIL. PA: Tapajós, without date and collector, 2 ex. (MNHN); MA: Mirador, Parque Estadual Base da Geraldina, 21/VIII/2006, F. Limeira-de-Oliveira col., 7 ex. (CEMT); CE: Beberibe, 10/XII/2002, A. Lindemberg col., 3 ex. (DZUP); PI: Teresina, 1/I/1953, A. K. Oliveira col., 5 ex. (DZUP); 3 ex. (MNRJ); 14/X/1952, A.G.A. Silva col., 1 ex.; 22/VIII/2006, without collector, 1 ex.; “CCA-UFPI”, 20/V/2008, without collector, 2 ex. (DZUP); RN: IV/1950, M. Alvarenga, 3 ex. (MNRJ); Natal, II/1958, without collector, 1 ex. (DZUP); XII/1956, Magalhães col., 1 ex. (MNRJ); 21/XII/1952, Melo col., 1 ex. (MZSP); Pedro Velho, XII/1953, F. Barros col., 1 ex. (DZUP); 3 ex. (MNRJ); Mossoró, I/1952, M. Alvarenga, 1 ex. (DZUP); Montanhas, XII/1963, without collector, 4 ex. (CEIOC); PE: without date, Weilenmann col., 1 ex. (ZMHB); Tigipió [Recife], 4/XI/1914, Zikan col., 2 ex. (CEIOC); AL: Maceió, without date and collector, 1 ex. (MNHN); SE: (1) Caninde do S Francisco “Faz. Miramar”, 8/III/2001, L. Iannuzzi col., 1 ex. (CEMT); BA: without date, G. Bondar col., 1 ex. (NHMB); Juazeiro, without date and collector, 1 ex. (MNRJ); Feira de Santana, 8/XIII/1953, C. R. Gonçalves col., 4 ex. (DZUP); 1 ex. (MNRJ); Maracás, 19/XI/1965, F. M. Oliveira col., 2 ex. (DZUP); Jacobina, XII/1941, Mangabeira col., 1 ex. (CEIOC); Paulo Affonso, “E. E. Raso da Catarina.”, 23/X/1982, without collector, 1 ex. (MNRJ); MG: Caraí, XI/1973, M. Alvarenga col., 1 ex. (CMNC); GO: Aragarças, I/1955, F. M. Oliveira col., 3 ex. (DZUP); MT: Jacaré. Parque Nacional Xingu, XI/1961, Alvarenga and Werner col., 4 ex. (DZUP); Utiariti, VII/1961, Lenko col., 2 ex. (MZSP).

##### Diagnosis.

**Figure 57.**
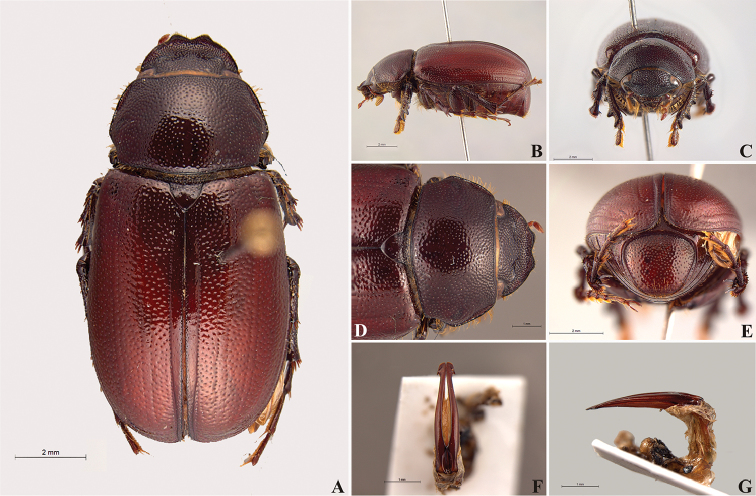
*Liogenys bidentata* Burmeister. **A** Dorsal view **B** Lateral view **C** Frontal view **D** Clypeus and pronotum **E** Pygidium **F** Parameres, dorsal view **G** Parameres, lateral view.

Body, pronotum and elytra purplish brown to dark brown, elongate; distance between eyes more than twice the width of one eye; clypeal emargination rounded or sub-angled, shallow and wide; lateral margin convex, slightly produced; pronotal posterior corners sharp, obtuse or almost right-angled; prothorax scaly posteriorly; pro-, meso- and metasternum, pro- and metacoxae with white scales; mesotibia cylindrical in cross section; protarsal claws symmetrical; metatarsomere I nearly one-half the size of tarsomere II, equal in width or slightly wider; pygidium flat, sometimes slightly convex; pygidial width exceeding distance between spiracles of propygidium; male genitalia, total length of parameres more than five times the length of their apex; inner margins convergent; apex harpoon-like with lateral angle projecting straight downward (Fig. 57F).

##### Redescription.

Length: 12.0–13.0 mm; width: 5.7–6.2. Purplish brown. *Head*: distance between eyes more than twice the width of one eye; frons equal in length to clypeus; clypeal emargination sub-angled, shallow and wide; outer sides of anterior teeth sub-parallel; outer margin of anterior teeth shorter than the eye; lateral margin convex with a barely noticeable rounded projection; canthus not exceeding the outer margin of the eye (Fig. 57D); distal maxillary palpomere, maximum width more than twice width of apex; fovea deep and oval, extending past the transverse midline of the palpomere; labium transversely carinated, as wide as it is long; antenna 10-articulated, lamellae lighter in color than flagellum and in males lamellae and flagellum equal in length. *Thorax*: anterior margin of pronotum straight, flanged throughout; maximum length of pronotum exceeding the length of tarsomeres I, II and III together; disc glabrous, punctures coarse and sparse; sides distinctly convex, pronotal posterior corners sharp, obtuse or almost right-angled; prothorax scaly posteriorly; proepisternum with long bristles; mesepisternum scaly, as are the sides of metasternum; also with few long bristles on the anterior margin; distance between meso- and metacoxae up to twice the metacoxal length; scutellum ogival, coarsely and sparsely punctured, mainly at the base (Fig. 57D). *Elytra*: shiny, glabrous, uniform dark brown to purplish; elytra more than three times longer than the pronotum; elytral suture and elytron unicolored, distinctly elevated; the two pairs of inner ridges more noticeable than the two outer pairs (Fig. 57A). *Legs*: procoxa scaly on infra-carinal and outer surface; punctures visible at 12× magnification; three protibial teeth, middle and apical equal in size, basal and middle teeth more spaced than middle and apical; inner apical spur present (Fig. 57C); mesocoxa with a tuft of long bristles; mesofemural disc setose, with a row of long bristles on the anterior margin; mesotibia cylindrical in cross section; disc coarsely sculptured, two mesotibial transverse carinae, the apical one complete; basal apophysis of metacoxa produced beyond the outer margin of trochanter; metatibia with apical spurs of different lengths, the longest equal in length to the diameter of the tibial apex; inner margin of male metatibia carinated towards apex, apical inner surface setose; metatibial disc coarsely sculptured; two metatibial transverse carinae present posteriorly; basal metatarsomere nearly one-half the length of tarsomere II and equal in width, in males protarsomere II long; pro- and mesotarsomeres I to IV enlarged, protarsomeres slightly wider than the mesotarsomeres and more than twice as wide as metatarsi; claw bifid, symmetrical, superior tooth longer and narrower than the inferior; distance between teeth as long as the inferior tooth. *Abdomen*: band of scales visible at the lowest magnification beneath the outer margin of elytra; ventrites bearing short bristles on disc and scarce scales on sides; propygidium visible, with sparse short bristles; pygidium flat, sometimes slightly convex; sub-quadrate, wider than long; pygidial width exceeding distance between propygidium spiracles; pygidial disc bristled only on apex; pygidial apex in males quadrate (Fig. 57E). *Parameres*: basal region narrower than the parameres together at the maximum width, parameral split at 2/3; total length of parameres more than five times the length of their apex; inner margins convergent; apex harpoon-like, with lateral angle projecting straight downward (Fig. 57F). In lateral view parameres straight, coplanar with basal region (Fig. 57G).

##### Type-locality.

BRAZIL. São Paulo, Ipanema [Iperó].

##### Geographical distribution.

BRAZIL (**PA**, **MA**, **CE**, **PI**, **RN**, **PE**, **AL**, **SE**, **BA**, **MG**, SP, **GO**, **MT**).

##### Remarks.

*Liogenys bidentata* resembles *L. fusca* (Fig. 64) and *L. pallidicornis* (Fig. 67) and differs from them by the pronotum being coarser and more sparsely punctured, scales on prothorax posteriorly less abundant; mesotibia always cylindrical in cross section and pygidium mostly flat. The shape of parameres is also distinctive.

#### Liogenys bidenticeps

Taxon classificationAnimaliaColeopteraMelolonthidae

Moser, 1919

Figs 58 , 92

Liogenys bidenticeps Moser, 1919: 13 (orig. desc.); Blackwelder 1944: 227; (check.), Frey 1969: 43 (key); Evans 2003: 207 (check.); Evans and Smith 2005: 171 (check.).
Liogenys bicuspis Moser, 1919: 14 (orig. desc.); Blackwelder 1944: 227; (check.), Frey 1969: 43 (key); Evans 2003: 207 (check.); Evans and Smith 2005: 171 (check.); Evans and Smith 2009: 175 (check.) **Syn. n.**

##### Type material.

*Liogenys bidenticeps* male syntype (ZMHB) [white printed] “Brasilia/ [handwritten] Sao Paulo”, [white handwritten] “Liogenys/bidenticeps/Typen Mos”, [light red printed] “Typus”, [red printed] “SYNTYPUS/Liogenys/bidenticeps Moser, 1919/labelled by MNHUB 2011”. Genitalia mounted. This type is here designated as the **lectotype** [white, outlined in red, printed] “LECTOTYPE/*Liogenys bidenticeps*/Moser, 1919/des. M. A. Cherman 2012.” Two male and a female syntypes (ZMHB): [white printed] “Brasilia/[handwritten] Sao Paulo”, [white handwritten] “Liogenys/bidenticeps/ Typen Mos”, [light red printed] “Typus”, [red printed] “SYNTYPUS/ Liogenys/ bidenticeps Moser, 1919/labelled by MNHUB 2011”. These three syntypes are here designated as **paralectotypes**, each one with the label: [white, outlined in red, printed] “PARALECTOTYPE/*Liogenys bidenticeps*/Moser, 1919/ des. M. A. Cherman 2012”. Male genitalia mounted.

*Liogenys bicuspis* male syntype (ZMHB): [white printed] “Brasilia/[handwritten] Cuyaba”, [white handwritten] “Liogenys/bicuspis/Type M Mos”, [red printed] “Typus”, [red printed] “SYNTYPUS/Liogenys/bicuspis Moser, 1919/labelled by MNHUB 2011”. Genitalia mounted. This type is here designated the **lectotype** [white, outlined in red, printed] “LECTOTYPE/*Liogenys bicuspis*/Moser, 1919/des. M. A. Cherman 2012”. Female syntype of *L. bicuspis*: [white printed] “Brasilia/[handwritten] Cuyaba”, [white handwritten] “Liogenys/bicuspis/Type F Mos”, [red printed] “Typus”, [red printed] “SYNTYPUS/Liogenys/bicuspis Moser, 1919/labelled by MNHUB 2011”. These syntype is here designated as **paralectotype** [white, outlined in red, printed] “PARALECTOTYPE/ *Liogenys bicuspis*/Moser, 1919/des. M. A. Cherman 2012”.

##### Non-type material.

BRAZIL. BA: Encruzilhada, 15°34'35"S, 40°56'51"W, 15/XII/2012, 850 m, Rafael and Grossi col., 1 ex. (INPA); SP: São Paulo, IX/1953, Vespasiano col., 1 ex. (DZUP); without date and collector, 8 ex. (ZMHB); Botucatu, 17/IX/1963, Maritovan col., 1 ex. (IBSP); Campinas, without date and collector, “Alwine Braatz V. collection”, 3 ex. (ZMHB); MT: Cuiabá, without date and collector, 4 ex. (ZMHB); 15/XI/2008, Monteiro col., 1 ex.; Fazenda Nirvana, 1/X/1988, Serrano col., 1 ex. (CEMT); “Córrego Brigadeiro [tributary of rio Jauru], próximo a Figueiropolis” [Figueiropolis D’oeste], 29/IX/1984, Binda col. 2 ex., (INPA), Jacaré, PN Xingú, XI/1947, Sick col., 1 ex. (MNRJ); MS: (1) Batayporã, 22°18'00"S, 53°15'98"W,13/X/2011, 328 m, M.G.C. Pereira (MuBio); Corumbá, without date and collector, 3 ex. (ZMHB); Guia Lopes da Laguna, 15/XI/2007, A. Abot col, 2 ex. (DZUP); Porto Murtinho, XI/1929, Melim col., 30 ex. (MNRJ); Rio Brilhante, 21-28,X/1970, V. O. Becker col., 3 ex. (DZUP); “Salobra, Miranda”, 24/X/1938, Lane col. 1 ex. (MZSP); Paraná, Jaguariaíva, 24-29/XII/1970, F. Giacomel col., 1 ex. (DZUP); RS: Catuipé, 25-30/XII/2010, F. L. dos Santos, 10 ex. (DZUP); (1) "S, Brazil”, without date and collector, 1 ex. (MNHN). PARAGUAY. IT: “Hohenau [Campo Angelo]”, 11/XI/1944, Jacob. Col., 1 ex. (SDEI); SP: “San Estanislao [Santani]”, without date and collector, 1 ex.; Thieme col., without locality, and collector, 6 ex. (ZMHB). ARGENTINA. FO: Ciudad de Formosa, XII/1949, A. Martinez col. (MZSP); Laguna Yema, Reserva Teuquito, 24°21'20,95"S, 61°18'55,23"W, 11/XII/2008, F. Ocampo, G. San Blas, Campon cols. 2 ex.; Ing. Juarez, 24°05'27"S, 61°56'49"W, 13/XII/2008, F. Ocampo, G. San Blas, Campon cols., 2 ex. (IADIZA); CA: Ancasti, 28°52'57"S, 65°25'04"W, 18/XII/2009, 573 m, F. Ocampo, Dominguez, Campon cols., 2 ex. (IADIZA); SE: Quimili, 9/XI/1939, Biraben, Bezzi Cols., 2 ex. (MLPA).

##### Diagnosis.

**Figure 58.**
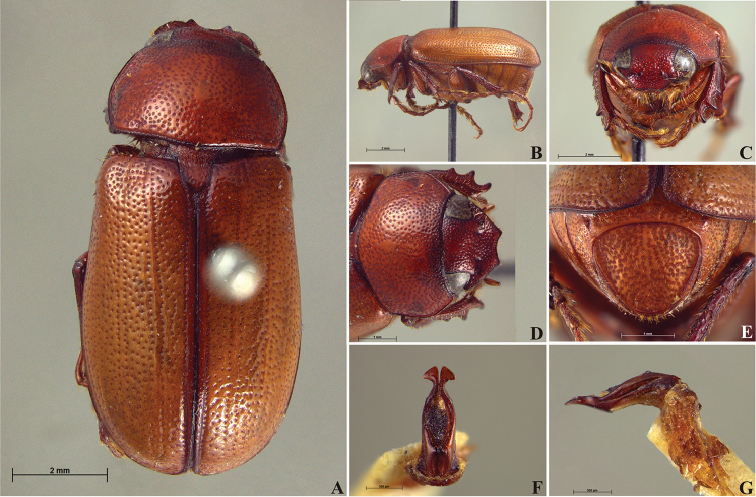
*Liogenys bidenticeps* Moser. **A** Dorsal view **B** Lateral view **C** Frontal view **D** Clypeus and pronotum **E** Pygidium **F** Parameres, dorsal view **G** Parameres, lateral view.

Body brownish; elongate; elytra light brown, lighter in color than pronotum; clypeal emargination rounded and wide; outer sides of anterior teeth sub-parallel; lateral margin convex, rounded; mesotibia cylindrical to sub-quadrate in cross section; pygidium flat, bristled throughout. In males, inner margins of parameres convergent; subapical projections on outer margins; strongly narrowed before the apex; apex spatula-like, edges sharp, curved outwards, reaching the level of the parameral outer margin (Fig. 58F).

##### Redescription.

Length: 8.5–10.7 mm; width: 4.1–5.2 mm. Brownish. *Head*: distance between eyes nearly twice the width of one eye; frons equal in length to clypeus; clypeal emargination rounded, shallow and wide; outer sides of anterior teeth sub-parallel or sometimes follow the margin of clypeus; outer margin of anterior teeth shorter than the eye; clypeal lateral margin convex but sometimes straight; canthus not exceeding the outer margin of the eye; distal maxillary palpomere, maximum width less than twice width of apex; fovea shallow, extending to the transverse midline of the palpomere; labium transversely carinated, as wide as it is long; antenna 10-articulated, lamellae lighter in color than flagellum, in males lamellae and flagellum equal in length. *Thorax*: anterior margin of pronotum slightly produced medially; maximum length of pronotum exceeding the length of tarsomeres I, II and III together; disc glabrous, punctures sparse and fine; pronotal posterior corners sub-angled, obtuse; proepisternum with short bristles; mesepisternum scaly; sides of metasternum with long bristles and scales; distance between meso- and metacoxae up to twice the metacoxa length; scutellum rounded, coarsely punctured. *Elytra*: shiny, glabrous, testaceous to brownish, uniform; elytra more than three times longer than the pronotum; elytral suture slightly darker than elytron and distinctly elevated, two pairs of inner ridges more noticeable than the two outer pairs. *Legs*: procoxa scaly on infra-carinal and outer surface; punctures visible at 12× magnification; three protibial teeth, middle and apical equal in size; distance between basal and middle teeth shorter than between middle and apical; protibial inner apical spur present; mesofemural disc setose, with a row of long bristles on anterior and posterior margins; mesotibia cylindrical in cross section, disc coarsely sculptured; two mesotibial transverse carinae, the apical one complete; basal apophysis of metacoxa produced beyond the outer margin of trochanter; metatibial apical spurs equal in length, shorter than the diameter of the tibial apex; inner margin of male metatibia carinated towards apex, apical inner surface setose; disc coarsely sculptured, two metatibial transverse carinae present posteriorly; basal metatarsomere shorter than tarsomere II, in males protarsomere II long; pro- and mesotarsomeres I to IV enlarged, protarsomeres wider than the mesotarsomeres, less than twice as wide as metatarsi; claw bifid, symmetrical, superior tooth narrower than the inferior and equal in length; distance between teeth shorter than the inferior tooth. *Abdomen*: band of scales visible at the lowest magnification beneath the outer margin of elytra; ventrites bristled on disc and sides; propygidium visible, bristled; pygidium flat, sub-trapezoidal, wide; pygidial width not exceeding distance between spiracles of propygidium; pygidial disc bristled throughout; in males pygidium wider and apex more rounded. *Parameres*: width of basal region equal to the parameres together at its maximum width, parameral split at 2/3; inner margins strongly convergent; outer margins with subapical projections; strongly narrowed before the apex; apex spatula-like, edges sharp, curved outwards, reaching the level of the parameral outer margin (Fig. 58F). In lateral view parameres curved upwards sub-basally and curved downwards partially on apex (Fig. 58G).

##### Type-locality.

*L. bidenticeps*: BRAZIL. São Paulo; *Liogenys bicuspis*: BRAZIL. Cuiabá, Mato Grosso.

##### Geographical distribution.

BRAZIL (MT, MS, **BA**, SP, **PR**, RS); **PARAGUAY (IT**, **SP)**; **ARGENTINA (FO**, **CA**, **SE)**.

##### Remarks.

*Liogenys bidenticeps* resembles *L. acutidens* in the brownish color and elytra light brown to testaceous (Fig. 56). Those species are also closely related (Cherman et al. 2016). *Liogenys bidenticeps* differs from *L. acutidens* by the following characters: clypeal lateral margin convex but not produced; clypeus glabrous; scutellum rounded apically and more punctured; elytral suture distinctly elevated; two mesotibial transverse carinae, the apical one complete; in females the pygidium is oblique in lateral view. Frey (1969) inferred that *L. bicuspis* and *L. bidenticeps* are synonymous, by writing the word “synonym” followed by a question mark (?). After studying the primary types of *L. bidenticeps* (ZMHB) and *L. bicuspis* (ZMHB) we found slight differences in the diameter of the punctures of the pygidium and the parameral length in males. When studying all the non-type material, which extends from southernmost Brazil, Paraguay and northern Argentina, we concluded that *L. bicuspis* is a junior subjective synonym of *L. bidenticeps* and those differences might represent variation among populations. Other variations among *L. bidenticeps* individuals are the color of elytra from testaceous to brownish; the clypeal anterior teeth, which outer margins vary from sub-parallel to following the margin of clypeus; clypeal lateral margin from convex to straight and in males the parameres in lateral view, more or less curved subapically.

#### Liogenys bilobata

Taxon classificationAnimaliaColeopteraMelolonthidae

Frey, 1969

Figs 59 , 89

Liogenys bilobatus Frey, 1969: 45, 58 (orig. desc., key)
Liogenys bilobata : Evans 2003: 207 (check.); Evans and Smith 2005: 178 (check.); Evans and Smith 2009: 176 (check.).

##### Type material.

*Liogenys bilobata* male holotype (MZSP): [white, printed] “Buritis (Primeira./cachoeira Rio Uru-/cuia) MG - 2-4.XI./1964 Exp. Dep. Zool.”, [white handwritten] “Type/Liogenys/bilobatus M/[printed]det. G. Frey, 1967/8/n.sp.”, [red printed] “Typus”. Genitalia mounted. Paratypes (1): Male paratype (MZSP) [white printed] “Buritis (Primeira./cachoeira Rio Uru-/cuia) MG - 2-4.XI./1964 Exp. Dep. Zool.”, [white handwritten] “Type/Liogenys/bilobatus M/[printed]det. G. Frey, 1967/8/n.sp.”.

##### Non-type material.

BRAZIL. PB: Juazeirinho, 16/III/1956, Silva col., 1 ex. (MNRJ); MT: Cuiabá, Dist. Guia. Faz. Santhidi, 15°28'47"S 56°07'33"W, 3/XI/2010, 180 m, L. Silva col., 1 ex.; Chapada dos Guimaraes, 3/XII/2008, A. F. Silva col., 1 ex.; 15°19'49"S, 55°51'08"W, 14/XII/2012, 300 m, G. Daniel col., 1 ex. (CEMT); DF: Planaltina (Embrapa Cerrados), 15°36'16"S, 47°44'16"W, 3/XI/2006, C. Oliveira col., 2 ex. (CEMT); MG: Buritis (Primeira cachoeira Rio Uricuia), 2-4/XI/1964, Exp. Dep. Zool. col., 2 ex.; Três Marias, 21/X/1964, Exp. Dep. Zool. col., 1 ex.; Unaí (Faz. Bolívia), 22-24/X/1964, Exp. Dep. Zool. col., 1 ex. (MZSP); SP: without date and collector, 1 ex. (ZMHB).

##### Diagnosis.

**Figure 59.**
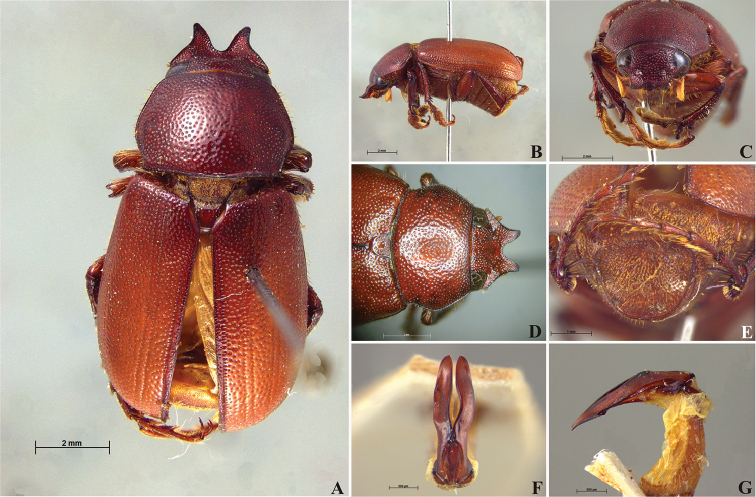
*Liogenys bilobata* Frey. **A** Dorsal view **B** Lateral view **C** Frontal view **D** Clypeus and pronotum **E** Pygidium **F** Parameres, dorsal view **G** Parameres, lateral view.

Body reddish brown; elongate, wider in the posterior third; elytra brownish, pronotum darker; clypeal emargination deep, narrow and rounded; outer sides of anterior teeth concave, following the lateral margin of clypeus; canthus exceeding the outer margin of the eye; meso- and metatibia quadrate or sub-quadrate in cross section, metafemur with thick and erect bristles on posterior margin; pygidial width exceeding distance between spiracles of propygidium; pygidial disc scaly throughout; female pygidium wider than in males. Male genitalia, parameres slightly asymmetrical; basal region narrower than the parameres together at its maximum width; apex rounded (Fig. 59F).

##### Redescription.

Length: 10.5–12.0 mm; width: 5.7–6.5 mm. Reddish brown. *Head*: distance between eyes nearly twice the width of one eye; frons equal in length to clypeus; clypeal emargination deep, narrow and rounded; clypeal teeth longer in males; outer sides of anterior teeth sub-parallel, slightly divergent; outer margin of anterior teeth longer than the eye; clypeal lateral margin concave; canthus exceeding the outer margin of the eye; distal maxillary palpomere, maximum width twice width of apex; fovea deep, extending past the transverse midline of the palpomere; labium transversely carinated, as wide as it is long; antenna 10-articulated, lamellae lighter in color than flagellum, in males lamellae and flagellum equal in length. *Thorax*: anterior margin of pronotum straight; maximum length of pronotum exceeding the length of tarsomeres I, II and III together; disc glabrous, punctures coarse and dense, coarser in females; pronotal posterior corners sharp, obtuse-angled; proepisternum with short bristles; pro- and mesepisternum scaly, as are the sides of metasternum; distance between meso- and metacoxae up to twice longer than the metacoxa; scutellum ogival to triangular, coarsely punctured, in males at the base. *Elytra*: shiny, glabrous, uniform reddish brown; elytra more than three times longer than the pronotum; elytral suture and elytron unicolored, not elevated; all four elytral ridges barely noticeable. *Legs*: procoxa scaly on infra-carinal and outer surface; punctures visible at 12× magnification; three protibial teeth, middle and apical equal in size, the three teeth equally spaced; protibial inner apical spur present; mesofemural disc setose; mesotibia quadrate in cross section, mesotibial disc coarsely sculptured; two mesotibial transverse carinae, the apical one incomplete in males; basal apophysis of metacoxa produced beyond the outer margin of trochanter; metafemur with thick and erect bristles on posterior margin; metatibia with posterior discontinuous longitudinal carina; metatibial apical spurs of different lengths, the longest one shorter than the diameter of the tibial apex; inner margin of male metatibia carinated towards apex; inner surface setose; disc coarsely sculptured; metatibia not transversally carinated; basal metatarsomere equal to or slightly shorter than tarsomere II and as wide as; in males protarsomere II short and wide; in males pro- and mesotarsomeres I to IV enlarged, protarsomeres slightly wider than the mesotarsomeres; more than twice as wide as metatarsi; claw bifid, symmetrical, superior tooth longer and slightly narrower than the inferior; distance between teeth shorter than the inferior tooth. *Abdomen*: band of abundant scales visible at the lowest magnification beneath the outer margin of elytra; ventrites with short and long bristles and scales on disc and sides, propygidium visible, scaly; pygidium flat in lateral view, sub-quadrate, wide; pygidial width exceeding distance between spiracles of propygidium; pygidial disc setose throughout, with scales mainly; pygidial apex in males sub-quadrate. *Parameres*: basal region narrower than the parameres together at its maximum width, parameral split at 2/3; parameres slightly asymmetrical; inner margins slightly convex; apical edge rounded, apex shape indistinct from the rest of the paramere (Fig. 59F).

##### Type-locality.

BRAZIL. Minas Gerais, Buritis (Primeira Cachoeira Rio Urucuia).

##### Geographical distribution.

BRAZIL (**PB**, **MT**, **DF**, MG, **SP**).

##### Remarks.

There is one specimen in MZSP with paratype label, but it was collected from “Minas Gerais, Unaí, Faz. Bolívia”. Since the unique type-locality mentioned in the original description is “Minas Gerais, Buritis (Primeira Cachoeira Rio Urucuia)”, this specimen is not part of the type series, so it has no nomenclatural value. *Liogenys bilobata* resembles *L. diodon* (Fig. 62) but differs in the size; lateral margin of clypeus being more concave (Fig. 20); canthus exceeding the outer margin of the eye; pronotal posterior corners sharp; abundant yellow scales on metasternum and sides of abdomen and throughout the pygidium, pygidial apex angled in males; metatibial disc more coarsely sculptured; metatibial outer longitudinal carina well defined in males and basal metatarsomere (I) as long as metatarsomere II.

#### Liogenys concolor

Taxon classificationAnimaliaColeopteraMelolonthidae

Blanchard, 1851

Figs 60 , 91

Liogenys concolor Blanchard, 1851: 167 (orig. desc.); Harold 1869a: 1140 (check.); Dalla Torre 1913: 318 (check.); Blackwelder 1944: 227 (check.); Frey 1969: 39, 54 (key, redescription); Evans 2003: 207 (check.); Evans and Smith 2005: 172 (check.); Evans and Smith 2009: 176 (check.).
Liogenys obesa Burmeister, 1855: 15 (orig. desc.); Blackwelder 1944: 227 (check.); Evans 2003: 211 (check.); Evans and Smith 2005: 175 (check.); Evans and Smith 2009: 176 (check.) **Syn. n.**
Liogenys obesus : Harold 1869a: 1140 (check.); Dalla Torre 1913: 318 (check.); Frey 1969: 41 (key).

##### Type material.

*Liogenys concolor* female syntype (MNHN): [white handwritten] “Campos/Geraes”, [light green printed] “MUSÉUM PÁRIS/ [handwritten] Campos”, [red printed] “HOLOTYPE”, [green handwritten] “L. concolor/ Cat Mus/ Brésil/ M. A. Saint Hilaire”. As in the original description figures a range of length “9-10 mm”, this type is here designated the **lectotype**: [white, outlined in red, printed] “LECTOTYPE/*Hilarianus concolor*/Burmeister, 1855/des. M. A. Cherman 2017”.

*Liogenys obesa* male syntype MLUH: [green handwritten] “obesa Burm/Bras. Br.”, [white handwritten] “115 Koll”, [white printed] “det. G. Frey, 1967/68/ [handwritten] Liogenys/obesus Burm/type”, [white printed] “Prof. Hüsing Halle”. This type is here designated the **lectotype**: [white, outlined in red, printed] “LECTOTYPE/*Liogenys obesa*/Burmeister, 1855/des. M. A. Cherman 2014”. Male syntype of *L. obesa* (MLUH): [white printed] “det. G. Frey, 1967/68/ [handwritten] Liogenys/obesus Burm/type”, [white printed] “Prof. Hüsing Halle”. This type is here designated the **paralectotype** [white, outlined in red, printed] “PARALECTOTYPE/*Liogenys obesa*/Burmeister, 1855/des. M. A. Cherman 2014”.

##### Non-type material.

BRAZIL. SP: São Paulo, without date and collector, 1 ex. (ZMHB); PR: Guarapuava, I/1959, Schneider col., 1 ex. (MNRJ); Ponta Grossa, “Lageado Campo”, I/1947, without collector, 2 ex. (DZUP). ARGENTINA. MI: Loreto, II/1960, without collector, 3 ex. (NHMB).

##### Diagnosis.

**Figure 60.**
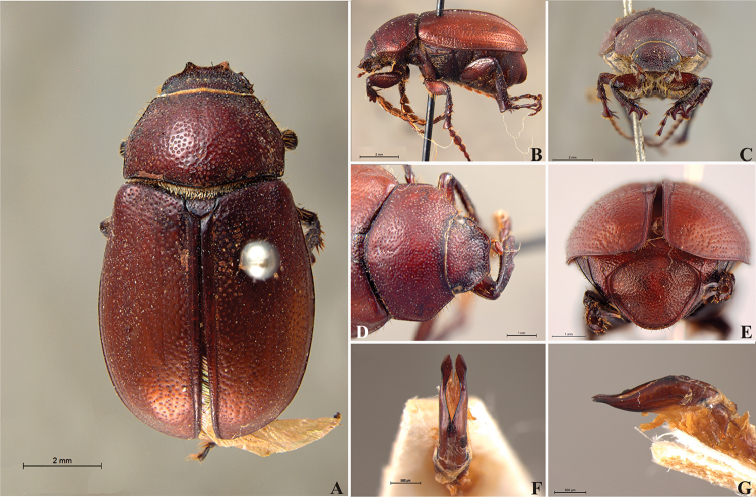
*Liogenys concolor* Blanchard. **A** Dorsal view **B** Lateral view **C** Frontal view **D** Clypeus and pronotum **E** Pygidium **F** Parameres, dorsal view **G** Parameres, lateral view.

Body and pronotum dark purplish red, head and pronotum darker anteriorly (Fig. 60A, D); elytra purplish red, semiopaque; body short and oval; distance between eyes more than five times wider than one eye; clypeal emargination shallow, rounded and very wide; lateral margin convex; lamellae of antenna darker and shorter than the flagellum; pronotal disc punctures strongly coarse, pronotal posterior corners sharp, obtuse-angled and slightly produced; distance between meso- and metacoxae as long as the metacoxa; scutellum wide; elytra more convex dorsoventrally (Fig. 60B), up to three times longer than the pronotum; all four elytral ridges noticeable; metafemur with abundant thick and erect bristles on posterior margin; male metafemur medially produced on posterior margin and inner margin of metatibia abruptly sub-basally produced towards apex; pygidium convex, large, with reticulated punctures; pygidial width exceeding distance between spiracles of propygidium; parameres, inner margins convergent; narrowed subapically; apex lanceolate (Fig. 60F).

##### Redescription.

Length: 10.0–11.0 mm; width: 5.9–6.4 mm. Purplish red. *Head*: distance between eyes more than five times wider than one eye; frons equal in length to clypeus; clypeal emargination rounded, shallow and very wide; clypeal lateral margin convex; outer sides of anterior teeth sub-parallel; outer margin of anterior tooth shorter than one eye; canthus exceeding the outer margin of the eye; distal maxillary palpomere oval, maximum width less than twice width of apex; fovea shallow and short, not reaching the transverse midline of the palpomere; labium transversely carinated, as wide as it is long; antenna 10-articulated, lamellae darker in color and shorter than the flagellum. *Thorax*: anterior margin of pronotum slightly produced medially; maximum length of pronotum exceeding the length of tarsomeres I, II and III together; disc glabrous, punctures sparse and strongly coarse; posterior corners sharp and slightly produced, obtuse-angled; proepisternum with long bristles; mesepisternum scaly, as are the sides of metasternum; distance between meso- and metacoxae as long as the metacoxa; scutellum wide, rounded, coarsely punctured, sometimes darker than pronotum. *Elytra*: semi-opaque, glabrous, uniform purplish red; barely convex dorsoventrally; elytra less than three times longer than the pronotum; elytral suture and elytron unicolored, distinctly elevated; all four elytral ridges noticeable. *Legs*: procoxa scaly on infra-carinal and outer surface; punctures visible at 12× magnification; three protibial teeth, middle and apical equal in size, the three teeth equally spaced; protibial inner apical spur present; mesofemural disc setose, with a row of long bristles on anterior and posterior margins; mesotibia cylindrical; disc coarsely sculptured, two mesotibial transverse carinae, the apical complete; basal apophysis of metacoxa produced beyond the outer margin of trochanter; abundant thick and erect bristles on posterior margin; male metafemur medially produced on posterior margin; metatibial apical spurs equal in length, length equal to the diameter of the tibial apex; inner margin of male metatibia carinated and abruptly sub-basally produced towards apex; apical inner surface setose; metatibial disc coarsely sculptured; metatibial transverse carina present posteriorly; basal metatarsomere slightly shorter than tarsomere II and equally wide, in males protarsomere II as wide as it is long; pro- and mesotarsomeres I to IV enlarged, protarsomeres slightly wider than the mesotarsomeres and more than twice as wide as metatarsi; claw bifid, symmetrical, superior tooth longer and narrower than the inferior; distance between teeth longer than the inferior tooth. *Abdomen*: band of abundant scales visible at the lowest magnification beneath the outer margin of elytra; ventrites bristled on disc and sides; propygidium slightly visible, scaly; pygidium convex, sub-trapezoidal, wide, pygidial width exceeding distance between spiracles of propygidium; pygidial disc bristled on apex; reticulated punctures; pygidial apex in males sub-quadrate. *Parameres*: width of basal region equal to the parameres together at its maximum width, parameral split at 2/3; inner margins of parameres convergent; narrowed subapically; apex lanceolate (Fig. 60F). In lateral view parameres concave (Fig. 60G).

##### Type-locality.

*Liogenys concolor*: BRAZIL. Campos Gerais [Paraná state]; *Liogenys obesa*: BRAZIL. Irisanga [Orissanga, São Paulo state].

##### Geographical distribution.

BRAZIL (SP, PR, RS); **ARGENTINA (MI)**.

##### Remarks.

*Liogenys concolor* is the only species among the entire genus with short body, due to the elytra convex dorsoventrally and distance between meso- and metacoxae as long as the metacoxa; distal maxillary palpomere oval; lamellae and flagellum almost black, darker than the scape; pronotal posterior corners slightly produced and scutellum wide (Fig. 60A). This species shares those features with *Homalochilus niger* although they are not closely related species (Cherman et al. 2016). As *L. concolor* bears a combination of features that are present only in *Liogenys*, like teeth on clypeus and umbilicate punctures of pygidium disc, the position of this species remains in *Liogenys* (Cherman et al. 2016). Primary types of *Liogenys concolor* (MNHN) and *Liogenys obesa* (MLUH) were studied and we concluded that they are conspecific, being *Liogenys obesa* the **junior subjective synonym** of *Liogenys concolor*. The holotype of *L. concolor* is a female from “Campos Gerais” (Central Paraná State, Brazil) and the lectotype of *L. obesa* is a male from “Brazil”. The association between male and female, to confirm that they are conspecific, was possible through the study of males and females from the same sample collected in Ponta Grossa, Brazil. Furthermore, Ponta Grossa is a municipality localized at “Campos Gerais” region.

#### Liogenys corumbana

Taxon classificationAnimaliaColeopteraMelolonthidae

Moser, 1921

Figs 61 , 93

Liogenys corumbanus Moser, 1921b: 139 (orig. desc.); Frey 1969: 47 (key).
Liogenys corumbana : Blackwelder 1944: 227 (check.); Evans 2003: 207 (check.); Evans and Smith 2005: 172 (check.); Evans and Smith 2009: 176 (check.).

##### Type material.

*Liogenys corumbanus* male syntype (ZMHB): [white printed] “Corumba/Matt. Grosso”, [white handwritten] “Liogenys/corumbanus/Mos/Typen m#.”, [light red printed] “Typus”, [white printed] “Liogenys/corumbanus/Mos.”, [red printed] “Syntypus/Liogenys/ corumbana Moser, 1921/labelled by MNHUB 2011”. Genitalia mounted. This type is here designated the **lectotype**: [white, outlined in red, printed] “LECTOTYPE/*Liogenys corumbanus*/Moser, 1921/des. M. A. Cherman 2012”. Seven males and two female syntypes of *L. corumbana* (ZMHB): [white printed] “Corumba/Matt. Grosso”, [white handwritten] “Liogenys/corumbanus/Mos/Typen”, [light red printed] “Typus”, [white printed] “Liogenys/corumbanus/Mos.”, [red printed] “Syntypus/Liogenys/ corumbana Moser, 1921/labelled by MNHUB 2011”. These nine syntypes are here designated **paralectotypes**, each one with the label: “PARALECTOTYPE/*Liogenys corumbanus*/ Moser, 1921/ des. M. A. Cherman 2012”.

##### Non-type material.

BRAZIL: MT: Campo Verde, “Fazenda Cavera”, 21/X/2011, Biava col., 1 ex. (CEMT); Comodoro, “Morro do sem Boné”, 13°43'54"S, 60°18'40"W, X/2012, M.F. Souza col., 1 ex. (CEMT); Cuiabá, Boa Esperanza, X/2011, F. Vaz-de-Mello col., 1 ex. (CEMT); without date and collector, 1 ex. (ZMHB); Poconé, “SESC Pantanal”, 8/XII/2003, without collector, 1 ex. (CEMT); Varzea Grande, 13/V/2009, Friozzo col., 1 ex. (CEMT); “VBS Trindade [Vila Bela da Santíssima Trindade] - Rio Guaporé”, 30/IX-1/X/1984, Binda col., 72 ex. (INPA); “Vila Bela [Vila Bela da Santíssima Trindade] - Margem Guaporé”, 1-30/IX/1984, Marcolino col., 9 ex. (CEMT); MS: Campo Grande, X/1947, A. Maller col., 1 ex. (AMNH).

##### Diagnosis.

**Figure 61.**
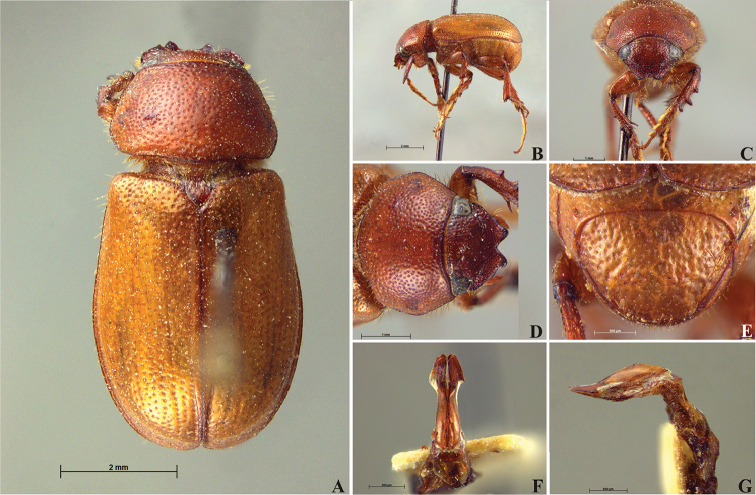
*Liogenys corumbana* Moser. **A** Dorsal view **B** Lateral view **C** Frontal view **D** Clypeus and pronotum **E** Pygidium **F** Parameres, dorsal view **G** Parameres, lateral view.

Body and elytra yellowish; elongate; pronotum and scutellum reddish yellow; clypeal emargination deep, sharp and narrow; outer sides of anterior teeth parallel; clypeal lateral margin convex, with a sharp tooth-like projection, obtuse angle between outer side of anterior teeth and clypeal lateral projection; canthus exceeding the outer margin of the eye; anterior margin of pronotum straight, flanged throughout; pronotal posterior corners obsolete (Fig. 61A); mesotibia quadrate in cross section; metafemur, thick and erect bristles on posterior margin; inner surface of metatibia glabrous; pygidium convex and wide, sub-quadrate; pygidial width exceeding distance between spiracles of propygidium; bristles only at the apex or sides; total length of parameres near three times the length of their apex; apex harpoon-like with lateral angle projecting straight downward (Fig. 61F).

##### Redescription.

Length: 7.0–8.0 mm; width: 3.7–4.2 mm. Yellowish. *Head*: distance between eyes more than twice the width of one eye, wider in females; frons equal in length to clypeus; clypeal emargination narrow, deep and sharp; outer sides of anterior teeth sub-parallel; outer margin of anterior teeth as long as the eye; lateral margin convex, with a sharp tooth-like projection; distance between this projection and anterior margin of eye longer than one eye; distance between clypeal lateral projection and anterior tooth shorter than basal width of anterior tooth; obtuse angle between outer side of anterior teeth and clypeal lateral projection; canthus exceeding the outer margin of the eye; distal maxillary palpomere, maximum width less than twice width of apex; fovea deep, extending past the transverse midline of the palpomere; labium transversely carinated, as wide as it is long; antenna 10-articulated, lamellae lighter in color than flagellum and in males lamellae longer than flagellum. *Thorax*: anterior margin of pronotum straight, flanged throughout; maximum length of pronotum exceeding the length of tarsomeres I, II and III together, disc glabrous, punctures dense and coarse; pronotal posterior corners rounded; proepisternum with short bristles; mesepisternum bristled, as are the sides of metasternum, few long bristles on the anterior margin; distance between meso- and metacoxae up to twice longer than the metacoxa; scutellum triangular, finely punctured mainly at the base. *Elytra*: shiny, glabrous, uniform yellowish; elytra more than three times longer than the pronotum; elytral suture and elytron unicolored, distinctly elevated; all four elytral ridges barely noticeable. *Legs*: procoxa, sparse scales on infra-carinal and outer surface, smooth at 12× magnification; three protibial teeth, middle and apical equal in size, distance between basal and middle teeth longer than between middle and apical; protibial inner apical spur present; mesofemural disc setose, with a row of long bristles on anterior and posterior margins; mesotibia quadrate in cross section, disc finely sculptured, two mesotibial transverse carinae, the apical one incomplete; basal apophysis of metacoxa produced beyond the outer margin of trochanter; metafemur, thick and erect bristles on posterior margin; metatibial apical spurs of different lengths, the longest equal in length to the diameter of the tibial apex, inner margin of male metatibia carinated towards apex; apical inner surface glabrous; disc finely sculptured; metatibial transverse carina present posteriorly; basal metatarsomere smaller and wider than tarsomere II, in males protarsomere II long; in males pro- and mesotarsomeres I to IV enlarged, protarsomeres slightly wider than the mesotarsomeres, less than twice as wide as metatarsi; claw bifid, symmetrical, superior tooth longer and as wide as the inferior; distance between teeth shorter than the inferior tooth. *Abdomen*: ventrites with sparse short bristles on disc and sides; propygidium slightly visible, bristled; pygidium convex, sub-quadrate, wide, pygidial width exceeding distance between spiracles of propygidium; pygidial disc bristled on apex or sides; pygidial apex rounded. *Parameres*: basal region as wide as the parameres together at its transverse midline, parameral split at 2/3; total length of parameres near three times the length of their apex; inner margins straight; apex harpoon-like with lateral angle projecting straight downward (Fig. 61F). In lateral view parameres slightly concave (Fig. 61G).

##### Type-locality.

BRAZIL.Corumbá, Mato Grosso do Sul.

##### Geographical distribution.

BRAZIL (**MT**, MS).

##### Remarks.

*Liogenys corumbana* is one of the smallest *Liogenys* species together with *L. minuta* Moser, 1924, this one from central Argentina. *Liogenys corumbana* is somewhat similar to *L. acutidens* because they share the size and color, but *L. corumbana* differs easily from the other by the shape of clypeus being deeply emarginate anteriorly and lateral margin produced sharply, forming a tooth-like.

#### Liogenys diodon

Taxon classificationAnimaliaColeopteraMelolonthidae

Burmeister, 1855

Figs 62 , 89

Liogenys diodon Burmeister, 1855: 15 (orig. desc.); Blackwelder 1944: 228 (check.); Frey 1969: 47 (key); Evans 2003: 208 (check.); Evans and Smith 2005: 178 (check.); Evans and Smith 2009: 177 (check.)
Liogenys caviceps Frey, 1964: 692 (orig. desc.); Frey 1969: 44 (syn.).

##### Type material.

*Liogenys diodon* female holotype (MLUH): [green handwritten] “diodon Br./Bras. Mlly”, [white handwritten] “Liogenys/diodon Brm/ Type F/[printed] det. G. Frey 1967/68”, [white printed] “Prof. Hüsing/Halle”. Genitalia mounted.

*Liogenys caviceps* male holotype (NHMB): [white printed] “Mossoro/R.G.N. Bras. /X.1951”, [red printed] “TYPE [handwritten] M”, [white handwritten] “Type M/ [printed] Liogenys [handwritten] diodon Burm [printed] det. G. Frey 1968”. Genitalia mounted. Paratypes (4): *L. caviceps* female paratype (NMHB): [white printed] “Mossoro/R.G.N. Bras./X.1951”, [white printed] “f#”, [white printed] “Liogenys [handwritten] diodon/F/Burm [printed] det. G. Frey 1968”. *L. caviceps* female paratype (AMNH): [white printed] “Mossoro/R.G.N. Bras./X.1951”, [white printed] “f#”, [white printed] “CUM TYPO/COMPARATUM”, [white printed] “Liogenys [handwritten] diodon/F/Burm [printed] det. G. Frey 1968”, [white handwritten] “Plectris [wrong genus] caviceps Frey/= “diodon Burm.” In NHMB another two female paratypes remain not studied.

##### Non-type material.

BRAZIL: PI: Piracuruca. Parque Nacional Sete cidades, Poço da bananeira, 4°5'56"S, 41°40'34"W, 11/II/2013,158 m, D. M. Takiya and APM Santos cols., “Pennsylvania trap”, 1 ex. (CEMT); CE: without date and collector, 1 ex. (MNRJ); RN: Jardim de Angicos, I/1952, A. Alvarenga col. 2 ex. (DZUP); Mossoró, X/1951, without collector, 2 ex. (NHMB); 1 ex. (AMNH); PE: Buique. Parque Nacional Catimbau, 8°37'S, 37°9'W, 25/I/2005, M. Schessl col. 2 ex. (CEMT); SE: Caninde do São Francisco, Fazenda Poço verde, 4/III/2000, L. Iannuzzi col. 1 ex.; (CEMT); Fazenda Miramar, 8/III/2001, L. Iannuzzi, 1 ex. (CEMT); GO: Cabeceiras, 24-27/X/1964, Exp.Dep. Zool. col. (MZSP).

##### Diagnosis.

**Figure 62.**
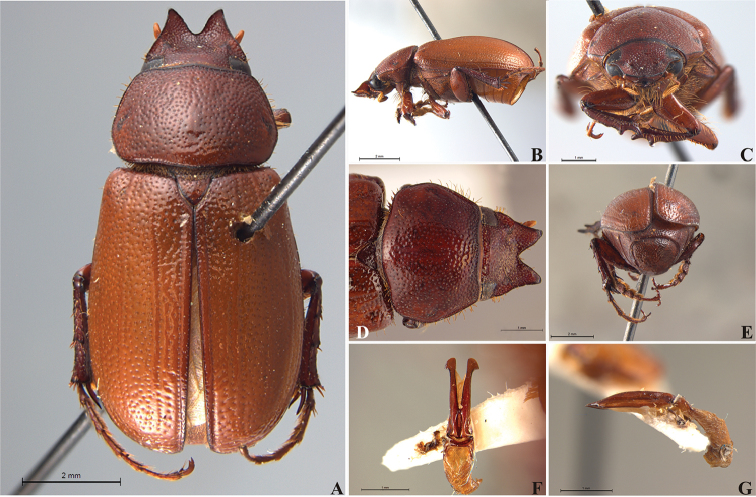
*Liogenys diodon* Burmeister. **A** Dorsal view **B** Lateral view **C** Frontal view **D** Clypeus and pronotum **E** Pygidium **F** Parameres, dorsal view **G** Parameres, lateral view.

Body reddish brown; elongate, sides almost parallel in males; elytra brownish, pronotum slightly darker; clypeal emargination deep, rounded and narrow; outer sides of anterior teeth concave, follow the lateral margin of clypeus; meso- and metatibia quadrate or sub-quadrate in cross section, metafemur with thick and erect bristles on posterior margin; pygidial width exceeding distance between spiracles of propygidium; bristles only at apex. In males, apex of pygidium more rounded; parameres; more than five times the length of their apex; apex harpoon-like with lateral angle projecting straight downward (Fig. 62F).

##### Redescription.

Length: 8.8–9.5 mm; width: 4.7–5.5 mm. Reddish brown. *Head*: distance between eyes nearly twice the width of one eye; frons shorter than clypeus; clypeal emargination narrow, deep and rounded; outer sides of anterior teeth follow the lateral margin of clypeus; lateral margin concave; canthus not exceeding the outer margin of the eye; distal maxillary palpomere, maximum width almost equal to the apex; fovea shallow, extending to or past the transverse midline of the palpomere; labium transversely carinated, as wide as it is long; antenna 10-articulated, lamellae lighter in color than flagellum, in males lamellae and flagellum equal in length. *Thorax*: anterior margin of pronotum slightly produced medially, flanged throughout; maximum length of pronotum exceeding the length of tarsomeres I, II and III together; disc glabrous, punctures sparse; pronotal posterior corners rounded (Fig. 62D); proepisternum with long bristles; mesepisternum scaly; sides of metasternum scaly and bristled; distance between meso- and metacoxae up to twice longer than the metacoxa; scutellum ogival, scarsly punctured. *Elytra*: shiny, glabrous, uniform reddish brown; elytra more than three times longer than the pronotum; elytral suture slightly darker than elytron and distinctly elevated; two pairs of inner ridges more noticeable than the two outer pairs. *Legs*: procoxa scaly on infra-carinal and outer surface; punctures visible at 12× magnification; three protibial teeth, the apical the longest, in males the three teeth equally spaced, slightly different in females; protibial inner apical spur present; anterior margin of mesofemur with a row of long bristles; mesotibia quadrate or sub-quadrate in cross section, disc finely sculptured, two mesotibial transverse carinae, in males the apical one incomplete; basal apophysis of metacoxa produced beyond the outer margin of trochanter; metafemur with thick and erect bristles on posterior margin; metatibia with posterior discontinuous longitudinal carina; metatibial apical spurs of different lengths, the longest one exceeding the diameter of the tibial apex; inner margin of male metatibia carinated towards apex; apical inner surface setose; disc finely sculptured; two metatibial transverse carinae present posteriorly; basal metatarsomere smaller than the tarsomere II and as wide as; in males protarsomere II short and wide; pro- and mesotarsomeres I to IV enlarged and more than twice as wide as metatarsi; claw bifid, symmetrical, superior tooth longer and narrower than the inferior; distance between teeth shorter than the inferior tooth. *Abdomen*: band of scales visible at the lowest magnification beneath the outer margin of elytra; ventrites bristled on disc and sides; propygidium slightly visible, bristled and scaly; pygidium convex, sub-trapezoidal, wide; pygidial width exceeding distance between spiracles of propygidium; pygidial disc scarsly bristled on apex; pygidial apex sub-rounded in males. *Parameres*: width of basal region equal to the parameres together at its transverse midline, parameral split at 2/3; total length of parameres more than five times the length of their apex; inner margins straight; apex harpoon-like with lateral angle projecting straight downward (Fig. 62F). In lateral view slightly convex (Fig. 62G).

##### Type-locality.

*Liogenys diodon*: BRAZIL (“Norte”) [Northern Brazil]; *Liogenys caviceps* (syn.): BRAZIL. Mossoro, R.G.N [Rio Grande do Norte state].

##### Geographical distribution.

BRAZIL (**PI**, **CE**, RN, **PE**, **SE**, **GO**).

##### Remarks.

*Liogenys diodon* resembles *L. bilobata* (Fig. 59), they share the deeply emarginate clypeus with lateral margin concave; clypeal teeth longer in males; protarsi wider than mesotarsi; pygidial width exceeding distance between spiracles of propygidium and thick and erect bristles on posterior margin of metafemur, this latter feature also seen in *L. sinuaticeps* (Fig. 33). *Liogenys diodon* differs from *L. bilobata* in the smaller size; clypeal lateral margin being less concave; canthus not exceeding the outer margin of the eye; pronotal posterior corners rounded; metatibia with outer longitudinal carina barely defined or absent, disc finely sculptured; metatarsomere I shorter than tarsomere II; disc of ventrites bristled instead of scaly; pygidium convex, almost glabrous with sub-rounded apex and male metafemur not medially produced on posterior margin. *Liogenys caviceps* types (NMHB) bear a label written by Frey (1964) with the name *Liogenys diodon* instead of *L. caviceps*. We suppose they are in fact the primary types as the type-locality written in the labels is exactly the same mentioned in the original description. We believe that when Frey (1969) synonymized those species, he must have swapped the original labels with the species name, leaving in the types of *L. caviceps* only the name of its senior synonym.

#### Liogenys elegans

Taxon classificationAnimaliaColeopteraMelolonthidae

Nonfried, 1891

Figs 63 , 91

Liogenis [sic] elegans Nonfried, 1891: 262 (orig. desc.).
Liogenys elegans Nonfried, 1891; Dalla Torre 1913: 318 (check.); Blackwelder 1944: 227 (check.); Frey 1969: 40 (key); Evans 2003: 208 (check.); Evans and Smith 2005: 173 (check.); Evans and Smith 2009: 177 (check.).
Liogenys brasiliensis Moser, 1919: 12 (orig. desc.); Moser 1921b: 140 (syn.)
Liogenys forsteri Frey, 1975: 260 (orig. desc.); Evans 2003: 209 (check.); Evans and Smith 2005: 173 (check.); Evans and Smith 2009: 177 (check.) **Syn. n.**

##### Type material.

*Liogenys elegans* female syntype (ZMHB): [white printed] “Coll. Nonfried/Brasilia”, [white handwritten] “26.”, [white handwritten] “Liogenys/elegans”, [red printed] “Typus”, [white printed] “Liogenys/elegans/Nonfr.”, [red printed] “SYNTYPUS/Liogenys/elegans Nonfried, 1891/labelled by MNHUB 2014”. This type is here designated the **lectotype**: [white, outlined in red, printed] “LECTOTYPE/*Liogenys elegans*/Nonfried, 1891/des. M. A. Cherman 2014”.

*Liogenys brasiliensis* male syntype (ZMHB): [white printed] “R. Grande/do Sul”, [white handwritten] “Liogenys/brasiliensis/ Mos/Typen m#.”, [white handwritten] “= elegans/ Nonfr.”, [red printed] “Typus”, [white printed] “Liogenys/brasiliensis/Mos.”, [red printed] “SYNTYPUS/Liogenys/brasiliensis Moser, 1919/labelled by MNHUB 2014”. Genitalia mounted. This type is here designated the **lectotype** [white, outlined in red, printed] “LECTOTYPE/*Liogenys brasiliensis*/Moser, 1919/des. M. A. Cherman 2014”. Female syntype (ZMHB): [white printed] “Brasília/ [handwritten] R. Grande do Sul”, [white handwritten] “Liogenys/brasiliensis/ Mos/Typen f# ”, [red printed] “Typus”, [white printed] “Liogenys/brasiliensis/Mos.”, [red printed] “SYNTYPUS/Liogenys/brasiliensis Moser, 1919/labelled by MNHUB 2014. This type is here designated the **paralectotype** [white, outlined in red, printed] “PARALECTOTYPE/*Liogenys brasiliensis*/Moser, 1919/ des. M. A. Cherman 2014”.

*Liogenys forsteri* two male paratypes (NHMB): [white printed] “Huerta Grande/ Cordoba, Argent. / 1.II.1955/ leg. H. Foerster”, [red printed] “Paratype/ [handwritten] Liogenys/ forsteri/ G.Frey 1974”.

##### Non-type material.

PARAGUAY. IT: Hohenau, IX/1929, Jacob col., 1 ex.; 22/X/1945, Jacob col., 1 ex. (SDEI); without locality, date and collector, 1 ex. (MLPA). BRAZIL. “Santa Cruz”, without date and collector, 1 ex. (NHMB); PR: Araucaria, without date, Dr. Czaki, 2 ex.; 10/XI, without collector, 1 ex. (AMNH); Banhado [Quatro Barras], XII/1972, P.J. Riehs col., 1 ex. (DZUP); Campo do Tenente, 10/X/1973, O. Mielke col., 3 ex. (DZUP); Caviúna [Rolandia], IX/1947, A. Maller col., 2 ex. (AMNH); Cerro Azul, 9/XII/1972, without collector, 1 ex. (DZUP); Curitiba, 6/XI/1969, 900 m, without collector, 1 ex.; 12/XI/1965, F. Giacomel col., 1 ex.; 17/XI/1970, without collector, 1 ex. (DZUP); XII/1911, without collector, 2 ex.(ZMHB); Guarauna, XII/1940, without collector, 1 ex. (DZUP); Marumbi, XI/1965, Laroca and Otero cols., 2 ex. (DZUP); Rio Negro, XI/1923, Wiltz col., 1 ex. (MNRJ); SC: Avencal, XII/1958, 800 m, without collector, 1 ex. (DZUP), Corupa, X/1948, A. Maller col., 1 ex. (AMNH), XII/1959, 60 m, without collector, 1 ex. (DZUP); without date, Reitter col., 1 ex. (NHMB); XII/1952, A. Maller col., 1 ex.; I/1932, Metz col., 2 ex. (MNRJ); Joinville, III/1958, Dirings col., 4 ex. (MZSP); Lages, without date, without collector, 1 ex. (ZMHB); Mafra, without date, Reitter col., 1 ex. (NHMB); XII/1937, A. Maller col., 1 ex. (MNRJ); Pinhal, XII/1951, A. Maller col., 1 ex. (MNRJ); Rancho Queimado, 8-11/X/1994, Bonaldo col., 4 ex.; 11/X/1996, Garcia col., 2 ex. (MCNZ); Rio Vermelho, IX/1945, A. Maller col., 1 ex. (AMNH); São Joaquim, 25/I/1983, P. Moure and Giacomel cols., 1 ex. (DZUP); RS: without date and collector, 5 ex. (ZMHB); Barão do Triunfo, 28/I/1996, Francschini col., 1 ex. (MCNZ); Cambará do Sul, 19/XII/1994, Moura col. 1 ex. (MCNZ); Canela,18/XI/1990, Hoffman col., 1 ex. (MCNZ); without locality, date and collector, 5 ex. (ZMHB). ARGENTINA. MI: Puerto Iguazu, 24/X/1997, without collector, 2 ex. (CEMT); IX/1947, Duret col., 2 ex. (CMNC); Panambi, XII/1958, A. Martinez col., 1 ex. (CMNC); CO: Huerta Grande, 7/I/1955, Foerster col., 1 ex. (NHMB).

##### Diagnosis.

**Figure 63.**
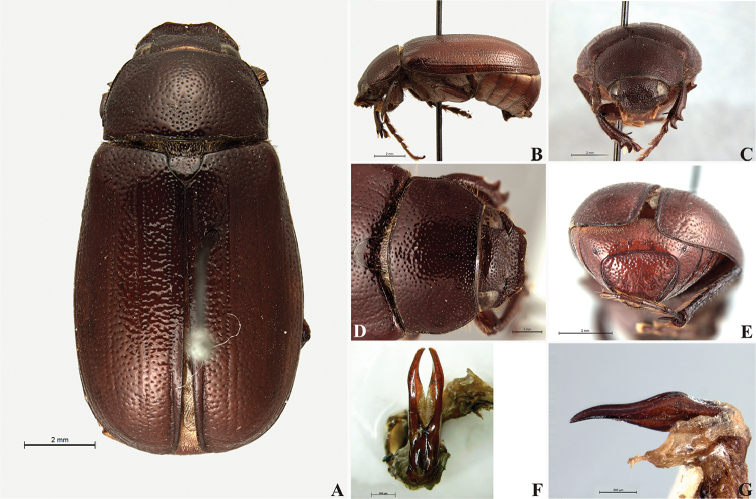
*Liogenys elegans* Nonfried. **A** Dorsal view **B** Lateral view **C** Frontal view **D** Clypeus and pronotum **E** Pygidium **F** Parameres, dorsal view **G** Parameres, lateral view.

Body, pronotum and elytra brownish to dark brown, very shiny; elongate; clypeal emargination shallow, rounded and very wide; outer sides of anterior teeth follow the lateral margin of clypeus; clypeal lateral margin straight; canthus exceeding the outer margin of the eye; pronotal, lateral margins barely convex; pronotal posterior corners sharp, almost right-angled; mesotibia sub-quadrate in cross section; pygidium wide, convex in males, pygidial midline sulcated in females; pygidial disc wrinkled, coarsely punctured; almost glabrous, bristled only on apex; parameres widened ventrally at the transverse midline and narrowed towards the fusiform apex; inner margins convergent (Fig. 63F).

##### Redescription.

Length: 12.7–13.5 mm; width: 6.8–7.1 mm. Brownish to dark brown. *Head*: distance between eyes nearly twice the width of one eye; frons equal in length than clypeus; clypeal emargination shallow, rounded and very wide, apparently truncated; outer sides of anterior teeth follow the lateral margin of clypeus; clypeal lateral margin straight; canthus exceeding the outer margin of the eye; distal maxillary palpomere, maximum width less than twice width of apex; fovea deep and elongate, extending past the transverse midline of the palpomere; labium transversely carinated, as wide as it is long; antenna 10-articulated, lamellae lighter in color and longer than flagellum. *Thorax*: anterior margin of pronotum slightly produced medially; maximum length of pronotum exceeding the length of tarsomeres I, II and III together; disc glabrous, punctures coarse and sparse; pronotal lateral margins barely convex; pronotal posterior corners sharp, almost right-angled, proepisternum with short bristles; mesepisternum scaly; sides of metasternum scaly and bristled, few long bristles on the anterior margin; distance between meso- and metacoxae up to twice longer than the metacoxa; scutellum rounded or sub-rounded, coarsely punctured. *Elytra*: very shiny, glabrous, dark reddish brown, darker at the base; elytra more than three times longer than the pronotum; elytral suture and elytron unicolored, distinctly elevated; all four elytral ridges barely noticeable. *Legs*: procoxa scaly on infra-carinal and outer surface; punctures visible at 12× magnification; three protibial teeth, middle and apical equal in size, distance between basal and middle teeth longer than between middle and apical; protibial inner apical spur present; mesofemural disc setose, with a row of long bristles on anterior and posterior margins; mesotibia sub-quadrate in cross section; disc finely sculptured, two mesotibial transverse carinae, the apical one complete; basal apophysis of metacoxa produced beyond the outer margin of trochanter; metatibia with posterior discontinuous longitudinal carina; metatibial apical spurs equal in length, length equal to the diameter of the tibial apex; inner margin of male metatibia carinated towards apex, apical inner surface setose; disc coarsely sculptured; two metatibial transverse carinae present posteriorly; basal metatarsomere shorter and wider than tarsomere II; in males protarsomere II long; pro- and mesotarsomeres I to IV enlarged, protarsomeres slightly wider the mesotarsomeres, less than twice as wide as metatarsi; claw bifid, symmetrical, superior tooth longer and narrower than the inferior; distance between teeth as long as the inferior tooth. *Abdomen*: ventrites with sparse short bristles on disc and sides; propygidium slightly visible, glabrous; pygidium convex, sub-trapezoidal, wide; pygidial width not exceeding distance between spiracles of propygidium, pygidial disc glabrous, shiny, bristled only on apex, wrinkled, coarsely punctured; pygidial apex in males sub-quadrate. *Parameres*: basal region as wide as the parameres together at its maximum width; parameral split at 2/3; inner margins convergent; parameres widened ventrally at the transverse midline and narrowed towards the fusiform apex (Fig. 63F). In lateral view concave; apex curved downwards partially (Fig. 63G).

##### Type-locality.

*Liogenys elegans*: BRAZIL; *Liogenys brasiliensis*: BRAZIL. Rio Grande do Sul (syn); *Liogenys forsteri*: ARGENTINA. Huerta Grande, Córdoba (syn.).

##### Geographical distribution.

**PARAGUAY (IT)**; BRAZIL (**PR**, **SC**, RS); **ARGENTINA (MI**, CO) .

##### Remarks.

*Liogenys elegans* is the sister lineage of a clade conformed by the species: *L. tibialis*, *L. punctaticollis*, *L. testaceipennis* and *L. spiniventris* (Cherman et al. 2016). *Liogenys elegans* shares with the species of the clade the body size elongate; the pronotal posterior corners sharp, almost right-angled; pygidial disc coarsely punctured and bristled only on apex; in females ventrites II to IV furnished with protuberances medially which are barely noticeable and visible only in lateral view. *Liogenys elegans* differs in the clypeal emargination being apparently truncated, shallow and wide; clypeus always straight laterally, pronotal convexity of lateral margins barely noticeable (like in *L. unicolor*); pygidium wide and male genitalia distinctive. The primary types of *L. elegans* (ZMHB) and *L. forsteri* (NHMB) were compared and we concluded than they are conspecific, so herein is designated *L. forsteri* a **junior subjective synonym** of *L. elegans*.

#### Liogenys fusca

Taxon classificationAnimaliaColeopteraMelolonthidae

Blanchard, 1851

Figs 64 , 92

Liogenys fuscus Blanchard, 1851: 168 (orig. desc.), Lacordaire 1856: 269 (sys.); Harold 1869a: 1140 (check.); Dalla Torre 1913: 318 (check.); Frey 1969: 47, 55 (key, red.).
Liogenys fusca : Blackwelder 1944: 227 (check.); Evans 2003: 209 (check.); Evans and Smith 2005: 174 (check.); Evans and Smith 2009: 178 (check.).
Liogenys argentinus Moser, 1918: 97 (orig. desc.); Frey 1969: 55 (syn.).
Liogenys argentina : Blackwelder 1944: 227 (check.).
Liogenys cuyabanus Moser, 1919: 12 (orig. desc.); Frey 1969: 55 (syn.).
Liogenys cuyabana : Blackwelder 1944: 227 (check.).

##### Type material.

*Liogenys fuscus* female syntype (MNHN): [white handwritten] “De l’emb./de l’Uruguay/jusqu’aux/missions”, [green handwritten] “Liogenys fuscus/Cat. Mus./Rives de l’Uruguay/ M. A. St Hilaire”, [red printed] “SYNTYPE”, [light green printed] “MUSÉUM PARIS”. This type is here designated the **lectotype** [white, outlined in red, printed], “LECTOTYPE/*Liogenys fuscus*/Blanchard, 1851/ des. M. A. Cherman 2012”. Female syntype (MNHN): [white handwritten] “6449/34”, [white handwritten] “1444”, [light green printed] “MUSÉUM PARIS/ [handwritten] Santa Cruz/D’Orbigny”. This type is here designated the **paralectotype**: [white, outlined in red, printed] “PARALECTOTYPE/ *Liogenys fuscus*/Blanchard, 1851/ des. M. A. Cherman 2012”. *Liogenys argentinus* male syntype ZMHB: [grey printed] “Argentinien/Santiago del Estero”, [white handwritten] “Liogenys/argentinus/Mos./Typen m# ”, [red printed] “Typus”, [red printed] “SYNTYPUS/Liogenys/argentinus Moser, 1918/labelled by MNHUB 2011”. Genitalia mounted. This type is here designated the **lectotype** [white, outlined in red, printed] “LECTOTYPE/*Liogenys argentinus*/Moser, 1918/ des. M. A. Cherman 2012”. Seven male and seven female syntypes (ZMHB): [grey printed] “Argentinien/ Santiago del Estero”, [white handwritten] “Liogenys/argentinus/Mos./ Typen”, [red printed] “Typus”, [red printed] “SYNTYPUS/Liogenys/argentinus Moser, 1918/ labelled by MNHUB 2011”. These fourteen syntypes are here designated as **paralectotypes**, each one with the label: [white, outlined in red, printed] “PARALECTOTYPE/*Liogenys argentinus*/Moser, 1918/ des. M. A. Cherman 2012”.

*Liogenys cuyabanus* male syntype (ZMHB): [white handwritten] “Cuyaba/Mato Grosso”, [white handwritten] “Liogenys/cuyabanus/Mos./Type m#”, [light red printed] “Typus”, [red printed] “SYNTYPUS/Liogenys/cuyabana Moser, 1919/labelled by MNHUB 2011”. Genitalia mounted. This type is here designated the **lectotype** [white, outlined in red, printed] “LECTOTYPE/*Liogenys cuyabanus*/Moser, 1919/ des. M. A. Cherman 2012”. Four female syntypes (ZMHB): [white handwritten] “Cuyaba/Mato Grosso”, [white handwritten] “Liogenys/cuyabanus/Mos./Typen f#”, [light red printed] “Typus”, [red printed] “SYNTYPUS/Liogenys/cuyabana Moser, 1919/labelled by MNHUB 2011”. These four syntypes are here designated as **paralectotypes**, each one with the label: “PARALECTOTYPE/*Liogenys cuyabanus*/Moser, 1919/ des. M. A. Cherman 2012”.

##### Non-type material.

PARAGUAY. “Estancia Postillón, Puerto max. A. Rio Paraguay”, without date, Louis des Arts Jr. Col., 1 ex. (ZMHB); PH: Puerto Pinasco, IX/1916, without collector, 2 ex. (AMNH); Campo León, X/1979, A. Martinez col., 1 ex. (CMNC); BQ: Filadelfia, X/1979, A. Martinez col., 2 ex. (CMNC). BOLIVIA. SC: Santa Cruz de la Sierra, without date, M. D’Orbigny col., 2 ex. (MNHN); Camiri, 9/XI/1946, Maldonado col., 2 ex. (CMNC); Comarapa, “9 km SW”, 17°58.926'S, 64°34.365'W, XII/2008, Edmonds and Vidaurre cols., 1 ex. (CEMT); San Ramon, “Laguna Brava”, 15/IX/1987, A. Castillo col., 1 ex. (AMNH); TA: Tatarenda, without date, 618 m, Erland Nordenskj col., 1 ex. (AMHB). BRAZIL. TO: Porto Nacional, XI/1953, R. Aires col., 1 ex. (DZUP); BA: Encruzilhada, 15°34'35"S, 40°56'51"W, 15/XII/2012, 850 m, Rafael and Grossi col., 2 ex. (INPA); MG: Arinos, 6/XI/1964, Exp. Dep. Zool. Col., 1 ex. (MZSP); Águas Vermelhas, “Faz. Faccino”,12/XII/2012, P. Grossi col., 1 ex. (EPGC); DF: Brasília, 15°44'10.30"S, 47°56'6.6"W, XII/2009, 1070 m, Carvalho col., 1 ex. (CEMT); Planaltina, “Embrapa Cerrados”, 15°36'16"S, 47°44'16"W, 18/XI/2009, C. Oliveira col., 1 ex. (CEMT); GO: Aragarças, X/1959, M. Alvarenga, 2 ex. (DZUP); MT: Barão de Melgaço, 21/V/2011, Sol col., 1 ex. (CEMT); Barra do Garças, 1990, Serrano col., 1 ex.; 15/X/1988, Capucho col., 1 ex.; 4/X/1988, Pereira col., 1ex. (CEMT); Boa Esperança 3/XI/1991, Oliveira col., 1 ex. (CEMT); Cáceres, 9/XI/1984, C. Elias col., 1 ex. (DZUP); Chapada dos Guimarães, 27-30/IX/2005, Marques col., 5 ex.; 5/X/2005, Marques col., 1 ex.; 30/V/2011, Sol col., 1 ex.; 27/V/2011, Klemp col., 1 ex.; “EE Buriti”, 6/X/1984, Dal Ponte col., 1 ex.; “Água Fria”, 8/X/1989, without collector, 1 ex.; (CEMT); Cuiabá, without date and collector, 8 ex.; 7/IX/1988, Loverde col. 1 ex.; 6/X/1988, Oliveira, Alves and Macieski cols., 4 ex.; 7/X/1988, Resende col., 1 ex.; 10/XI/1990, Aicio col., 3 ex.; 13/X/1991, Souza col., 1 ex.; 8/XI/1991, Aquino col., 1 ex.; 14/II/1992, Abido col., 1 ex.; 10/VIII/1992, Edenir col., 1 ex.; 8/IV/1993, Almindo Filho col., 1 ex.; 6/V/1993, Serrano col., 1 ex.; 20-25/VII/1993, Serrano col., 2 ex.; 17/VIII/1993, Serrano col., 1 ex.; 19/VIII/1993, Romio col., 1 ex.; 24/VIII/1993, Pinto col., 4 ex.; 8/XI/1993, Godoy col., 1 ex.; 21/XI/1993, Silva col., 4 ex.; 10/IV/1994, silveira col., 1 ex.; 12/III/1994, Ormond col., 1 ex.; 9/IX/1994, Montori col., 1 ex.; 1, 29/X/1994, Montori col. 2 ex.; 16-18/X/1994, Solange col., 2 ex.; 20/VI/2009, Silva col., 1 ex.; 11/IV/2010, Marcondes col., 1 ex.; “campus UFMT” 24/X/1988, Oliveira col., 1 ex.; 4/X/1990, Marques col., 1 ex.; 8/XI/1990, Alves Costa col., 1 ex.; 14/IX/1991, Souza col., 1 ex.; X/2008, Vaz-de-Mello col., 12 ex.; 13/IX/1988, Marques col., 1 ex.; 10/XI/1988, Leni col., 1 ex.; 13/IX/1988, Marques col. 1 ex; “Fazenda Birvana”, X-XI/1988, Serrano col., 2 ex.; “Duque de Caxias, 15/III/2011, Cardodo col., 2 ex.; “ Rodoviária”, 15/IV/2011, Vaz-de-Mello col., 14 ex.; “Santa Cruz”, 30/XI/2010, Kramer col., 1 ex.; “Res. Coxipó”, 12/IV/1989, Gianni col., 1 ex.; 22/XI/2010, F. Vaz-de-Mello col., 1 ex.; “Boa Esperança” X/2010, Vaz-de-Mello col., 1 ex.; 5/V/2010, Rabello col., 1ex.; “Jardim Vitória” 22/VIII/2009, Galvão col., 2 ex.; “Recanto do Sol” 23/X/2009, Dal Mas col., 1 ex.; “Cophamil” 27/VIII/1990, Regina col., 1 ex.; “Santa Amalia”, 22/V/2011, Klemp col., 1 ex.; “Verdão”,25/X/1989, Roseni col., 1 ex.; “Jardim das Américas” 11/VI/2010, Amado col., 1 ex.; Lucas do Rio Verde, 3/I/2012, Camera col., 2 ex. (CEMT); Nova Mutum, “Alto da Colina”, 20/VII/2010, Amhold col., 1 ex. (CEMT); Nossa Senhora do Livramento, 15/XI/1992, 2 ex.; 12/XII/1992, 1 ex. (CEMT); Pari, 6/X/1988, Auxiliadora col., 1 ex. (CEMT); Pocone, “SESC pantanal”, 10/IV/2009, Abreu col., 1 ex.; 14/V/2002, without collector, 2 ex.; 6/IX/2002, Myazaki col., 4 ex.; 7/IX/2002, without collector, 5 ex.; 12/X/2002; without collector, 1 ex.; “Fazenda Santa Maria”, 23/XII/2011, Semedo col., 1 ex.; “Fazenda Bom Jesus”, 24/XII/2010, Santos col., 1 ex.; “Transpantaneira, Porto Jofre”, 5/V/1984, Silva col., 1 ex. (CEMT); Porto Esperidião, XI/1984, Magno, Alvarenga cols., 3 ex. (MNRJ); Rondonópolis, 6/IX/1991, Viegas col., 1 ex. (CEMT); Rosario Oeste, XI/1976, A. Maller col., 2 ex. (MNRJ); Santo Antonio do Leverger, 16/XI/2008, Becker col., 1 ex. (CEMT); Tangará da serra, “Chacara Asa Branca”, 14°32'42"S, 58°40'28"W, XI/2011, Meurer col., 8 ex. (CEMT). Várzea Grande, 8/VI/2011, Uchaki col. 1 ex.; 27/X/1991, Bento col., 1 ex.; 5/XI/2008, Silva col., 1 ex. (CEMT); MS: Corumbá, “Nhecolandia”, 17/X/1953, C. R. Gonçalves col., 11 ex. (DZUP); 11 ex. (MNRJ); “Fazenda São Bento próxima a Porto Jofre”, 6/XII/2010, Tortato col., 1 ex. (CEMT); Salobra, 29/VIII/1940, Com IOC col., 1 ex.; XI/1941, Com IOC col., 2 ex. (CEIOC); Porto Murtinho, “Faz. Campo Florido”, 11/XII/2012, M Savaris and S Lampert col., 2 ex. (DZUP). Rio Verde [Rio Verde do Mato Grosso], XI/1963, 400 m, A. Maller, 2 ex (DZUP); “Zona da NOB Oswaldo Cruz Correntes” [Dois Irmãos do Burito], 17/X/1938, without collector, 4 ex. (CEIOC); SP: Jundiaí, 10/II/2012, Castro col., 1 ex.; 30/XII/2011, Castro col., 1 ex. (CEMT); Campinas, without date and collector, 1 ex. (ZMHB); RS: São Francisco de Paula, 29°25'22.4"S, 50°23'11.2"W, 10/XII/2009, P. Grossi col., 1 ex. (CEMT). ARGENTINA. SA: Aguaray, XII/1959, A. Martinez col., 1 ex. (MZSP); FO: Laguna Yema, 24°21'20,95"S, 61°18'55,23"W, 11/XII/2008, Ocampo, San Blas, Campon cols., 5 ex. (IADIZA); Rio Pilcomayo, 9/X/1906, Hermann J. col., 1 ex. (ZMHB); SE: without date, Wagner col., 4 ex. (MLPA); TU, IX/1900, C. Bruch col., 1 ex. (MACN); CH: Asustado, without date, Richter col., 1 ex. (MLPA), Resistencia, XII/1961, A. Martinez col., 2 ex. (CMNC); CR: Bella Vista, IV/1931, without collector, 1 ex. (MLPA); CO: “Córdoba, Casa Hernandez”, XI/1965, A. Martinez col., 2 ex. (CMNC); Rio Cuarto, without date, Bremer col., 1 ex. (ZMHB).

##### Diagnosis.

**Figure 64.**
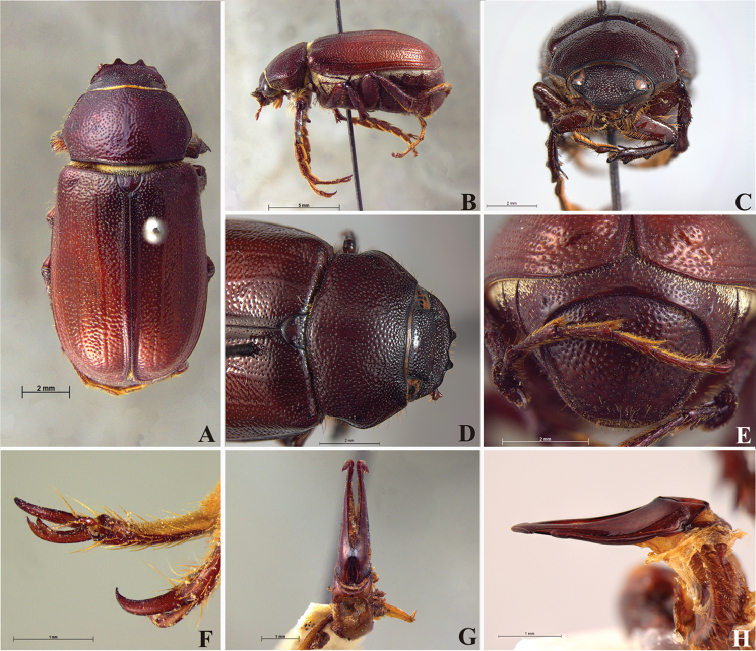
*Liogenys fusca* Blanchard. **A** Dorsal view **B** Lateral view **C** Frontal view **D** Clypeus and pronotum **E** Pygidium **F** Male protarsal claw **G** Parameres, dorsal view **H**. Parameres, lateral view.

Body, pronotum and elytra purplish brown or dark brown; elongate; distance between eyes slightly more than twice the width of one eye; frons slightly longer than clypeus; clypeal lateral margin convex, with a projection that varies from rounded to sharp; pronotal convexity at sides distinct (Fig. 64D); pronotal posterior corners sharp, obtuse-angled; prothorax scaly posteriorly (Fig. 64A); pro-, meso- and metasternum, pro- and metacoxae scaly (Figs 64B, E); male mesotibia sub-quadrate in cross section; inner margin of male metatibia abruptly sub-basally or medially produced towards apex; protarsi, male claws unequal, symmetrical in females; basal metatarsomere one-half the length of tarsomere II and wider; pygidium noticeable convex and wide, pygidial width exceeding distance between spiracles of propygidium; total length of parameres more than five times the length of their apex; inner margins slightly convergent; apex harpoon-like with lateral angle projecting straight downward (Fig. 64G). In lateral view parameres concave (Fig. 64G).

##### Redescription.

Length: 12.4–14.0 mm; width: 6.5–7.6 mm. Purplish brown. *Head*: distance between eyes slightly more than twice the width of one eye; frons slightly longer than clypeus; clypeal emargination rounded, shallow and narrow; outer sides of anterior teeth sub-parallel or parallel; clypeal lateral margin convex, with a projection that varies from rounded to sharp; distance between clypeal lateral projection and anterior margin of eye slightly longer than one eye; distance between clypeal lateral projection and anterior tooth longer than basal width of anterior tooth; obtuse angle between outer side of anterior teeth and clypeal lateral projection; canthus not exceeding the outer margin of the eye; distal maxillary palpomere, maximum width more than twice width of apex; fovea deep and oval, extending past the transverse midline of the palpomere; labium transversely carinated, as wide as it is long; antenna 10-articulated, lamellae lighter in color than flagellum and in males lamellae longer than the flagellum. *Thorax*: anterior margin of pronotum slightly produced medially; maximum length of pronotum exceeding the length of tarsomeres I, II and III together; disc glabrous, punctures fine and dense, smooth at posterior longitudinal midline; pronotal convexity at sides distinct; pronotal posterior corners sharp, obtuse-angled, in some cases almost right-angled; prothorax scaly posteriorly; proepisternum with long bristles and scales; mesepisternum scaly, as are the sides of metasternum, also with few long bristles on the anterior margin; distance between meso- and metacoxae up to twice longer than the metacoxa; scutellum ogival, finely punctured. *Elytra*: shiny, glabrous, uniform dark brown to purplish, elytra more than three times longer than the pronotum; elytral suture and elytron unicolored, distinctly elevated; pair of inner ridges more noticeable than the other three outer pairs. *Legs*: procoxa scaly on infra-carinal and outer surface; punctures visible at 12× magnification; three protibial teeth, middle and apical equal in size, distance between basal and middle teeth slightly longer than between middle and apical; protibial inner apical spur present; mesofemural disc setose, with a row of long bristles on anterior and posterior margins; mesotibia sub-quadrate in cross section in males, cylindrical in females; disc coarsely sculptured, two mesotibial transverse carinae, the apical one complete; basal apophysis of metacoxa produced beyond the outer margin of trochanter; metatibial apical spurs of different lengths, the longest one shorter than the diameter of the tibial apex; inner margin of male metatibia carinated and abruptly sub-basally produced towards apex, apical inner surface setose; metatibial disc finely sculptured in males, coarsely sculptured in females; two metatibial transverse carinae present posteriorly; basal metatarsomere one-half the length of tarsomere II and wider, in males protarsomere II long; pro- and mesotarsomeres I to IV enlarged, protarsomeres slightly wider than the mesotarsomeres and more than twice as wide as metatarsi; protarsal claws asymmetrical in males, symmetrically bifid in females; superior tooth longer and as wide as the inferior; distance between teeth as long as the inferior tooth. *Abdomen*: band of abundant scales visible at the lowest magnification beneath the outer margin of elytra; ventrites bristled on disc; propygidium visible, glabrous; pygidium noticeable convex, sub-quadrate, wide, pygidial width exceeding distance between spiracles of propygidium; pygidial disc bristled only on apex, coarsely punctured, pygidial apex quadrate. *Parameres*: width of basal region equal to the parameres together at its transverse midline, parameral split at 2/3; total length of parameres more than five times the length of their apex; inner margins slightly convergent; apex harpoon-like with lateral angle projecting straight downward (Fig. 64G). In lateral view parameres concave (Fig. 64H).

##### Type-locality.

*Liogenys fuscus*: Rives de l’Uruguay [today Rio Grande do Sul, Brazil]; *Liogenys argentinus*: ARGENTINA. Santiago del Estero (syn.); *Liogenys cuyabanus*: BRAZIL. Cuiabá, Mato Grosso (syn.).

##### Geographical distribution.

BRAZIL (**TO**, **BA**, **MG**, DF, GO, MT, MS, **SP**, RS); PARAGUAY (BQ, PH); **BOLIVIA (Santa Cruz**, **Tarija)**; ARGENTINA **(SA**, **TU**, **FO**, **CH**, SE, **CR**, **CO)**.

##### Remarks.

*Liogenys fusca* resembles *L. bidentata* (Fig. 57) and *L. pallidicornis* (Fig. 67). Cherman et al. (2016) support that those species are closely related. *Liogenys fusca* differs from the other two in the quadridentate clypeus, being more evident in males; clypeal emargination rounded; elytral suture distinctly elevated; elytral pair of inner ridges more noticeable than the outers; pygidial disc swollen; basal metatarsomere one-half the length of tarsomere II and wider; inner margin of male metatibia abruptly sub-basally produced towards apex, claws of protarsi asymmetrical, one bifid and one simple (Fig. 64F) and parameres concave.

#### Liogenys laminiceps

Taxon classificationAnimaliaColeopteraMelolonthidae

Moser, 1919

Figs 65 , 91

Liogenys laminiceps Moser, 1919: 16 (orig. desc.); Blackwelder 1944: 227 (check.); Frey 1969: 38 (key); Evans 2003: 210 (check.); Evans and Smith 2005: 174 (check.); Evans and Smith 2009: 178 (check.).

##### Type material.

*Liogenys laminiceps* male holotype (ZMHB): [white printed] “Brasilia/ [handwritten] Sao Paulo”, [white handwritten] “Liogenys/laminiceps/Mos/Typen”, [light red printed] “Typus”, [white printed] “Liogenys/laminiceps/Mos.”, [red printed] “HOLOTYPUS/Liogenys/laminiceps Moser, 1919/labelled by MNHUB 2013”. Genitalia mounted.

##### Diagnosis.

**Figure 65.**
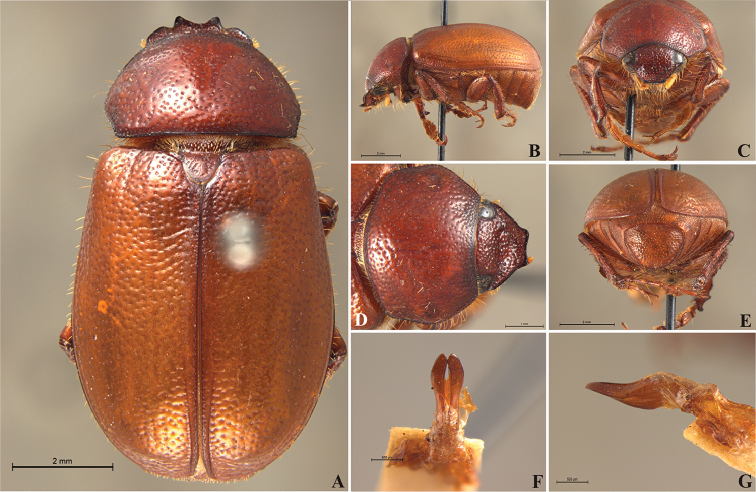
*Liogenys laminiceps* Moser. **A** Dorsal view **B** Lateral view **C** Frontal view **D** Clypeus and pronotum **E** Pygidium **F** Parameres, dorsal view **G** Parameres, lateral view.

Body and elytra reddish brown; elongate, widest at posterior third; pronotum darker; clypeal emargination shallow, rounded and wide; outer sides of anterior teeth parallel; clypeal lateral margin convex, with a rounded projection, obtuse angle between outer side of anterior teeth and clypeal lateral projection; antenna 9-articulated; pronotal disc punctures very sparse; pronotal posterior corners sub-angled, obtuse; mesotibia quadrate in cross section; metafemur with thick and erect bristles on posterior margin; pygidium convex; in males, parameres widened from the midline towards the apex, maximum width subapically; apex rounded; inner margins convergent (Fig. 65F).

##### Redescription.

Length: 9.8 mm; width: 5.5 mm. Reddish brown. *Head*: distance between eyes nearly twice the width of one eye; frons shorter than clypeus; clypeus bristled anteriorly; clypeal emargination rounded, shallow and wide; outer sides of anterior teeth parallel; outer margin of anterior teeth shorter than the eye; clypeal lateral margin convex, with a rounded projection; distance between this projection and anterior tooth shorter than basal width of anterior tooth, distance between clypeal lateral projection and anterior margin of eye shorter than one eye; obtuse angle between outer side of anterior teeth and clypeal lateral projection; canthus not exceeding the outer margin of the eye; distal maxillary palpomere, maximum width slightly wider than the apex; fovea deep and elongate, extending to or past the transverse midline of the palpomere; labium transversely carinated, as wide as it is long; antenna 9-articulated, lamellae lighter in color than flagellum and equal in length. *Thorax*: anterior margin of pronotum straight, flanged throughout; maximum length of pronotum exceeding the length of tarsomeres I, II and III together; disc glabrous, punctures very sparse and coarse; pronotal posterior corners sub-angled, obtuse; proepisternum with long bristles; mesepisternum scaly; sides of metasternum scaly and bristled, few long bristles on the anterior margin; distance between meso- and metacoxae up to twice longer than the metacoxa; scutellum rounded, coarsely punctured. *Elytra*: shiny, glabrous, uniform reddish brown; elytra more than three times longer than the pronotum; elytral suture and elytron unicolored, distinctly elevated; all four elytral ridges barely noticeable. *Legs*: procoxa scaly on infra-carinal and outer surface, punctures visible at 12× magnification; three protibial teeth, middle and apical equal in size, the three teeth equally spaced; protibial inner apical spur present; mesofemural disc setose, with a row of long bristles on anterior and posterior margins; mesotibia quadrate in cross section, disc finely sculptured; two mesotibial transverse carinae, the apical one incomplete; basal apophysis of metacoxa produced beyond the outer margin of trochanter; metafemur with thick and erect bristles on posterior margin; inner margin of metatibia carinated towards apex; inner surface setose; disc finely sculptured; metatibial transverse carina present posteriorly and posterior discontinuous longitudinal carina; metatibial apical spurs of different lengths, the longest equal in length to the diameter of the tibial apex; basal metatarsomere wider than tarsomere II and equal in length; protarsomere II short and wide; pro- and mesotarsomeres I to IV enlarged, protarsomeres slightly wider than the mesotarsomeres and more than twice as wide as metatarsi; claw bifid, symmetrical, superior tooth longer and as wide as the inferior; distance between teeth as long as the inferior tooth. *Abdomen*: band of scales visible at the lowest magnification beneath the outer margin of elytra; ventrites bristled on disc and sides; propygidium visible, bristled and scaly; pygidium convex, sub-trapezoidal, wide, pygidial width not exceeding distance between spiracles of propygidium, pygidial disc bristled throughout; pygidial apex rounded. *Parameres*: basal region with parameral split at 2/3; parameres widened from the midline towards the apex, maximum width subapically (Fig. 65F); apex rounded; inner margins convergent. In lateral view parameres concave (Fig. 65G).

##### Type-locality.

BRAZIL. São Paulo.

##### Geographical distribution.

BRAZIL (SP).

##### Remarks.

*Liogenys laminiceps* is one of the few species with antenna 9-articulated, together with *L. sinuaticeps* (Fig. 72) and *L. flavida*, a southern Argentinian species. The last two species are easily differentiated from *L. laminiceps* because they are yellowish and with sides of body being almost parallel. The female of *L. laminiceps* remains unknown.

#### Liogenys moseri

Taxon classificationAnimaliaColeopteraMelolonthidae

Frey, 1969

Figs 66 , 93

Liogenys moseri Frey, 1969: 60, 49 (orig. desc., key); Evans 2003: 211 (check.); Evans and Smith 2005: 175 (check.); Evans and Smith 2009: 179 (check.).

##### Type material.

*Liogenys moseri* male syntype (ZMHB): [white printed] “Rio Jan”, [white printed] “Liogenys/laminiceps/Mos.”, [light red printed] “Typus”, [white handwritten] “Type/ [printed] Liogenys/ [handwritten] moseri n. sp./[printed] det G. Frey, 1968”, [red printed] “SYNTYPUS/Liogenys/moseri Frey, 1969/labelled by MNHUB 2014”. This type is here designated the **lectotype** [white, outlined in red, printed] “LECTOTYPE/*Liogenys moseri*/Frey, 1969/des. M. A. Cherman 2014”. Genitalia mounted. Male syntype (NHMB): [white printed] “Rio Jan”, [white handwritten] “Liogenys/sjoestedti/Mos/Typen.”, [red printed] “PARATYPE”, [white printed] Liogenys/ [handwritten] moseri/n. sp./[printed] det G. Frey, 1968”, [white handwritten] “Wagner”, [white printed] “Hieke/Berlin”. This type is here designated the **paralectotype** [white, outlined in red, printed] “PARALECTOTYPE/*Liogenys moseri*/Frey, 1969/des. M. A. Cherman 2014”.

##### Non-type material.

BRAZIL. Without locality and date, 1 ex. (NHRS); MT: Pocone: Margen baia do Burro, 17°50.73'S, 57°24.17'W, 15/XI/2011, J. L. da Silva col., 1 ex.; without date, 1 ex.; Sede do parque [Parque Nacional do Pantanal Matogrossense], 17°50.73'S, 57°24.17'W, 15/XI/2011, J. L. da Silva col., 1 ex. (CEMT).

##### Diagnosis.

Body and elytra testaceous, elongate, sides almost parallel; pronotum reddish brown; clypeal emargination shallow, rounded and wide; outer sides of anterior teeth parallel; clypeal lateral margin with a sharp tooth-like projection, forming a deep right angle between this projection and the anterior teeth; pronotal posterior corners apparently rounded, weakly obtuse; mesepisternum, sides of metasternum, metacoxae and ventrites scaly; mesotibia quadrate in cross section; male metafemur medially produced on posterior margin; pygidium flat; sub-quadrate; pygidial disc with abundant erect bristles throughout; punctures reticulated; parameres widened on apex, apex rounded; inner margins convergent.

##### Redescription.

**Figure 66.**
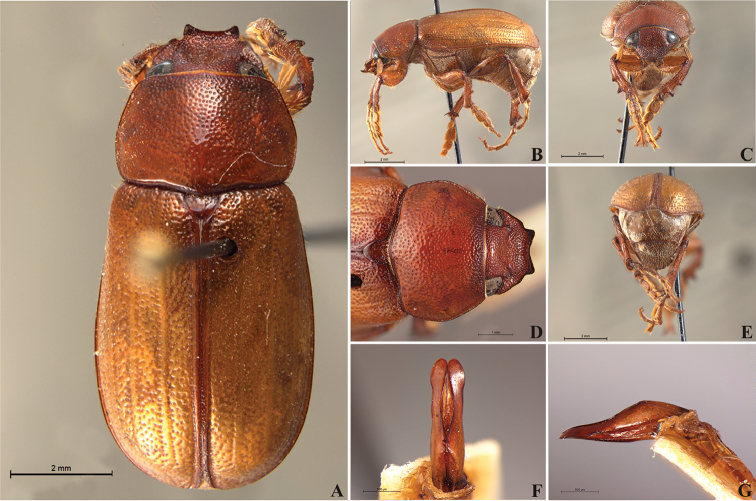
*Liogenys moseri* Frey. **A** Dorsal view **B** Lateral view **C** Frontal view **D** Clypeus and pronotum **E** Pygidium **F** Parameres, dorsal view **G** Parameres, lateral view.

Length: 9.8–10.8 mm; width: 4.6–5.0 mm. Testaceous. *Head*: distance between eyes nearly twice the width of one eye; frons shorter than clypeus; clypeal emargination rounded, shallow and wide; outer sides of anterior teeth parallel; outer margin of anterior teeth as long as the eye; clypeal lateral margin convex, with a sharp tooth-like projection, distance between clypeal lateral projection and anterior margin of eye longer than one eye, distance between tooth-like projection and anterior tooth shorter than basal width of anterior tooth; right angle between outer side of anterior teeth and clypeal lateral projection; canthus exceeding the outer margin of the eye; distal maxillary palpomere, maximum width more than twice width of apex, fovea deep; not reaching the transverse midline of the palpomere; labium transversely carinated, as wide as it is long; antenna 10-articulated, lamellae and flagellum unicolored and equal in length. *Thorax*: anterior margin of pronotum slightly produced medially; maximum length of pronotum exceeding the length of tarsomeres I, II and III together; disc glabrous, punctures coarse and dense; pronotal posterior corners apparently rounded, weakly obtuse; proepisternum with short bristles; mesepisternum scaly, as are the sides of metasternum, also with few long bristles on the anterior margin; distance between meso- and metacoxae up to twice longer than the metacoxa; scutellum ogival, coarsely punctured at the base. *Elytra*: shiny, glabrous, uniform testaceous, lighter in color than the pronotum; elytra more than three times longer than the pronotum; elytral suture slightly darker than elytron and distinctly elevated; all four elytral ridges barely noticeable. *Legs*: procoxa scaly on infra-carinal and outer surface; three protibial teeth, middle and apical equal in size, the three teeth equally spaced; protibial inner apical spur present; mesofemural disc setose; mesotibia quadrate in cross section; disc coarsely sculptured, two mesotibial transverse carinae, the apical one incomplete; basal apophysis of metacoxa produced beyond the outer margin of trochanter; metafemur medially produced on posterior margin; metatibial apical spurs of different lengths, the longest one shorter than the diameter of the tibial apex; inner margin of metatibia carinated towards apex, apical inner surface setose; metatibial disc finely sculptured; two metatibial transverse carinae present posteriorly; basal metatarsomere and tarsomere II equal in size, in males protarsomere II short and wide; in males pro- and mesotarsomeres I to IV enlarged and more than twice as wide as metatarsi; claw bifid, symmetrical, superior tooth longer and as wide as the inferior; distance between teeth as long as the inferior tooth. *Abdomen*: band of scales visible at the lowest magnification beneath the outer margin of elytra; ventrites scaly on disc; propygidium visible, bristled; pygidium flat, sub-quadrate, wide, pygidial width not exceeding distance between spiracles of propygidium; pygidial disc with abundant erect bristles throughout, punctures reticulated; pygidial apex rounded. *Parameres*: width of basal region equal to the parameres together at its maximum width, parameral split at 2/3; inner margins convergent; apex widened, rounded (Fig. 66F). In lateral view parameres slightly concave (Fig. 66G).

##### Type-locality.

BRAZIL. Rio de Janeiro.

##### Geographical distribution.

BRAZIL (RJ, **MT**).

##### Remarks.

*Liogenys moseri* resembles *L. obesina* Frey, 1969 —a northern Argentinian species— mainly in the shape of the parameres, but they differ in the body shape, being the sides more parallel in *L. moseri*; clypeal lateral margins produced; thorax and abdomen scaly ventrally; pygidial apex slightly narrower and bristles on disc thicker. The type-locality of *L. moseri* (Rio de Janeiro) is dubious, as the amount of localities recorded among the non-type material are from Mato Grosso state, which is very far from Rio de Janeiro. The type material was collected by Wagner during the end of 19^th^ century and beginning of the 20^th^, mainly in Rio Salado region (Santiago del Estero, Argentina). Probably, the original type-locality written on the label was “Rio Sal” instead of “Rio Jan”, misspelled by the person who wrote the definitive label. Female of *L. moseri* remains unknown.

#### Liogenys pallidicornis

Taxon classificationAnimaliaColeopteraMelolonthidae

Blanchard, 1851

Figs 67 , 89

Liogenys pallidicornis Blanchard, 1851: 167 (orig. desc.); Lacordaire 1856: 269 (sys.); Harold 1869a: 1140 (check.); Dalla Torre 1913: 318 (check.); Blackwelder 1944: 227; Evans 2003: 212 (check.); Evans and Smith 2005: 176 (check.); Evans and Smith 2009: 180 (check.)

##### Type material.

*Liogenys pallidicornis* male syntype (MNHN): [white handwritten] “Capit^e^/des Mines”, [green handwritten] “Liogenys pallidicornis./Cat. Mus./Brésil/M. A. St. Hilaire”, [light green printed] “MUSEUM PARIS. [handwritten] Cap. des/Mines”, [red printed] “SYNTYPE”. Genitalia mounted. This type is here designated the **lectotype** [white, outlined in red, printed] “LECTOTYPE/*Liogenys pallidicornis*/Blanchard 1851/des. M. A. Cherman 2014”. Male syntype (MNHN): [white handwritten] “Capit^e^/des Mines”, [green handwritten] “Liogenys pallidicornis./Cat. Mus./Brésil/M. A. St. Hilaire”, [light green printed] “MUSEUM PARIS. [handwritten] Cap. des/Mines”, [red printed] “SYNTYPE”. This type is here designated the **paralectotype**: [white, outlined in red, printed] “PARALECTOTYPE/*Liogenys pallidicornis*/Blanchard 1851/des. M. A. Cherman 2014”.

##### Non-type material.

BRAZIL. Without locality, date and collector, 4 ex. (ZMHB); CE: without date and collector, 2 ex. (ZMHB); Ubajara, “P.N. Portão Neblina”, 3°50'35.5"S, 40°54'1"W, 16/II/2013, 850 m, Vaz-de-Mello and Grossi col., 1 ex. (CEMT); “Nordeste”, without date and collector, 2 ex. (CEMT); RN: Jardim de Angicos, I/1952, M. Alvarenga, 1 ex. (DZUP); Natal, XII/1956, A.F. Magalhães col., 1 ex. (DZUP); 2 ex. (MNRJ); Mossoró, I/1952, M. Alvarenga col., 4 ex. (DZUP); SE: Caninde do São Francisco, “Faz. Miramar”, 11/III/2000, L. Iannuzzi col., 1 ex. (CEMT); BA: Jacobina, XII/1941, Mangabeira col., 2 ex. (CMNC). ARGENTINA. Chaco austral, without date, C. Bruch col., 1 ex. (MLPA).

##### Diagnosis.

**Figure 67.**
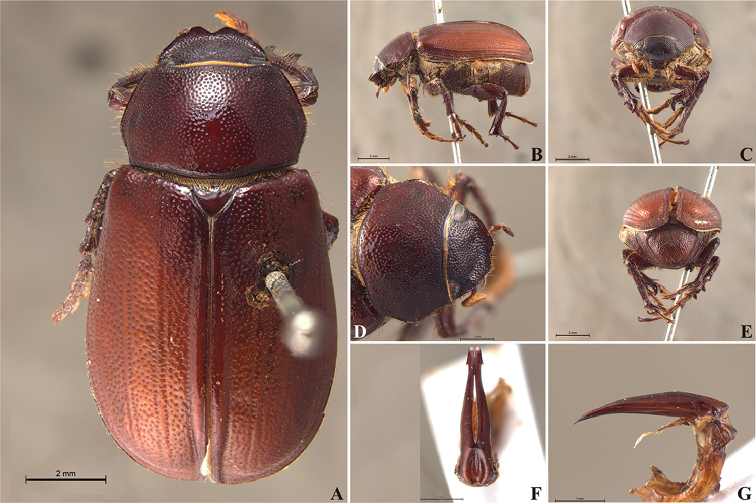
*Liogenys pallidicornis* Blanchard. **A** Dorsal view **B** Lateral view **C** Frontal view **D** Clypeus and pronotum **E** Pygidium **F** Parameres, dorsal view **G** Parameres, lateral view.

Body, pronotum and elytra purplish brown or dark brown; elongate; distance between eyes more than twice the width of one eye; frons longer than clypeus; clypeal emargination sub-angled, shallow and narrow; clypeal lateral margin convex; pronotal posterior corners sharp, obtuse-angled; prothorax scaly posteriorly; pro-, meso- and metasternum, pro- and metacoxae scaly abundantly; male mesotibia sub-quadrate in cross section; protarsal claws symmetrical; basal metatarsomere shorter than tarsomere II and slightly wider; pygidium flat or convex and wide, pygidial width exceeding distance between spiracles of propygidium; total length of parameres more than five times the length of their apex, narrowed subapically; inner margins convergent; apex harpoon-like with lateral angle curved projecting almost perpendicular to parameres (Fig. 67F).

##### Redescription.

Length: 11.0–13.0 mm; width: 6.1–7.2 mm. Purplish brown. *Head*: distance between eyes more than twice the width of one eye; frons longer than clypeus; clypeal emargination sub-angled, shallow and narrow; outer sides of anterior teeth follow the lateral margin of clypeus; canthus not exceeding the outer margin of the eye; distal maxillary palpomere, maximum width twice the width of apex; fovea shallow and oval, extending past the transverse midline of the palpomere; labium transversely carinated, as wide as it is long; antenna 10-articulated, lamellae lighter in color than flagellum and equal in length. *Thorax*: anterior margin of pronotum straight, flanged throughout; maximum length of pronotum exceeding the length of tarsomeres I, II and III together; pronotal disc glabrous, punctures dense and fine, smooth at the posterior longitudinal midline; pronotal posterior corners sharp, obtuse-angled; prothorax scaly posteriorly, abundant scales; proepisternum with long bristles; mesepisternum scaly, as are the sides of metasternum, also with few long bristles on the anterior margin; distance between meso- and metacoxae up to twice longer than the metacoxa; scutellum triangular, smooth. *Elytra*: shiny, glabrous, uniform dark brown to purplish; elytra more than three times longer than the pronotum; elytral suture and elytron unicolored and elevated; four elytral ridges barely noticeable. *Legs*: procoxa scaly on infra-carinal and outer surface; punctures visible at 12× magnification; three protibial teeth, middle and apical equal in size, in males the three teeth equally spaced, in females distance between basal and middle teeth longer than between middle and apical; protibial inner apical spur present; mesofemural disc glabrous, with a row of long bristles on the anterior margin; mesotibia sub-quadrate in cross section in males, cylindrical in females, disc finely sculptured, two mesotibial transverse carinae, the apical one complete; basal apophysis of metacoxa produced beyond the outer margin of trochanter; metatibial apical spurs of different lengths, the longest equal in length to the diameter of the tibial apex, inner margin of male metatibia carinated towards apex, apical inner surface setose; metatibial disc finely sculptured in males, coarsely in females; two metatibial transverse carinae present posteriorly; basal metatarsomere smaller and slightly wider than tarsomere II, in males protarsomere II long; pro- and mesotarsomeres I to IV enlarged, protarsomeres slightly wider than the mesotarsomeres and more than twice as wide as metatarsi; claw bifid, symmetrical, superior and inferior tooth equal in length and wider; distance between teeth shorter than the inferior tooth. *Abdomen*: band of abundant scales visible at the lowest magnification beneath the outer margin of elytra; ventrites bristled on disc and sides, on sides also scaly; propygidium visible, glabrous; pygidium flat or convex, sub-quadrate, wide; pygidial width exceeding distance between spiracles of propygidium; pygidial disc bristled only on apex, with long bristles; finely punctured; pygidial apex quadrate or sub-quadrate. *Parameres*: width of basal region equal to the parameres together at its maximum width, parameral split at 2/3; total length of parameres more than five times the length of their apex; inner margins convergent; narrowed sub-apically; apex harpoon-like with lateral angle curved projecting almost perpendicular to parameres (Fig. 67F). In lateral view parameres convex (Fig. 67G).

##### Type-locality.

BRAZIL. “Capit.e des Mines” [today Minas Gerais state].

##### Geographical distribution.

BRAZIL (**CE**, **RN**, **SE**, MG, **BA**); **ARGENTINA (“Chaco Austral”)**.

##### Remarks.

*Liogenys pallidicornis* resembles *L. fusca* (Fig. 64) and *L. bidentata* (Fig. 57) and differs from them in the shape of the clypeus being not produced laterally; scutellum smooth; and parameres being narrowed sub-apically, the shape of the apex is distinctive and parameres convex in lateral view. *Liogenys pallidicornis* is the sister lineage of *L. bidentata* (Cherman et al. 2016) and differs in the fovea of the distal maxillary palpomere being shallower and narrower, metatibial disc finely sculptured; metatarsomere I slightly wider than tarsomere II and pygidium convex.

#### Liogenys parva

Taxon classificationAnimaliaColeopteraMelolonthidae

Blanchard, 1851

Figs 68 , 89

Liogenys parvus Blanchard, 1851: 168 (orig. desc.); Harold 1869a:1140 (check.); Dalla Torre 1913: 318 (check.); Frey 1969: 61 [not Blanchard 1851] (red.).
Liogenys parva : Blackwelder 1944: 228 (check.); Evans 2003: 212 (check.); Evans and Smith 2005: 177 (check.); Evans and Smith 2009: 181 (check.).

##### Type material.

*Liogenys parvus* male syntype (MNHN): [white handwritten] “Capit.^e^/des mines [today Minas Gerais]”, [light green printed] “MUSEUM PARIS/ [handwritten] Caple des/Mines”, [green handwritten] “L. parvus/ Cat Mus/ Montevideo/ M. A. St. Hilaire”. Male genitalia mounted. This type is here designated as **lectotype**, bearing: [white, outlined in red, printed] “LECTOTYPE/*Liogenys parva*/Blanchard, 1851/des. M. A. Cherman 2016”.

##### Diagnosis.

**Figure 68.**
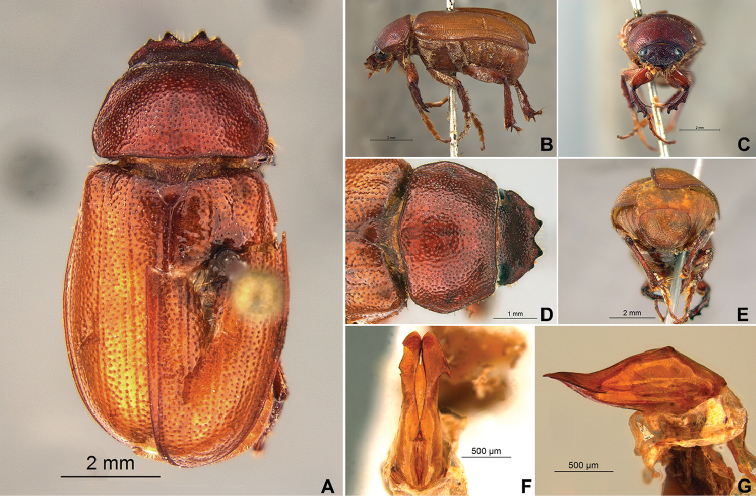
*Liogenys parva* Blanchard. **A** Dorsal view **B** Lateral view **C** Frontal view **D** Clypeus and pronotum **E** Pygidium **F** Parameres, dorsal view **G** Parameres, lateral view.

Body yellowish brown; elongate; elytra testaceous, pronotum darker; clypeus quadridentate due to the tooth-like projection laterally; clypeal emargination sub-angled and narrow; outer sides of anterior teeth parallel; distance between clypeal lateral and anterior tooth equal to basal width of anterior tooth, distance between clypeal lateral tooth and anterior margin of eye longer than one eye, right angle between clypeal lateral and anterior tooth; canthus exceeding the outer margin of the eye; mesotibia cylindrical in cross section; metacoxa punctured and bristled; pygidium convex, apex rounded; pygidial width exceeding distance between spiracles of propygidium; pygidial disc bristled on apex; male genitalia, total length of parameres less than three times the length of their apex; inner margins convergent; apex harpoon-like with lateral angle projecting straight downward (Fig. 68F).

##### Redescription.

Length: 8.0–9.0 mm; width: 4.4 mm. Yellowish brown. *Head*: distance between eyes nearly twice the width of one eye; frons length equal to clypeus; clypeal emargination deep, rounded and narrow; outer sides of anterior teeth parallel; outer margin of anterior teeth shorter than the eye; clypeus convex laterally and strongly produced forming a tooth-like projection; distance between lateral and anterior tooth equal to basal width of anterior tooth, distance between lateral tooth and anterior margin of eye longer than one eye, right angle between anterior and lateral teeth; canthus exceeding the outer margin of the eye; distal maxillary palpomere, maximum width twice width of apex; fovea deep, extending past the transverse midline of the palpomere; labium transversely carinated, as wide as it is long; antenna 10-articulated, lamellae lighter in color than flagellum and equal in length. *Thorax*: pronotal anterior margin of pronotum straight and depressed throughout (Fig. 68D); maximum length of pronotum exceeding the length of tarsomeres I, II and III together; disc glabrous, punctures sparse and coarse; pronotal posterior corners sub-angled, obtuse; proepisternum with long bristles; mesepisternum with few scales; sides of metasternum with few long bristles; distance between meso- and metacoxae twice longer than the metacoxa; scutellum sub-rounded, scarce punctures at the base. *Elytra*: shiny, glabrous, uniform yellowish to testaceous, lighter in color than the pronotum; elytra more than three times longer than the pronotum; elytral suture slightly darker than elytron and slightly elevated; all four elytral ridges barely noticeable. *Legs*: procoxa scaly and bristled, long bristles on infra-carinal surface and short bristles on outer surface; three protibial teeth, middle and apical equal in size, distance between basal and middle teeth longer than between middle and apical; protibial inner apical spur present; with a row of long bristles on the anterior and posterior margins of mesofemur, mesotibia cylindrical in cross section, disc finely sculptured, two mesotibial transverse carinae, the apical one incomplete; metacoxa coarsely punctured and scarcely bristled, basal apophysis of metacoxa produced beyond the outer margin of trochanter; inner margin of metatibia carinated towards apex, apical inner surface setose, disc finely sculptured; metatibial transverse carina present posteriorly and posterior discontinuous longitudinal carina; protarsomere II long; pro- and mesotarsomeres I to IV enlarged, protarsomeres slightly wider than the mesotarsomeres; claw bifid, symmetrical, superior teeth longer and narrower than the inferior; distance between teeth shorter than the inferior tooth. *Abdomen*: band of scales beneath the outer margin of elytra; disc of ventrites and propygidium bristled; pygidium convex, sub-trapezoidal, wide; pygidial width exceeding distance between spiracles of propygidium; pygidial disc glabrous, few long bristles on apex, coarsely punctured; pygidial apex rounded. *Parameres*: basal region slightly wider than the parameres together at its transverse midline; parameral split at 2/3; total length of parameres less than three times the length of their apex; inner margins convergent; apex harpoon-like with lateral angle projecting straight downward (Fig. 68F). In lateral view parameres straight not coplanar with basal region, concave apically (Fig. 68G).

##### Type-locality.

BRAZIL. “Capit.^e^ des Mines” [Minas Gerais state].

##### Geographical distribution.

**BRAZIL (MG)**.

##### Remarks.

The type-locality mentioned by Blanchard (1851) in the original description is “Montevideo”, as well as the green label added by the MNHN. This seems to be a mistake, because the original label of Saint Hilaire says “Capitanie des Mines”, corresponding to Minas Gerais, Brazil. As the lectotype studied matches with the original description, we concluded that it is in fact describing *L. parva*, and “Capitanie des Mines” [Minas Gerais] should be the right type-locality. As we did not find non-type material from Uruguay corresponding to *L. parva*, this distribution record is considered as incorrect. The female remains unknown.

Frey (1969) redescribed *L. parva* Blanchard but some differences were found comparing that redescription with the Blanchard's, type, as the pygidium smooth at 16x magnification and the shape of parameres drawn by Frey. Also, Frey mentioned “Argentinien, Pilcomayo, Formosa” as the type-locality and according to him, the types were deposited in Smithsonian Institute Washington (USNM) and in NHMB, different from *L. parva* Blanchard's, type. We did not find any *L. parva* type in both USNM and NHMB museums, though in NHMB there was found only one non-type specimen labeled by Frey as *L. parva*, with labels from USNM and Frey's, collection, labeled from Salta, Argentina (see *Liogenys freyi* Cherman, sp. n. remarks). After studying the *L. parva* Blanchard lectotype (MNHN), plus Frey's, (1969) redescription and the non-type specimen found in NHMB, we concluded that the *L. parva* redescribed by Frey (1969) does not correspond to *L. parva* Blanchard.

#### Liogenys punctaticollis

Taxon classificationAnimaliaColeopteraMelolonthidae

(Blanchard, 1851)

Figs 69 , 90

Hilarianus punctaticollis Blanchard, 1851: 169 (orig. desc.); Lacordaire 1856: 270 (sys.); Harold 1869a: 1141 (check.); Dalla Torre 1913: 319 (check.); Blackwelder 1944: 228 (check.).
Liogenys palmata Burmeister, 1855: 13 (orig. desc.); Blackwelder 1944: 227 (check.).
Liogenys palmatus : Harold 1869a: 1140 (check.); Dalla Torre 1913: 318 (check.); Frey 1969: 40, 56 (key, red.).
Liogenys punctaticollis : Frey 1974: 331 (n. comb.; senior syn. of L. palmata); Evans 2003: 213 (check.); Evans and Smith 2005: 177 (check.); Evans and Smith 2009: 181 (check.).
Hilarianus anguliceps Blanchard, 1851: 169 (orig. desc.); Lacordaire 1856: 270 (sys.); Harold 1869a: 1141 (check.); Dalla Torre 1913: 319 (check.); Blackwelder 1944: 228 (check.); Evans 2003: 273 (check.); Evans and Smith 2005: 230 (check.); Evans and Smith 2009: 308 (check.); Cherman et al. 2016: 23 (junior syn. of L. punctaticollis).

##### Type material.

*Hilarianus punctaticollis* male holotype (MNHN): [handwritten] “Brésil”, [light green printed] “MUSÉUM PÁRIS”, [red printed] “SYNTYPE”, [green handwritten] “H. punctaticollis/ Cat Mus/ Brésil/ M. de Castelnau”. Genitalia mounted.

*Liogenys palmata* male lectotype (MHLU): [handwritten] “palmata/ Germ./ Bras Int.”, [white handwritten] “Type/Liogenys/palmatus/burm/[printed] det. G. Frey 1967/68”. Genitalia mounted. Male paralectotype (MHLU): “P. Type/Liogenys/ palmatus/ burm/ [printed] det. G. Frey 1967/68”, [green printed] “Nov./Frib.”, [white printed] “Prof. Hüsind/Halle” Genitalia mounted. Female paralectotype (MHLU): “P. Type/Liogenys/palmatus/burm/[printed] det. G. Frey 1967/68”, [white printed] “Prof. Hüsind/Halle”. Those three types were labeled by Frey in 1967/68, as Type and P. Types and that is why they are here considered as lectotype and paralectotypes.

*Hilarianus anguliceps* female holotype (MNHN): [white handwritten] “71/44”, [light green printed] “MUSÉUM PÁRIS/Rio-Janeiro/de Castelnau”, [white handwritten] “f#”, [white handwritten] “Hilarianus/anguliceps Bl”, [red printed] “SYNTYPE”, [green handwritten] “H. anguliceps/Cat Mus/ Brésil/M. de Castelnau”.

##### Non-type material.

BRAZIL. ES: Rio Bonito, X/1963, 600 m, without collector, 1 ex. (DZUP); MG: Mesquita, XII/1973, Ravenna col., 1 ex. (CMNC); SP: São Paulo, IX/1936, J. Zikan col., 1 ex. (IBSP); without locality and date, Raben col., 1 ex. (ZMUC).

##### Diagnosis.

**Figure 69.**
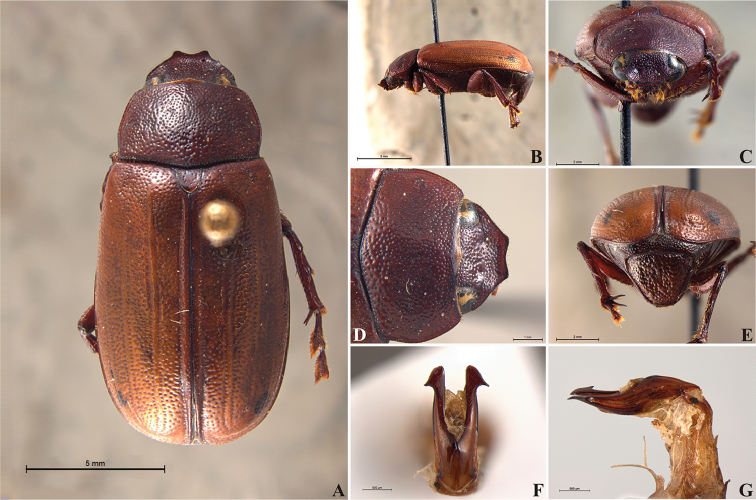
*Liogenys punctaticollis* Blanchard. **A** Dorsal view **B** Lateral view **C** Frontal view **D** Clypeus and pronotum **E** Pygidium **F** Parameres, dorsal view **G** Parameres, lateral view.

Body brownish; elongate; elytra testaceous to brownish, pronotum darker, reddish brown in males and dark brown in females; clypeal emargination sub-angled and wide; outer sides of anterior teeth sub-parallel; clypeal lateral margin straight; mesotibia quadrate in cross section in males, sub-quadrate to cylindrical in females; posterior margin of male metafemur medially produced on posterior margin; metatibial inner margin abruptly sub-basally or medially produced; pygidium varies from flat to convex, as wide as it is long, pygidial disc bristled only on apex; in males, total length of parameres near five times the length of their apex; inner margins convergent; apex harpoon-like with lateral angle curved projecting almost perpendicular to parameres (Fig. 69F).

##### Redescription.

Length: 14.0–16.0 mm; width: 6.9–7.9 mm. Brownish. *Head*: distance between eyes nearly twice the width of one eye; frons shorter than clypeus; clypeal emargination sub-angled, shallow and wide; outer sides of anterior teeth sub-parallel; outer margin of anterior tooth shorter than the eye; clypeal lateral margin straight; canthus not exceeding the outer margin of the eye; distal maxillary palpomere, maximum width less than twice width of apex; fovea shallow, extending past the transverse midline of the palpomere; labium transversely carinated, as wide as it is long; antenna 10-articulated, lamellae lighter in color and longer than flagellum. *Thorax*: anterior margin of pronotum slightly produced medially; maximum length of pronotum exceeding the length of tarsomeres I, II and III together; disc glabrous, punctures sparse and coarse; pronotal posterior corners sharp, almost right-angled; proepisternum with short bristles; mesepisternum scaly; sides of metasternum scaly and bristled, few long bristles on the anterior margin; distance between meso- and metacoxae up to twice longer than the metacoxa; scutellum ogival, finely punctured at the sides. *Elytra*: shiny, glabrous, uniform brownish, barely lighter in color than the pronotum; elytra more than three times longer than the pronotum; elytral suture slightly darker than elytron and distinctly elevated; pair of inner ridges more noticeable than outer three pairs. *Legs*: procoxa bristled on infra-carinal and outer surface; punctures visible at 12× magnification; three protibial teeth, middle and apical equal in size, the three teeth equally spaced; protibial inner apical spur present; mesofemural disc setose, with a row of long bristles on anterior and posterior margins; mesotibia sub-quadrate in cross section; disc coarsely sculptured, apical transverse carina in male mesotibia with intraspecific variation being partial or complete, in females always complete; basal apophysis of metacoxa produced beyond the outer margin of trochanter; male metafemur medially produced on posterior margin; metatibia with posterior discontinuous longitudinal carina; metatibial apical spurs equal in length, length equal to the diameter of the tibial apex; inner margin of male metatibia carinated and abruptly medially produced towards apex, apical inner surface setose, metatibial disc finely sculptured; metatibial transverse carina present posteriorly; basal metatarsomere and tarsomere II equal in size, in males protarsomere II long; pro- and mesotarsomeres I to IV enlarged, protarsomeres slightly wider than the mesotarsomeres and more than twice as wide as metatarsi; claw bifid, symmetrical, superior tooth longer and as wide as the inferior; distance between teeth shorter than the inferior tooth. *Abdomen*: ventrites bristled on disc; propygidium visible, glabrous; pygidium in lateral view flat or convex, in posterior view sub-trapezoidal, as wide as it is long; pygidial width not exceeding distance between spiracles of propygidium, pygidial disc bristled only on apex; pygidial apex in males quadrate. *Parameres*: width of basal region equal to the parameres together at its transverse midline, parameral split at the third portion; total length of parameres near five times the length of their apex; inner margins slightly convergent and opened; apex harpoon-like with lateral angle curved projecting almost perpendicular to parameres (Fig. 69F). In lateral view parameres concave (Fig. 69G).

##### Type-locality.

*Liogenys punctaticollis*: BRAZIL; *Liogenys anguliceps*: BRAZIL. Nova Friburgo, RJ (syn). *Liogenys palmata*: BRAZIL, “int” (syn).

##### Geographical distribution.

BRAZIL (**ES**, **MG**, RJ, **SP**).

##### Remarks.

*Liogenys punctaticollis* differs from *L. tibialis* (Fig. 77) in a few features, such as the lateral margin of clypeus being always straight; in females the elytra are very shiny and the male genitalia with total length of parameres near five times the length of their apex; apex harpoon-like with lateral angle curved projecting almost perpendicular to parameres and inner margins slightly convergent and opened.

In Cherman et al. (2016) *Hilarianus anguliceps* was synonymized with *L. punctaticollis*. As it is the *Hilarianus* type species, this genus was designated the junior synonym of *Liogenys* (Cherman et al. 2016).

#### Liogenys rufocastanea

Taxon classificationAnimaliaColeopteraMelolonthidae

Moser, 1921

Fig. 70

Liogenys rufocastaneus Moser, 1918: 103 (orig. desc.); Blackwelder 1944: 228 (check.); Frey 1969: 42 (key); Evans 2003: 214 (check.); Evans and Smith 2005: 178 (check.); Evans and Smith 2009: 182 (check.).

##### Type material.

*Liogenys rufocastaneus* male syntype (ZMHB): [white printed] “Paraguay”, [white handwritten] “Liogenys/rufocastaneus/Mos/Type m#.”, [light red printed] “Typus”, [white printed] “Liogenys/rufocastaneus/Mos.”. Genitalia mounted. This type is here designated the **lectotype**: [white, outlined in red, printed] “LECTOTYPE/*Liogenys rufocastaneus*/Moser, 1921/des. M. A. Cherman 2016”. A female syntype (ZMHB): [white printed] “Brasilien”, [white handwritten] “Liogenys/rufocastaneus/Mos/Type f#.” [light red printed] “Typus”, [white printed] “Liogenys/rufocastaneus/Mos.” These syntype are here designated as **paralectotype**, with the label: “PARALECTOTYPE/*Liogenys rufocastaneus*/ Moser, 1921/ des. M. A. Cherman 2016”.

##### Non-type material.

without locality and date, Kraatz, 1 ex. (SDEI); PARAGUAY. Without date, Fry, 1 ex. (NHMB); Without date, Kraatz, 9 ex. (SDEI). BRAZIL. Without date, Kraatz, 7 ex. (SDEI).

##### Diagnosis.

**Figure 70.**
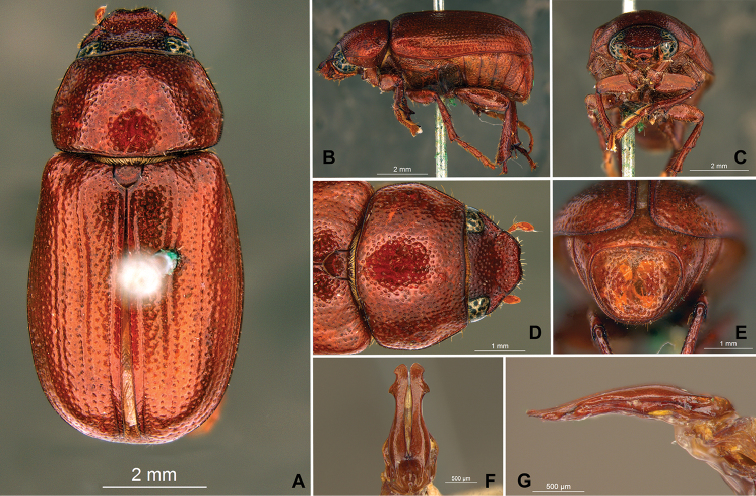
*Liogenys rufocastanea* Moser. **A** Dorsal view **B** Lateral view **C** Frontal view **D** Clypeus and pronotum **E** Pygidium **F** Parameres, dorsal view **G** Parameres, lateral view.

Body, elytra and pronotum brown; elongate; clypeus weakly emarginate; clypeus and frons almost coplanar; outer sides of anterior teeth follow the lateral margin of clypeus; clypeal lateral margin straight; pronotal posterior corners rounded; mesotibia cylindrical in cross section; pygidium flat and wide; pygidial width exceeding distance between spiracles of propygidium; pygidial disc bristled only on apex, punctures very sparse; pygidial apex quadrate; males genitalia, basal region of parameres wider than the parameres together at its maximum width; apex ornamented and emarginate laterally forming two projections at each side (Fig. 70F), subapical angles sharp, truncated and slightly divergent apically.

##### Redescription.

Length: 8.0-9.0 mm; width: 4.1-4.2 mm. Brown. *Head*: distance between eyes nearly twice the width of one eye in males, wider in females; frons shorter than clypeus; clypeus and frons almost coplanar; anterior margin of clypeus slightly emarginate, emargination shallow, rounded and wide; outer sides of anterior teeth follow the lateral margin of clypeus; clypeal lateral margin straight; canthus not exceeding the outer margin of the eye; distal maxillary palpomere, maximum width twice width of apex; fovea deep, extending past the transverse midline of the palpomere; labium transversely carinated, as wide as it is long; antenna 10-articulated, lamellae lighter in color and longer than flagellum. *Thorax*: anterior margin of pronotum slightly produced medially; maximum length of pronotum exceeding the length of tarsomeres I, II and III together, disc glabrous, punctures very sparse and coarse; pronotal posterior corners rounded; proepisternum with short bristles; mesosternum scaly; sides of metasternum with sparse scales anteriorly, few long bristles on the anterior margin; distance between meso- and metacoxae up to twice longer than the metacoxa; scutellum ogival, sparse and coarse punctures at the sides of the base. *Elytra*: shiny, glabrous, uniform brown; elytra more than three times longer than the pronotum; elytral suture and elytron unicolored, distinctly elevated; all four elytral ridges barely noticeable. *Legs*: procoxa, sparse scales on infra-carinal, outer surface up to inner margins of femur, smooth at 12× magnification; three protibial teeth, middle and apical equal in size, the three teeth equally spaced; protibial inner apical spur present; mesofemural disc setose, with a row of long bristles on the anterior margin; mesotibia cylindrical in cross section, disc finely sculptured, two mesotibial transverse carinae, the apical one incomplete in males; basal apophysis of metacoxa produced beyond the outer margin of trochanter, disc scaly; inner margin of metatibia carinated towards apex, apical inner surface setose; metatibial disc finely sculptured; metatibial transverse carina present posteriorly; metatibial apical spurs of different lengths, the longest shorter than the diameter of the tibial apex; protarsomere II long; in males pro- and mesotarsomeres I to IV enlarged, protarsomeres wider than the mesotarsomeres, twice as wide as metatarsi; basal metatarsomere twice shorter and slightly wider than tarsomere II; claw bifid, symmetrical, superior tooth longer and narrower than the inferior; distance between teeth shorter than the inferior tooth. *Abdomen*: ventrites bristled sparsely on disc and sides; propygidium visible, glabrous or scarcely bristled; pygidium flat, sub-trapezoidal, wide, as longer as wide, pygidial width exceeding distance between spiracles of propygidium; pygidial disc bristled only on apex; pygidial apex quadrate. *Parameres*: basal region wide and somewhat swollen, very short, parameral split at 1/3; ventral sides of base projected laterally; parameral inner margins straight; apex ornated, emarginate laterally forming two projections at each side, the basal one sharp, apical one truncated and slightly divergent (Fig. 70F). In lateral view parameres straight almost coplanar with basal region (Fig. 70G).

##### Type locality.

PARAGUAY.

##### Geographical distribution.

BRAZIL; PARAGUAY.

##### Remarks.

*Liogenys rufocastanea* resembles *Pacuvia castanea* Curtis —the species type of the Chilean genus— in the shape of the clypeus plus frons, which are almost coplanar and in the clypeus slightly emarginate, being this one a non-common feature among *Liogenys* species.

#### Liogenys santaecrucis

Taxon classificationAnimaliaColeopteraMelolonthidae

Blanchard, 1851

Figs 71 , 93

Liogenys santae-crucis Blanchard, 1851: 167 (orig. desc.)
Liogenys sanctae-crucis : Lacordaire 1856: 269 (sys.); Blackwelder 1944: 228 (check.)
Liogenys sanctae crucis : Harold 1869a:1140 (check.); Dalla Torre 1913: 318 (check.).
Liogenys sanctaecrucis : Frey 1969: 37 (key); Evans 2003: 214 (check.); Evans and Smith 2005: 178 (check.); Evans and Smith 2009: 182 (check.).
Liogenys excisus Moser, 1919: 15 (orig. desc.); Frey 1969: 43 (key); Blackwelder 1944: 227 (check.); Evans 2003: 208 (check.); Evans and Smith 2009: 181 (check.).
Liogenys peritryssoidea Keith, 2004: 195 (replacement name for Liogenys excisus Moser, 1919: 15); Evans and Smith 2005: 177 (check.); Evans and Smith 2009: 181 (check.) **Syn. n.**

##### Type material.

*Liogenys santaecrucis* male syntype (MNHN): [white handwritten] “D319/34” [light green printed] “MUSEUM PARIS/Chiquitos/d’Orbigny”, [red printed] “SYNTYPE”. This type is here designated the **lectotype** [white, outlined in red, printed] “LECTOTYPE/*Liogenys santaecrucis*/Blanchard, 1851/des. M. A. Cherman 2014”. Female syntype (MNHN) [white handwritten] “D319/34”, [light green printed] “MUSEUM PARIS/[handwritten] Chiquitos/d’Orbigny”, [red printed] “SYNTYPE”, [green handwritten] “L. sanctae-crucis/Cat. Mus./Santa-Cruz (Bolivie)/M. D Orbigny). This syntype is here designated the **paralectotype** [white, outlined in red, printed] “PARALECTOTYPE/*Liogenys santaecrucis*/Blanchard, 1851/des. M. A. Cherman 2014”.

*Liogenys excisus* male holotype (ZMHB): [white printed] “Cuyaba/Mtt. Grosso”, [white handwritten] “kein/furr? Mos”, [white handwritten] “Liogenys/excisus/Mos/Typen”, [red printed] “Typus”, [white handwritten] “Liogenys/bidenti/ceps Mos/[printed] det. G. Frey, 1968”.

##### Non-type material.

BRAZIL. MT: Dist. Guia. Faz Santhidi, 15°28'47S 56°07'33"W, 3/XI/2010, 180m, L. Silva col., 2 ex. (CEMT); Cáceres, 14/XI/1984, Buzzi, Mielke, Elias and Casagrande cols. 1 ex. (DZUP). BOLIVIA. LP: Rio Tamampayo, without date and collector, 1 ex. (ZMHB).

##### Diagnosis.

**Figure 71.**
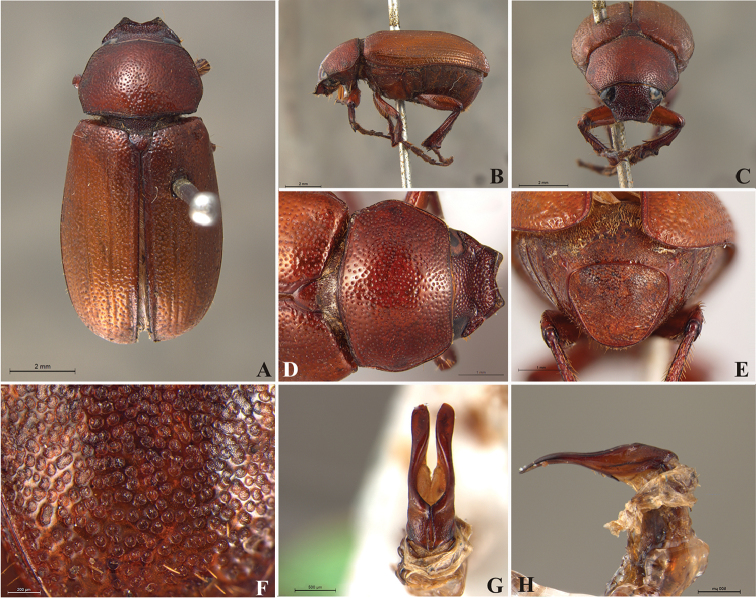
*Liogenys santaecrucis* Blanchard. **A** Dorsal view **B** Lateral view **C** Frontal view **D** Clypeus and pronotum **E** Pygidium **F** Reticulate punctures **G** Parameres, dorsal view **H** Parameres, lateral view.

Body brownish; elongate, sides almost parallel; elytra testaceous to brownish, pronotum slightly darker; clypeal emargination very deep, sub-angled and wide; outer sides of anterior teeth sub-parallel; meso- and metatibia quadrate in cross section, metatibia not transversally carinated and metatibial spurs equal in length; pygidial width exceeding distance between spiracles of propygidium, pygidial disc entirely bristled throughout. In males, pygidium covered with erect bristles, apex with angled corners. Male genitalia with a groove across the parameral basal region, elevated flange along the inner margin, apex widened and rounded (Fig. 71G).

##### Redescription.

Length: 9.5–10.3 mm; width: 4.4–5.2 mm. Brownish. *Head*: distance between eyes nearly twice the width of one eye; frons equal in length to clypeus; clypeal emargination deep, sub-angled and wide; teeth closer in females; outer sides of anterior teeth sub-parallel; outer margin of anterior teeth as long as the eye; lateral margin slightly convex; canthus not exceeding the outer margin of the eye; distal maxillary palpomere, maximum width equal to the apex; fovea deep, extending past the transverse midline of the palpomere; labium transversely carinated, as wide as it is long; antenna 10-articulated, lamellae lighter in color than flagellum and equal in length in males. *Thorax*: anterior margin of pronotum slightly produced medially; maximum length of pronotum exceeding the length of tarsomeres I, II and III together; disc glabrous, punctures coarse and sparse; pronotal posterior corners sharp, right-angled; proepisternum with long bristles, pro- and mesepisternum scaly, as are the sides of metasternum, also with few long bristles on the anterior margin; distance between meso- and metacoxae up to twice longer than the metacoxa; scutellum ogival, coarsely punctured at the base or sides. *Elytra*: shiny, glabrous, uniformly testaceous, barely lighter in color than pronotum, elytra more than three times longer than the pronotum; elytral suture slightly darker than elytron and not elevated; all four elytral ridges barely noticeable. *Legs*: procoxa scaly on infra-carinal and outer surface; punctures visible at 12× magnification; three protibial teeth, middle and apical equal in size; the three teeth equally spaced; protibial inner apical spur present; mesofemural disc setose, with a row of long bristles on anterior and posterior margins; mesotibia quadrate in cross section; disc finely sculptured; basal apophysis of metacoxa produced beyond the outer margin of trochanter; metatibial disc finely sculptured and posterior discontinuous longitudinal carina; inner margin of male metatibia carinated towards apex, apical inner surface setose; metatibia not carinated transversally; metatibial apical spurs equal in length, length equal to the apex diameter of tibia; in males, pro- and mesotarsomeres I to IV enlarged, protarsomeres slightly wider than the mesotarsomeres; protarsi more than twice wider than metatarsi; basal metatarsomere and tarsomere II equal in size; claw bifid, symmetrical, superior tooth longer and narrower than inferior tooth; distance between teeth less than the inner tooth. *Abdomen*: band of scales visible at the lowest magnification beneath the outer margin of elytra; disc and sides of ventrites bristled as is the propygidium; pygidium flat, trapezoidal, apex somewhat angled, wider than long, pygidial width exceeding distance between spiracles of propygidium; pygidial disc bristled throughout, with yellow bristles, deep umbilical reticulate punctures (Fig. 71F). *Parameres*: width of basal region equal to the parameres together at its maximum width; parameral split at 2/3; basal region strongly grooved at the split level and follows the inner margin forming a flange (Fig. 71G); apex rounded and widened. In lateral view parameres concave, curved downwards apically (Fig. 71H).

##### Type locality.

*Liogenys santaecrucis*: BOLIVIA. Santa-Cruz, Chiquitos; *Liogenys excisus*: BRAZIL. Mato Grosso, Cuiabá (**junior synonym**).

##### Geographical distribution.

BRAZIL (MT); BOLIVIA (**LP**, SC).

##### Remarks.

*Liogenys santaecrucis* shares with *L. bidenticeps* the body size and color of elytra, but it differs in many features such as the deep and angled clypeal emargination, outer margin of teeth and eye equally long, metatibial spurs equally long, pygidium exceeding the distance between the spiracles of the propygidium and the shape of male genitalia is also distinctive. It shares with *L. bilobata* and *L. diodon* the metatibia not carinated transversally and with discontinuous longitudinal carina posteriorly (Figs 11–12, 59, 62). This species is extremely similar to *L. pseudosanctaecrucis* Cherman, sp. n. *Liogenys santaecrucis* differs in the basal metatarsomere being equal in length to tarsomere II, pygidial disc bristled throughout and parameres with a narrower groove crossing the basal region at the split level. *Liogenys perytrissoidea* is the replacement name for *L. excisus* Moser, 1919 designated by Keith (2004). The type of *Liogenys excisus* bears the label [white handwritten] “Liogenys/bidenti/ceps Mos/[printed] det. G. Frey, 1968” because Frey (1969) suggested that *L. excisus* could be a synonym of *L. bidenticeps*. That synonymy inferred by Frey (1969) is discarded in the present work. Male primary types of *L. santaecrucis* (MNHN) and *L. peritryssoidea* (ZMHB) were compared, and we concluded that it is a **junior subjective synonym** of *L. santaecrucis*. Blanchard's, species name was originally *Liogenys santae-crucis*. Subsequent authors corrected its spelling to *L. sanctaecrucis*. In this work, the spelling is being corrected again to *L. santaecrucis*, maintaining the root of the original species epithet.

#### Liogenys sinuaticeps

Taxon classificationAnimaliaColeopteraMelolonthidae

Moser, 1918

Figs 72 , 91

Liogenys sinuaticeps Moser, 1918: 104 (orig. desc.); Blackwelder 1944: 228 (check.), Frey 1969: 47 (key); Evans 2003: 214 (check.); Evans and Smith 2005: 178 (check.); Evans and Smith 2009: 182 (check.).

##### Type material.

*Liogenys sinuaticeps* male syntype (ZMHB): [white printed] “Brasilia”, [white handwritten] “Liogenys/sinuaticeps/Mos/Typen m#.”, [red printed] “Typus”, [white printed] “Liogenys/sinuaticeps/Mos.”, [red printed] “SYNTYPUS/Liogenys/sinuaticeps Moser, 1918/labelled by MNHUB 2014”. Genitalia mounted. As according to Moser's, description there is only one type specimen, it is considered the holotype.

##### Non-type material.

BRAZIL. SP: Campinas, without date and collector, 1 ex. (ZMHB); RS: Itacurubi, 10/X/2012, I. Valmorbida col, 1 ex. (DZUP).

##### Diagnosis.

**Figure 72.**
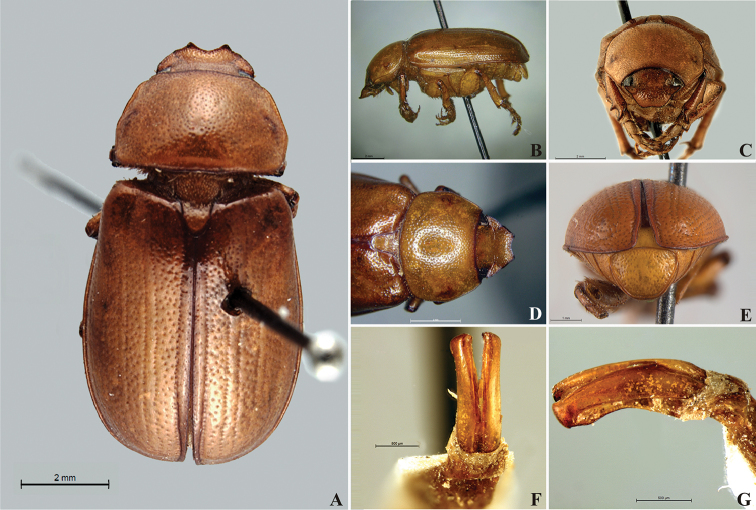
*Liogenys sinuaticeps* Moser. **A** Dorsal view **B** Lateral view **C** Frontal view **D** Clypeus and pronotum **E** Pygidium **F** Parameres, dorsal view **G** Parameres, lateral view.

Body yellowish; elongate; elytra and pronotum uniform yellow; clypeal emargination rounded and wide; outer sides of anterior teeth sub-parallel; clypeal lateral projection barely noticeable, rounded; canthus exceeding the outer margin of the eye; labium not transversely carinated, longer than wide, labial sides swollen; sensorial area not reaching the midline of the palpomere and not forming a fovea; antenna 9-articulated; basal apophysis of metacoxa not produced; metafemur with thick and erect bristles on posterior margin; inner margin of male metatibia not carinated and not produced on apex, metatibial inner face glabrous; tarsi opaque, male protarsomeres enlarged as wide as the mesotarsomeres, more than twice as wide as metatarsi; pygidial disc glabrous; flat in lateral view; male genitalia, parameral width almost equal throughout their entire length; inner margins straight or slightly divergent, apex of parameres truncated and curved downwards (Fig. 72F).

##### Redescription.

Length: 9.8-10.1 mm; width: 5.1-5.2 mm. Yellowish. *Head*: distance between eyes more than three times wider than one eye; frons equal in length to clypeus; clypeal emargination shallow, rounded and wide; outer sides of anterior teeth sub-parallel; outer margin of anterior teeth longer than the eye; clypeal lateral margin convex, with a rounded projection barely noticeable; distance between clypeal lateral projection and anterior margin of eye longer than one eye; distance between clypeal lateral projection and anterior tooth shorter than basal width of anterior tooth; canthus exceeding the outer margin of the eye; maxilla, teeth of galea reduced; distal maxillary palpomere, maximum width barely wider than apex; sensorial area not reaching the midline of the palpomere and not forming a fovea; labium not transversely carinated, longer than wide, labial sides swollen; antenna 9-articulated, lamellae unicolored with flagellum and in males they are equal in length. *Thorax*: anterior margin of pronotum straight; maximum length of pronotum exceeding the length of tarsomeres I, II and III together; disc glabrous, punctures fine and very sparse; pronotal posterior corners rounded; proepisternum bristled; mesepisternum scaly; sides of metasternum glabrous; distance between meso- and metacoxae up to twice longer than the metacoxa; scutellum ogival, coarsely punctured at the base or sides. *Elytra*: shiny, glabrous, uniform yellowish brown; elytra more than three times longer than the pronotum; elytral suture and elytron unicolored, distinctly elevated; the two pairs of inner ridges more noticeable than the two outer pairs. *Legs*: procoxa scaly on infra-carinal surface and bristled on the outer one; smooth at 12× magnification; three protibial teeth, the apical the longest, the three teeth equally spaced; protibial inner apical spur present; mesofemural disc setose; mesotibia cylindrical in cross section; disc finely sculptured, two mesotibial transverse carinae, the apical one incomplete; basal apophysis of metacoxa not produced; metafemur with thick and erect bristles on posterior margin; metatibial apical spurs of different lengths, the longest one exceeding the diameter of the tibial apex; inner margin of metatibia not carinated and not produced on apex; metatibial disc finely sculptured; two metatibial transverse carinae present posteriorly; tarsi opaque; basal metatarsomere shorter and wider than tarsomere II; in males protarsomere II short and wide; pro- and mesotarsomeres I to IV enlarged, protarsomeres as wide as the mesotarsomeres, more than twice as wide as metatarsi; claw bifid, symmetrical, superior tooth longer and as wide as the inferior; distance between teeth longer than the inferior tooth. *Abdomen*: band of scales visible at the lowest magnification beneath the outer margin of elytra; ventrites bristled on disc; propygidium visible, glabrous; pygidium flat, sub-trapezoidal, wider than long; pygidial width not exceeding distance between spiracles of propygidium; pygidial disc glabrous; pygidial apex quadrate or sub-quadrate. *Parameres*: width almost equal throughout the entire length of the parameres; parameral split at the third portion; inner margins straight, slightly divergent; parameres apex truncated (Fig. 72F). In lateral view parameres slightly convex, apex curved downwards (Fig. 72G).

##### Type-locality.

BRAZIL.

##### Geographical distribution.

BRAZIL (**SP**, RS).

##### Remarks.

*Liogenys sinuaticeps* is a singular species, distinguished from the other *Liogenys* by its labium longer than wider, without concavity on disc (as in *Pacuvia*) (Fig. 24), and the five teeth of the maxilla reduced (Fig. 27). *Liogenys sinuaticeps* shares with *L. laminiceps* and *L. flavida* in having antenna 9-articulated, disagreeing with Moser (1918) who states that the antenna is 10-articulated. This species is closely related to *L. unicolor* (Fig. 78) (Cherman et al. 2016) and they share the following features: sensorial area of the maxillar distal palpomere not forming a fovea (Fig. 28), this feature also in common with *L. macropelma*; pygidial disc glabrous and the basal apophysis of metacoxa not produced, also in common with *L. tarsalis* (Fig. 75).

#### Liogenys spiniventris

Taxon classificationAnimaliaColeopteraMelolonthidae

Moser, 1918

Figs 73 , 90

Liogenys spiniventris Moser, 1918: 108 (orig. desc.); Blackwelder 1944: 228 (check.); Frey 1969: 47 (key); Evans 2003: 214 (check.); Evans and Smith 2005: 178 (check.); Evans and Smith 2009: 182 (check.).

##### Type material.

*Liogenys spiniventris* male syntype (ZMHB): [white printed] “Brasilia/[handwritten]Bahia”, [white handwritten] Liogenys/spiniventris/Mos./Typen”, [red printed] “Typus”, [white printed] “Liogenys/spiniventris/Mos.”, [red printed] “SYNTYPUS/Liogenys/spiniventris Moser, 1918/labelled by MNHUB 2014”. As it is the unique primary type, it is considered the holotype.

##### Diagnosis.

**Figure 73.**
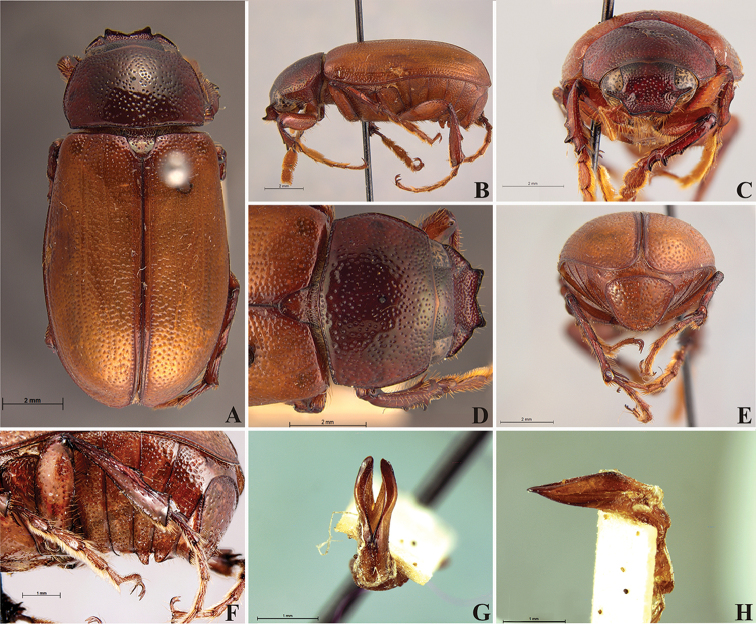
*Liogenys spiniventris* Moser. **A** Dorsal view **B** Lateral view **C** Frontal view **D** Clypeus and pronotum **E** Pygidium **F** Abdomen, lateral view **G** Parameres, dorsal view **H** Parameres, lateral view.

Body brownish; elongate; elytra testaceous, pronotum purplish brown, clypeal emargination sharp and wide; outer sides of anterior teeth parallel; clypeal lateral margin convex, with a tooth-like projection; right angle between outer side of anterior teeth and clypeal lateral projection; mesotibia quadrate in cross section; inner margin of metatibia abruptly sub-basally produced towards apex; ventrites IV and V produced medially (Fig. 73F); pygidium convex, as wide as it is long; pygidial disc bristled only on apex; parameres, width of basal region narrower than the parameres together at its maximum length, apex fusiform (Fig. 73G).

##### Redescription.

Length: 12.5 mm; width: 6.1 mm. Brownish. *Head*: distance between eyes nearly twice the width of one eye; frons swollen, equal in length to clypeus; clypeal emargination sharp, shallow and wide; outer sides of anterior teeth parallel; outer margin of anterior teeth shorter than the eye; clypeal lateral margin convex forming a tooth-like projection; distance between clypeal lateral projection and anterior margin of eye shorter than one eye; distance between clypeal lateral projection and anterior tooth shorter than basal width of anterior tooth; right angle between outer side of anterior teeth and clypeal lateral projection; canthus not exceeding the outer margin of the eye; distal maxillary palpomere, maximum width less than twice width of apex; fovea shallow, extending past the transverse midline of the palpomere; labium transversely carinated, as wide as it is long; antenna 10-articulated, lamellae lighter in color and longer than flagellum. *Thorax*: anterior margin of pronotum slightly produced medially; maximum length of pronotum exceeding the length of tarsomeres I, II and III together; disc glabrous, punctures fine and sparse; pronotal posterior corners sharp, almost right-angled; proepisternum with short bristles; mesepisternum scaly; sides of metasternum scaly and bristled, few long bristles on the anterior margin; distance between meso- and metacoxae up to twice longer than the metacoxa; scutellum ogival, finely punctured at the sides. *Elytra*: shiny, glabrous, uniformly testaceous, distinctly lighter in color than pronotum, elytra more than three times longer than the pronotum; elytral suture slightly darker than elytron and distinctly elevated; all four elytral ridges barely noticeable. *Legs*: procoxa bristled on infra-carinal and outer surface; punctures visible at 12× magnification; three protibial teeth, middle and apical equal in size, the three teeth equally spaced; protibial inner apical spur present; mesofemural disc setose, with a row of long bristles on anterior and posterior margins; mesotibia quadrate in cross section, disc coarsely sculptured; two mesotibial transverse carinae, the apical one incomplete; basal apophysis of metacoxa produced beyond the outer margin of trochanter; inner margin of metatibia carinated and abruptly sub-basally or medially produced up to the apex, apical inner surface setose; metatibial disc finely sculptured; metatibial transverse carina present posteriorly and posterior discontinuous longitudinal carina; metatibial spurs equal in length, length equal to the diameter of the tibial apex; basal metatarsomere and tarsomere II equal in size, slightly wider than the mesotarsomeres and more than twice as wide as metatarsi; claw bifid, symmetrical, superior tooth longer and as wide as the inferior; distance between teeth shorter than the inferior tooth. *Abdomen*: ventrites bristled on disc and sides; ventrites IV and V produced medially; propygidium visible, glabrous; pygidium convex; sub-trapezoidal, as wide as it is long; pygidial width not exceeding distance between spiracles of propygidium; pygidial disc bristled only on apex; pygidial apex quadrate. *Parameres*: width of basal region narrower than the parameres together at its maximum width; parameral split at the third portion; inner margins of parameres convergent; apex fusiform (Fig. 73G). In lateral view parameres straight not coplanar with basal region (Fig. 73H).

##### Type-locality.

BRAZIL. Bahia.

##### Geographical distribution.

BRAZIL (BA).

##### Remarks.

*Liogenys spiniventris* resembles *L. testaceipennis* (Fig. 76). The former differs in the shape of the clypeus, sharply emarginate, with a tooth-like projection laterally and in ventrites with different number of the medial projections (Figs 73, 76F). Female of *L. spiniventris* remains unknown.

#### Liogenys suturalis

Taxon classificationAnimaliaColeopteraMelolonthidae

(Blanchard, 1851)

Figs 74 , 92

Hilarianus suturalis Blanchard, 1851: 169 (orig. desc.); Lacordaire 1856: 270 (sys.); Harold 1869a: 1141 (check.); Dalla Torre 1913: 319 (check.); Blackwelder 1944: 228 (check.).
Liogenys suturalis : Frey 1969: 40, 55 (key, n. comb.); Evans 2003: 214 (check.); Evans and Smith 2005: 178 (check.); Evans and Smith 2009: 182 (check.)

##### Type material.

*Hilarianus suturalis* female syntype (MNHN): [light green printed] “MUSÉUM PÁRIS/ Bresil/deCastelnau”, [red printed] “SYNTYPE”, [green handwritten] “H. suturalis/ Cat Mus/ Minas-Geraes (Brésil)/ M. de Castelnau”. This type is here designated the **lectotype** [white, outlined in red, printed] “LECTOTYPE/*Hilarianus suturalis*/Blanchard, 1851/des. M. A. Cherman 2014”. Female syntype (MNHN): [white handwritten] “1859/34”, [white handwritten] “2860”, [light green printed] “MUSÉUM PÁRIS/ [handwritten] Santa-Cruz/d’Orbigny”, [red printed] “SYNTYPE”, [green handwritten] “H. suturalis/ Cat Mus/ Santa-Cruz (Bolivie)/ M. D’Orbigny”. This type is here designated the **paralectotype** [white, outlined in red, printed] “PARALECTOTYPE / *Hilarianus suturalis*/Blanchard, 1851/des. M. A. Cherman 2014”.

##### Non-type material.

BRAZIL. GO: Edéia, 14/X/2003, R. B. da Costa col., 1 ex. (CEMT); Goiania, X/2012, Tissiane Col., 2 ex. (CEMT); DF: Brasília, “Lago Norte”, 15°45'42.63"S, 47°50'5.8"W, XII/2009, 1023 m, Nunes col., 6 ex. (CEMT); Planaltina, 15°36'16"S, 47°44'16"W, 3/XI/2006, C. Oliveira col., 5 ex. (CEMT); MT: Rosário Oeste, XI/1976, A. Maller col., 2 ex. (MNRJ); Córrego Brigadeiro [Figueirópolis D’Oeste], 15°30’S, 58°43’W, 29/IX/1984, Binda col., 7 ex. (INPA); 29/IX/1984, Marcolino col., 1 ex. (CEMT); BR 270 km 140 [near Rondonópolis], 27/IX/1984, Binda col., 1 ex. (INPA); Chapada dos Guimarães, 23/X/2011, Tassinari col., 1 ex. (CEMT); Cuiabá, without date and collector, 1 ex.; 24/VIII/1993, Pinto Col., 1 ex.; 20/VI/2010, Rabello col., 1 ex.; 13/XI/2012, Jacobina col., 1 ex.; 3/X/1988, Silva col., 1 ex.; 9/IX/1990, Netto col., 1 ex.; 21/V/1986, Costa col., 1 ex. (CEMT); Poconé, 15/IV/2009, Abreu col., 1 ex.; 9/XI/2002, without collector, 1 ex.; 21/XI/2003, without collector, 1 ex. (CEMT); Varzea Grande, 13/XI/1993, Paula col., 1 ex. (CEMT); Campo Verde, “Rio Engano”, 14/X/2012, Pena and Pena cols., 1 ex. (CEMT); Barra do Bugre, 7/XI/1993, Serrano col., 1 ex. (CEMT); MS: Campo Grande, X/1947, A. Maller col., 2 ex. (AMNH); Porto Murtinho, XI/1929, W. Mehr col., 14 ex. (MNRJ); Dourados, 22°13'44.26"S, 54°49'48,18"W, 15/VIII/2010, 429 m, Simioni col. 1 ex.; “Faz. Coqueiro Azulão”, 22°14'28"S, 54°55'08"W, 12/IV/2012, D. G. Ribeiro col., 1 ex.; “UFGD”, 22°11'38,7"S, 54°55'16"W, 9/V/2012, D. G. Ribeiro col., 1 ex. (MuBio); 26/XI/2012, F. Vaz-de-Mello col., 3 ex. (CEMT); Nova Andradina, “Usina Santa Helena”, 21°59'32"S, 53°26'28"W, 18/X/2012, 360 m, G. Coutinho col., 4 ex. (CEMT); Rio Brilhante, 21-28/X/1970, V. O. Becker col., 3 ex. (DZUP); Bonito, 21°07'44"S, 56°28'46"W, 19/XII/2010, F. Vaz-de-Mello col., 1 ex. (CEMT); Rio Verde [Rio Verde do Mato Grosso], XI/1959, 400 m, without collector, 2 ex.; X/1966, A. Maller col., 1 ex. (DZUP); BA: Encruzilhada, 15°31'42,3"S, 40°57'52,1"W, 16/XII/2012, 780 m, Rafael and Grossi col., 1 ex.; 15°34'35"S, 40°56'51"W, 16/XII/2012, 850 m, Rafael and Grossi col., 1 ex. (INPA); XI/1975, without collector, 4 ex. (MNRJ); “Estr. SE”, 16/XII/2012, P. Grossi col., 4 ex. (EPGC); MG: Aguas vermelhas, XII/1997, Vaz-de-Mello and Bello cols., 2 ex. (CEMT); 12/XII/2012, P Grossi col., 14 ex. (EPGC); (1) São Roque de Minas, “Serra da Canastra, Sede Jaguaré”, 20°15'17"S, 46°25'11"W, 2/XI/2007, Souza col., 1 ex. (CEMT); Lavras, 9/X/2001, Vieira col., 1 ex. (CEMT); Uberaba, X/1961, D. Elias col., 15 ex. (DZUP); Uberlândia, 21/X/1974, H.N. Espínola col., 2 ex. (DZUP); Ibiá, 20/X/1965, C.T. and C. Elias col., 1 ex. (DZUP); SP: Pontal, 23/X/1984, Osman Dermeka col., 4 ex. (DZUP); Descalvado, “Faz. Itaunas”, 27/IX/2007, N. W. Perioto col., 2 ex.; 6/X/2006, N. W. Perioto col., 2 ex.; 17/XII/2007, N. W. Perioto col., 1 ex.; 5/XI/2007, N. W. Perioto col., 1 ex. (CEMT); Neves Paulista, 9/VII/2009, Fanti col., 1 ex. (CEMT); Palmital, “Faz. São Sebastião”, 12/X2010, Callil col., 18 ex. (CEMT); Batatais, 31/X/1969, L. C. Silva col., 1 ex.(DZUP); Ribeirão Preto, 31/X/1973, Pe. Moure col., 1 ex. (DZUP); PR: Porecatu, 20/X/1970, Becker-Hastchback col., 1 ex. (DZUP). BOLIVIA. SC: Comarapa, “Aserradero La forestal”, 9/XI/1987, D. Salvatierra col., 1 ex. (AMNH); Parapeti, I/1960, A. Martinez col., 1 ex. (CMNC); Camiri, 12/XI/1946, Maldonado col., 1 ex. (1) (ZMNC); CB: Cochabamba, without date, Zischka col., 1 ex. (CMNC). PARAGUAY. Without date, Zikan col., 1 ex. (CEIOC); without locality, date and collector, 10 ex.; “camino vogt”, without date, Heys col., 1 ex. (SDEI); CN: San Carlos del Apa, 22°14'04"S, 57°17'48"W, 30/X/2002, B. Garcete col., 2 ex. (CEMT); AS: Asunción, XI/1944, Mis. Cient. Brasil col., 3 ex. (CEIOC); IT: Coronel Bogado, 27°10'12"S 56°15'0"W, I/1944, A. Martinez col., 1 ex. (CEIOC); GU: Villarica, XII/1928, Schade col., 3 ex. (CEIOC). ARGENTINA. SA: El Naranjo, II/1944, A. Martinez col., 2 ex.; Parque Nacional El Rey, 15/XII/1987, 890 mm Peck col., 2 ex.; Tartagal, XI/1944, Duret and Martinez cols., 4 ex. (CMNC); Cafayate, 26°03'52"S, 65°56'19"W, 24/XI/2006, Ocampo, Ruiz, San Blas, Salazar cols., 3 ex. (IADIZA); JU: XI/1947, without collector, 2 ex. (DZUP); without date, Bruch col., 2 ex. (ZMHB); Marta [?], XI/1946, Duret col., 1 ex. (CMNC); FO: Laguna Yema, 24°19'54"S, 61°17'37,73"W, 11/XII/2008, 161 m, Ocampo, San Blas, Campon cols., 2 ex. (IADIZA); CH: XI/1945, A. Martinez col., 1 ex. (CMNC); CO: El Diquecito, I/1976, A. Martinez col., 2 ex.; San Javier, II/1946, Monros col., 1 ex.; Calamuchita, “La Granadilla”, I/1974, A. Martinez col., 1 ex. (CMNC); Lozada, XI/2006, P. Fichetti col., 3 ex. (IADIZA); “Cordova, Pampas”, without date and collector, 1 ex. (MNHN); SL: Luján, I/2010, M. A. Cherman col., 3 ex. (DZUP).

##### Diagnosis.

**Figure 74.**
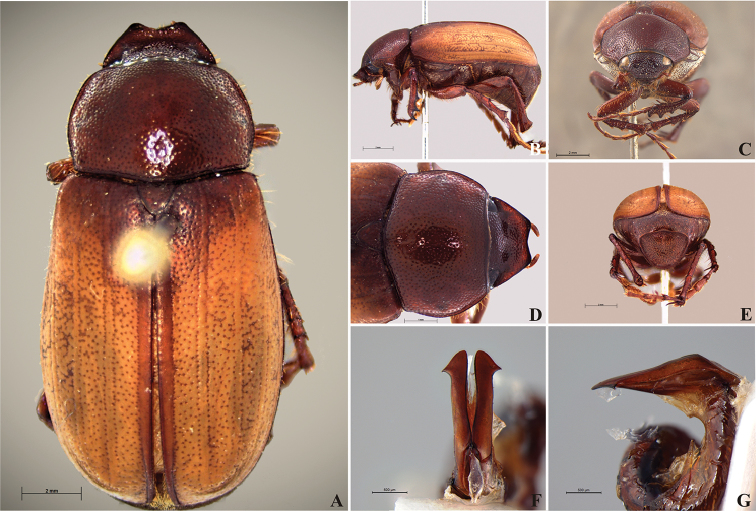
*Liogenys suturalis* (Blanchard). **A** Dorsal view **B** Lateral view **C** Frontal view **D** Clypeus and pronotum **E** Pygidium **F** Parameres, dorsal view **G** Parameres, lateral view.

Body brownish, elongate; elytra dull yellow, pruinose, opaque; pronotum reddish brown, shiny; clypeal emargination shallow, rounded and wide; outer sides of anterior teeth follow the lateral margin of clypeus, sub-parallel in females; lateral margin straight, slightly convex in females; pronotal posterior corners sharp, obtuse-angled; meso- and metepisternum, sides of metasternum and metacoxae scaly; mesotibia sub-quadrate in cross section; pygidium flat, pygidial disc bristled throughout, coarsely punctured; in males total length of parameres near five times the length of their apex; inner margins straight, slightly divergent; apex harpoon-like with lateral angle curved projecting almost perpendicular to parameres (Fig. 74F).

##### Redescription.

Length: 13.5–15.0 mm; width: 6.7–7.6 mm. Reddish brown and elytra dull yellow. *Head*: distance between eyes nearly twice the width of one eye, more than twice in females; frons equal in length to clypeus; clypeal emargination rounded, shallow and wide; outer sides of anterior teeth follow the lateral margin of clypeus in males, sub-parallel in females; clypeal lateral margin straight, slightly convex in females; outer margin of anterior tooth shorter than one eye; canthus not exceeding the outer margin of the eye; distal maxillary palpomere, maximum width twice width of apex; fovea deep, extending to the transverse midline of the palpomere; labium transversely carinated, as wide as it is long; antenna 10-articulated, lamellae lighter in color and longer than flagellum. *Thorax*: anterior margin of pronotum straight; maximum length of pronotum exceeding the length of tarsomeres I, II and III together; disc glabrous, punctures fine and dense, coarser in females; pronotal posterior corners sharp, obtuse-angled; proepisternum with long bristles; meso- and metepisternum, and sides of metasternum scaly, metasternum with few long bristles on the anterior margin; distance between meso- and metacoxae up to twice longer than the metacoxa; scutellum ogival, coarsely punctured. *Elytra*: glabrous, dull yellow, darker at the base, pruinose; elytra more than three times longer than the pronotum; elytral suture slightly darker than elytron and distinctly elevated; pair of inner ridges more noticeable than the three outer pairs. *Legs*: procoxa scaly on infra-carinal and outer surface; punctures visible at 12× magnification; three protibial teeth, middle and apical equal in size, the three teeth equally spaced; protibial inner apical spur present; mesofemural disc setose, with a row of long bristles on anterior and posterior margins; mesotibia sub-quadrate in cross section; disc coarsely sculptured; mesotibial apical transverse carina may vary from partial to complete, in females always complete; basal apophysis of metacoxa produced beyond the outer margin of trochanter; metatibia with posterior discontinuous longitudinal carina; metatibial apical spurs equal in length, length equal to the diameter of the tibial apex; inner margin of male metatibia carinated towards apex, apical inner surface setose; metatibial disc coarsely sculptured; two metatibial transverse carinae present posteriorly; basal metatarsomere and tarsomere II equal in size; in males protarsomere II long; pro- and mesotarsomeres I to IV enlarged, protarsomeres slightly wider than the mesotarsomeres, twice as wide as metatarsi; claw bifid, symmetrical, superior tooth longer and as wide as the inferior; distance between teeth as long as the inferior tooth. *Abdomen*: band of scales visible at the lowest magnification beneath the outer margin of elytra; disc of ventrites bearing sparse short bristles; propygidium visible, with scarce scales; pygidium generally flat, sub-quadrate, as wide as it is long; pygidial width not exceeding distance between spiracles of propygidium; pygidial disc bristled throughout, coarsely punctured; pygidial apex sub-quadrate, more rounded in females. *Parameres*: basal region slightly narrower than the width of the parameres together at its transverse midline, parameral split at 2/3; total length of parameres near five times the length of their apex; inner margins straight, slightly divergent; apex harpoon-like with lateral angle curved projecting almost perpendicular to parameres (Fig. 74F). In lateral view parameres straight not coplanar with basal region (Fig. 74G).

##### Type-locality.

BRAZIL. Minas Gerais.

##### Geographical distribution.

BRAZIL (GO, DF, MT, MS, **BA**, MG, **SP**, **PR**); **BOLIVIA (CB**, **SC); PARAGUAY (CN**, **AS**, **IT**, **GU)**; **ARGENTINA (SA**, **JU**, **FO**, **CH**, **CO**, **SL)**.

##### Remarks.

*Liogenys suturalis* together with *L. opacipennis* Frey, 1969 and *L. rectangula* Frey, 1969, are the only *Liogenys* species with body brownish and elytra opaque dull yellow. *Liogenys suturalis* is a closely related lineage to the clade *L. elegans* + (*L. tibialis* + *L. punctaticollis* + *L. spiniventris* + *L. testaceipennis*) (Cherman et al. 2016), and differs from them mainly in the elytra pruinose and the abundant scales covering the pro-, meso- and metasternum laterally.

#### Liogenys tarsalis

Taxon classificationAnimaliaColeopteraMelolonthidae

Moser, 1921

Figs 75 , 91

Liogenys tarsalis Moser, 1921a: 54 (orig. desc.); Blackwelder 1944: 228 (check.); Cherman et al. 2016: 23 (comb.n.).
Homoliogenys tarsalis : Gutiérrez 1952: 216 (generic description); Frey 1969: 44 (key); Evans 2003: 206 (check.); Evans and Smith 2005: 171 (check.); Evans and Smith 2009: 175 (check.).

##### Type material.

*Liogenys tarsalis* male syntype (ZMHB): [white printed] “Rio Jan”, [white handwritten] “Liogenys/tarsalis/ Mos/ Typen m#.”, [white handwritten] “Wagner.”, [red printed] “Typus”, [red printed] “SYNTYPUS/Liogenys/tarsalis Moser, 1921/labelled by MNHUB 2014”. This type is here designated the **lectotype** [white, outlined in red, printed] “LECTOTYPE/*Liogenys tarsalis*/Moser, 1921/ des. M. A. Cherman 2014”. Female syntype (ZMHB) [white printed] “Rio Jan”, [white handwritten] “Liogenys/tarsalis/ Mos/ Typen f#.”, [white handwritten] “Wagner.”, [red printed] “Typus”, [red printed] “SYNTYPUS/Liogenys/tarsalis Moser, 1921/labelled by MNHUB 2014”. Female syntype (ZMHB) with the same labels as the former, plus: [orange printed] “*Liogenys tarsalis* Moser Det. K. Katovich 02”. Male syntype (NHRS): [white printed] “Rio Jan”, [white handwritten] “Liogenys tarsalis n. sp. Mos. m#”, [white printed] “Wagner.”, [red printed] “Typus”, [white printed] “NHRS- JLKB 000021172”. Female syntype (NHRS): [white printed] “Rio Jan”, [white handwritten] “Liogenys tarsalis n. sp. Mos. f#”, [white printed] “Wagner.”, [red printed] “Typus”, [white printed] “NHRS- JLKB 000021173”. These four syntypes are here designated **paralectotypes**, each one with the label: [white, outlined in red, printed] “PARALECTOTYPE/*Liogenys tarsalis*/Moser, 1921/ des. M. A. Cherman 2014”.

##### Non-type material.

ARGENTINA. CH: Charata, X/1924, 4 ex. (MLPA); Pampa del infierno, IX/1982, A. Martinez col. 1 ex. (CMNC); SF: Chaco, XI/1905, C. Bruch col. 1 ex. (ZMHB).

##### Diagnosis.

**Figure 75.**
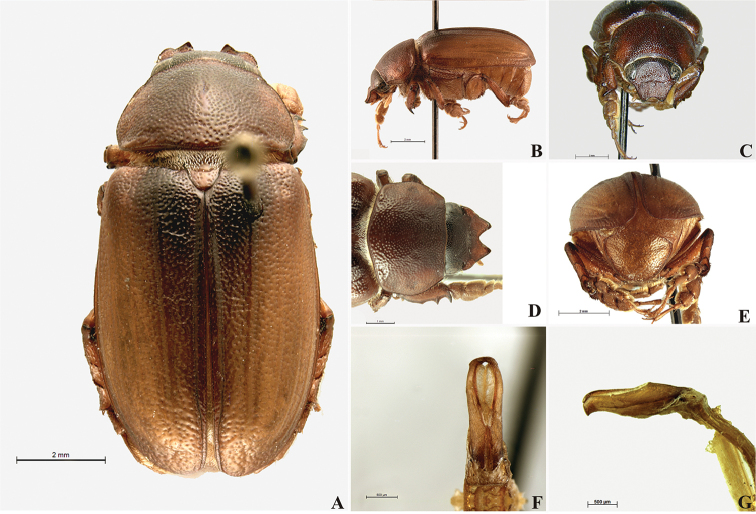
*Liogenys tarsalis* Moser. **A** Dorsal view **B** Lateral view **C** Frontal view **D** Clypeus and pronotum **E** Pygidium **F** Parameres, dorsal view **G** Parameres, lateral view.

Body brownish; elongate, sides almost parallel; elytra with non-uniform brown, base, inner and outer margins darker, as are the head and pronotum (Fig. 75A, C, D); clypeal emargination deep, sub-angled and narrow; outer sides of anterior teeth sub-parallel; clypeal lateral projection distinct, rounded; pronotum sulcated medially; protibial spur reduced or absent; mesofemur with thick erect bristles on posterior margin; basal apophysis of metacoxa not produced; apical inner surface glabrous; in males tarsi opaque and enlarged in all legs, mainly the tarsomere II; pygidium convex, bristled throughout; parameres with inner margins convergent, apex of each paramere fits into the other.

##### Redescription.

Length 10.0–10.5 mm; width: 5.2–5.4 mm. Brownish. *Head*: distance between eyes nearly twice the width of one eye; frons equal in length to clypeus; clypeal emargination deep, sub-angled and narrow; outer sides of anterior teeth sub-parallel; outer margin of anterior teeth as long as the eye; clypeal lateral margin convex, projection rounded; distance between clypeal lateral projection and anterior margin of eye longer than one eye; distance between clypeal lateral projection and anterior tooth shorter than basal width of anterior tooth; obtuse angle between outer side of anterior teeth and clypeal lateral projection; canthus not exceeding the outer margin of the eye; distal maxillary palpomere, maximum width more than twice width of apex; fovea shallow, extending past the transverse midline of the palpomere; labium transversely carinated, as wide as it is long; antenna 10-articulated, lamellae lighter in color and longer than the flagellum. *Thorax*: anterior margin of pronotum slightly produced medially; maximum length of pronotum exceeding the length of tarsomeres I, II and III together; disc sulcated medially, glabrous, punctures coarse and dense; pronotal posterior corners rounded; proepisternum with long bristles, pro- and mesepisternum scaly; sides of metasternum with sparse bristles and few long ones on the anterior margin; distance between meso- and metacoxae up to twice the length of the metacoxa; scutellum ogival to rounded, coarsely punctured. *Elytra*: shiny, glabrous, brownish, lighter in color than pronotum, base, inner and outer margins darker; elytra more than three times longer than the pronotum; elytral suture darker than elytron and elevated, pair of inner ridges more noticeable than the three outer pairs. *Legs*: procoxa scaly on infra-carinal and outer surface; punctures visible at 12× magnification; three protibial teeth, the apical the longest; distance between basal and middle teeth longer than between middle and apical; protibial inner apical spur reduced or absent; mesofemural disc setose, thick erect bristles on posterior margin; mesotibia cylindric in cross section, disc coarsely sculptured; two mesotibial transverse carinae, the apical one incomplete; metacoxa scaly, basal apophysis not produced; inner margin of metatibia carinated but apex not produced (see Cherman et al. 2016) and glabrous on inner surface; metatibial disc finely sculptured; metatibia posteriorly with two transverse carinae and posterior discontinuous longitudinal carina; metatibial apical spurs of different lengths; the longest equal in length to the diameter of the tibial apex; tarsi opaque; pro-, meso- and metatarsomeres I to IV equally enlarged in every leg; protarsomere II short and wide; basal metatarsomere shorter than tarsomere II; claw bifid, symmetrical, superior tooth longer and wider than the inferior, distance between teeth longer than the inferior tooth. *Abdomen*: ventrites bristled on disc and sides; pygidium convex, sub-trapezoidal, apex sub-angled to rounded, wider than long, pygidial width not exceeding distance between propygidial spiracles; pygidial disc bristled throughout, bristles longer on apex; male pygidial apex sub-quadrate to rounded. *Parameres*: basal region slightly narrower than the parameres together at its maximum width, parameral split at the third portion; inner margins convergent, apex curved inwards, fitting into each other (Figs 51, 75F). In lateral view parameres slightly concave.

##### Type-locality.

BRAZIL, Rio de Janeiro.

##### Geographical distribution.

BRAZIL (RJ); **ARGENTINA (CH**, **SF)**.

##### Remarks.

*Liogenys tarsalis* is the type species of *Homoliogenys*, a monotypic genus created by Gutiérrez (1952). Recently, Cherman et al. (2016), returned *L. tarsalis* to *Liogenys*, supported by phylogenetic evidence. As *Homoliogenys* is monotypic, this genus became a junior subjective synonym of *Liogenys*. This species shares various features with *Liogenys forcipata* Frey, as the clypeus strongly emarginate with a sharp lateral projection and the basal apophysis of metacoxa reduced, this one being a non-common feature within *Liogenys*. It differs from *L. forcipata* in the clypeal emargination more rounded, metasternum scarcely setose, scutellum more rounded, inner margin of metatibia not produced on apex and glabrous on inner surface and metatibia posteriorly as wide as or slightly narrower than the outer face. Males of *L. tarsalis* are also different from *L. forcipata* in the protibial teeth unequally spaced, tarsi opaque and all of them equally enlarged, protarsomere II short and wide, pygidium more rounded and parameres glabrous (Figs 51, 75F). The type-locality of *L. tarsalis* (Rio de Janeiro) is very far from those localities recorded among the non-type material which are all from Argentinian Chacoan Province (Fig. 91). The type material was collected by Wagner during the end of 19^th^ century and beginning of the 20^th^, mainly in Rio Salado region (SE, Argentina). Probably, the type-locality written on the original label was “Rio Sal” instead of “Rio Jan”, misspelled by the person who wrote the definitive label. Females remain unknown.

#### Liogenys testaceipennis

Taxon classificationAnimaliaColeopteraMelolonthidae

Moser, 1918

Figs 76 , 90

Liogenys testaceipennis Moser, 1918: 109 (orig. desc.); Blackwelder 1944: 228 (check.); Frey 1969: 47 (key); Evans 2003: 214 (check.); Evans and Smith 2005: 178 (check.); Evans and Smith 2009: 182 (check.)
Liogenys seabrai Martínez, 1957: 51 (orig. desc.); Evans 2003: 214 (check.); Evans and Smith 2005: 178 (check.); Evans and Smith 2009: 182 (check.) **Syn. n.**

##### Type material.

*Liogenys testaceipennis* male holotype (ZMHB): [white printed] “Brasil”, [white handwritten] “Liogenys/testaceipennis/Mos./Type”, [red printed] “Typus”, “HOLOTYPUS/Liogenys/testaceipennis Moser, 1918/labelled by MNHUB 2013”. Genitalia mounted.

*Liogenys seabrai* male holotype (MACN): [white handwritten] “Ene-957/BRASIL/Rio Janeiro/D.F. TIJUCA/C. A. C. Seabra/ A. Martínez- coll.”, [red printed] HOLOTYPUS, [red handwritten] “Liogenys/seabrai (M)/ sp. N./ [printed] A. MARTÍNEZ- DET. 1957”.

##### Non-type material.

BRAZIL. RJ: Manguinhos, 22/IX/1913, R. Fischer Col., 2 ex.; 28/IX/1916, R. Fischer col., 1 ex.; 19/X/1917, R. Fischer col., 1 ex.; 22/X/1917, R. Fischer col., 2 ex. (SDEI); Tijuca, I/1957, C. A. C. Seabra and A. Martinez col., 1 ex. (MACN); Seabra (CMNC); 4/XI/1957, A. Martinez col. (MNRJ); II/1957, M. Alvarenga col., 1 ex. (DZUP); Corcovado, 5/XI/1937; Friedr. Tippmann Wien col, 1 ex. (ZMHB); 15/IX/1961, J. S. Moure col. 1 ex. (DZUP); IX/1961, IX/1961, C. A. Seabra and M. Alvarenga (DZUP); Galeão, XI/1953, M. Alvarenga, 1 ex. (DZUP); Praia Brava, V/1975, Jesus col., 1 ex. (CEIOC); Represa Amorim, I/1933, Travassos col., 1 ex. (CEIOC); Jacarepagua, I/1957, A. Martinez col., 1ex. (CMNC).

##### Diagnosis.

**Figure 76.**
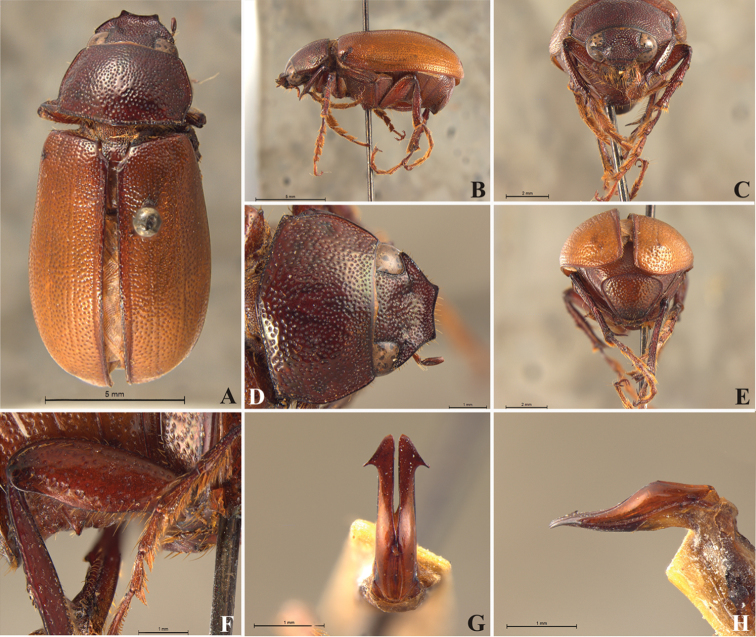
*Liogenys testaceipennis* Moser **A** Dorsal view **B** Lateral view **C** Frontal view **D** Clypeus and pronotum **E** Pygidium **F** Abdomen, lateral view **G** Parameres, dorsal view **H** Parameres, lateral view.

Body brownish; elongate; elytra testaceous to brownish, pronotum darker, reddish brown in males and dark brown in females; clypeal emargination rounded and wide; outer sides of anterior teeth sub-parallel; clypeal lateral margin convex in males, straight in females; male mesotibia quadrate in cross section, sub-quadrate to cylindrical in females; pygidium varies from flat to convex, as wide as it is long; pygidial disc bristled only on apex. In males, metafemur medially produced on posterior margin; inner margin of metatibia medially produced and ventrite IV medially produced; parameres of genitalia near three times the length of their apex; inner margins straight; apex harpoon-like, lateral angle curved projecting almost perpendicular to parameres (Fig. 76G).

##### Redescription.

Length: 13.0–13.8 mm; width: 6.3–6.7 mm. Testaceous to brownish. *Head*: distance between eyes nearly twice the width of one eye; frons equal in length to clypeus; clypeal emargination rounded, shallow and wide; outer sides of anterior teeth sub-parallel; outer margin of anterior teeth shorter than the eye; clypeal lateral margin concave in males, straight in females; distal maxillary palpomere, maximum width less than twice width of apex; fovea shallow, extending past the transverse midline of the palpomere; labium transversely carinated, as wide as it is long; antenna 10-articulated, lamellae lighter in color and longer than flagellum. *Thorax*: anterior margin of pronotum slightly produced medially; maximum length of pronotum exceeding the length of tarsomeres I, II and III together; disc glabrous, punctures fine and sparse; pronotal posterior corners sharp, almost right-angled; proepisternum with short bristles; mesepisternum scaly; sides of metasternum scaly and bristled, few long bristles on the anterior margin; distance between meso- and metacoxae up to twice longer than the metacoxa; scutellum ogival, coarsely punctured at the sides. *Elytra*: shiny, glabrous, uniform testaceous, lighter in color than the pronotum; elytra more than three times longer than the pronotum; elytral suture slightly darker than elytron and distinctly elevated; two pairs of inner ridges more noticeable than the two outer pairs. *Legs*: procoxa bristled on infra-carinal and outer surface; punctures visible at 12× magnification; three protibial teeth, middle and apical equal in size; the three teeth equally spaced; protibial inner apical spur present; mesofemur with a row of long bristles on anterior and posterior margins; male mesotibia quadrate in cross section, sub-quadrate to cylindrical in females; disc coarsely sculptured, metatibial apical transverse carina in males partial or complete, in females always complete; basal apophysis of metacoxa produced beyond the outer margin of trochanter; metatibia with posterior discontinuous longitudinal carina; metatibial apical spurs equal in length, length equal to the diameter of the tibial apex; male metafemur medially produced on posterior margin; inner margin of male metatibia carinated and medially produced towards apex, apical inner surface setose; metatibial disc finely sculptured; metatibial transverse carina present posteriorly; basal metatarsomere slightly wider and equal to or slightly longer than tarsomere II; in males, protarsomere II long; pro- and mesotarsomeres I to IV enlarged, protarsomeres slightly wider than the mesotarsomeres and more than twice as wide as metatarsi; claw bifid, symmetrical, superior tooth longer and as wide as the inferior; distance between teeth as long as the inferior tooth. *Abdomen*: ventrites bristled on disc; propygidium visible, glabrous; pygidium flat or convex, sub-trapezoidal, as wide as it is long; pygidial width not exceeding distance between spiracles of propygidium, pygidial disc bristled only on apex; ventrite IV in males medially produced; male pygidial apex quadrate. *Parameres*: width of basal region equal to the parameres together at its transverse midline, parameral split at the third portion; total length of parameres near three times the length of their apex; inner margins straight; apex harpoon-like with lateral angle projecting almost perpendicular to parameres (Fig. 76G). In lateral view parameres concave (Fig. 76H).

##### Type-locality.

*Liogenys testaceipennis*: BRAZIL; *Liogenys seabrai*. BRAZIL.Tijuca, RJ [Rio de Janeiro state]. (Syn.)

##### Geographical distribution.

BRAZIL (RJ).

##### Remarks.

*Liogenys testaceipennis* (Fig. 76F) differs from *L. spiniventris* (Fig. 73F) in the spine-like projection only on ventrite IV in males; the clypeal emargination rounded, clypeal lateral margin concave and not produced and pronotal punctures more sparsely distributed. *Liogenys testaceipennis* (ZMHB) and *L. seabrai* (MACN) primary types were studied and we concluded that they are conspecific, so herein *L. seabrai* is designated **junior subjective synonym** of *L. testaceipennis*.

#### Liogenys tibialis

Taxon classificationAnimaliaColeopteraMelolonthidae

Moser, 1918

Figs 77 , 90

Liogenys tibialis Moser, 1918: 107 (orig. desc.); Blackwelder 1944: 228 (check.); Frey 1969: 40 (junior syn. of L. palmata). **Stat. Rest.**

##### Type material.

*Liogenys tibialis* male syntype (ZMHB): [white printed] “Brasilia/ [handwritten] Theresopolis”, [white handwritten] “Liogenys/tibialis Mos/Typen”, [red printed] “Typus”, [white printed] “CUM TYPO/COMPARATUM”, [white handwritten] “Liogenys/palmatus/Burm/[printed] det. G. Frey 1968”. Genitalia mounted. As it is the unique primary type, it is considered the holotype.

##### Non-type material.

BRAZIL. MG: Passa Quatro, 19/IX/1922, 915 m, J. Zikan col., 1 ex.(CEIOC); RJ: Bomsucesso, VIII/1922, Barros col., 1 ex. (CEIOC); Petrópolis, Mosela, 24/I/1956, D’Albuquerque col., 1 ex. (MNRJ); Itatiaia, XI/1947, 900 m, Nick col., 1 ex. (CMNC); 1/I/1927, Ohaus col., 2 ex. (SDEI); I/1968, Dirings col., 1 ex. (MZSP); 1/X/1926, 700 m, J. Zikan col., 1 ex.; 9/I/1929, 700 m, J. Zikan col., 1 ex.; 20/IX/1929, 700 m, J. Zikan col., 1 ex.; 14/X/1937, W. Zikan col., 1 ex. (CEIOC); X/1962, 900 m, L. Almeida and O. Mielke cols., 1 ex. (MNRJ); 18/IX/1935, without collector, 1 ex.; 19/X/1944, 700 m, without collector, 2 ex.; XI/1947, 700 m, without collector, 1 ex. (MNRJ); “Fazenda Penedo”, 19/XI/1942, Rygod col., 1 ex. (MNRJ); “Lago azul”, 19/VI/1954, Daicy, Barros, Pearson cols., 4 ex. (MNRJ); 1 ex. (ZMHB); Theresopolis, without date and collector, 1 ex. (ZMHB); “Soberbo”, 15/X/1978, Becker, 1 ex. (MCNZ); SP: without date, M. Melzer col., 1 ex.; without date, J Metz col., 1 ex. (SDEI); São Paulo, without date and collector, 3 ex. (MNRJ); 21/IX/1914, without collector, 1 ex. (CEIOC); Alto da Serra, without date and collector, 1 ex. (DZUP); without date and collector, 1 ex. (ZMHB); “Paranapiacaba”, 30/III/1924, R. Spitz col., 1 ex. (MNRJ); I/1928, without collector, 1 ex. (MZSP); without date and collector, 1 ex. (CMNC); Ipiranga, without date and collector, 1 ex. (MZSP); 12/V/1983, without collector, 1 ex. (CMNC); 29/IX/1926, Ohaus col., 1 ex.; 15/X/1926, Ohaus col., 1 ex. (SDEI); 24/IX/1926, Ohaus col., 1 ex. (NHMB); 23/IX/1937, Lange de Morretes col., 1 ex. (MZSP); Santo Amaro, XII/1958, J. Lane, 2 ex.; X/1958, J. Lane, 1 ex. (IBSP); Salesópolis, Boracéia, 17/X/1960, Lenko col. 1 ex. (MZSP); Campos do Jordão, “Parque Estadual Campos do Jordão”, 15/X/1992, Exp. MZSP col., 1 ex. (MZSP); PR: Curitiba, XI/1938, without collector, 1 ex. (IBSP); Piraquara, Manancial da serra do Mar, 25°29'46"S, 48°58'54"W, 16/XII/2006, 1000 m, Rafael and Melo cols., 1 ex. (INPA); Campo Largo/ Estr. de Cerne Km 45, 22/XI/1979, without collector, 1 ex.; Campo tenente, 10/X/1973, Buzzi col., 1 ex.; Morretes, “Marumbi”, 14/I/1967, 500 m, Laroca and Nigro cols., 1 ex.; 15-16/X/1966, 500 m, Mielke and Laroca cols., 2 ex.; 6/X/1967, 500 m, Laroca and Giacomel cols., 1 ex.; 14/II/1967, 500 m, without collector, 1 ex.; 16-17/I/1970, Laroca and Becker cols., 1 ex. (DZUP); Ponta Grossa, XI/1954, without collector, 1 ex.; X/1957, without collector, 1 ex. (DZUP); Guaratuba, Estr. Dos Castelhanos, 9/IX/2007, JA Rafael and P Grossi cols., 6 ex. (EPGC); SC: Rancho Queimado, 15-18/XI/1995, Bonaldo col., 1 ex. (MCNZ), Hansa Humboldt [Corupá], III/1954, 60 m, without collector, 1 ex. (DZUP).

##### Diagnosis.

**Figure 77.**
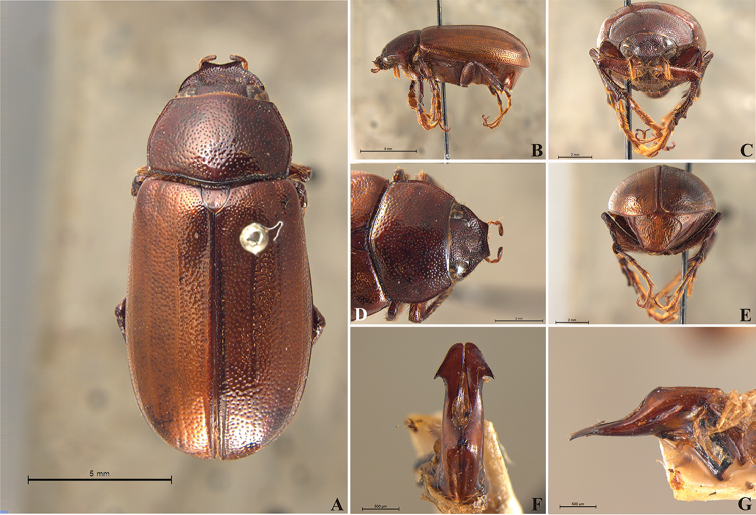
*Liogenys tibialis* Moser. **A** Dorsal view **B** Lateral view **C** Frontal view **D** Clypeus and pronotum **E** Pygidium **F** Parameres, dorsal view **G** Parameres, lateral view.

Body brownish; elongate; elytra testaceous to brownish, pronotum darker, reddish brown in males and dark brown in females; clypeal emargination sub-angled and wide; outer sides of anterior teeth sub-parallel; clypeal lateral margin slightly convex in males and straight in females; male mesotibia quadrate in cross section, sub-quadrate to cylindrical in females; male metafemur medially produced on posterior margin; inner margin of male metatibia medially produced towards apex; pygidium varies from flat to convex, as wide as it is long; pygidial disc bristled only on apex; male ventrites slightly elevated along the midline from ventrite I to V; genitalia, parameral split on third portion; total length of parameres near three times the length of their apex; inner margins straight or slightly convergent; apex harpoon-like with lateral angle projecting straight downward (Fig. 77F).

##### Redescription.

Length: 13.5–14.5 mm; width: 6.4–7.3 mm. Brownish. *Head*: distance between eyes nearly twice the width of one eye; frons equal in length to clypeus; clypeal emargination sub-angled, shallow and wide; outer sides of anterior teeth sub-parallel; outer margin of anterior tooth shorter than the eye; clypeal lateral margin slightly convex in males and straight in females; canthus not exceeding the outer margin of the eye; distal maxillary palpomere, maximum width less than twice the width of apex; fovea shallow, extending past the transverse midline of the palpomere; labium transversely carinated, as wide as it is long; antenna 10-articulated, lamellae lighter in color and longer than flagellum. *Thorax*: anterior margin of pronotum slightly produced medially; maximum length of pronotum exceeding the length of tarsomeres I, II and III together; disc glabrous, punctures sparse and coarse; pronotal posterior corners sharp, almost right-angled; proepisternum with short bristles; mesepisternum scaly; sides of metasternum scaly and bristled, few long bristles on the anterior margin; distance between meso- and metacoxae up to twice longer than the metacoxa; scutellum ogival, finely punctured at the sides. *Elytra*: shiny, glabrous, slightly pruinose in females; uniform brownish to testaceous, lighter in color than pronotum; elytra more than three times longer than the pronotum; elytral suture slightly darker than elytron and distinctly elevated; two pairs of inner ridges more noticeable than the two outer pairs. *Legs*: procoxa scaly on infra-carinal and outer surface; punctures visible at 12× magnification; three protibial teeth, middle and apical equal in size, the three teeth equally spaced; protibial inner apical spur present; mesofemural disc setose, with a row of long bristles on anterior and posterior margins; mesotibia quadrate in cross section in males, sub-quadrate to cylindrical in females; disc coarsely sculptured, mesotibial apical transverse carina in males partial or complete, in females always complete; basal apophysis of metacoxa produced beyond the outer margin of trochanter; male metafemur medially produced on posterior margin; inner margin of male metatibia carinated and medially produced towards apex, apical inner surface setose; metatibial disc finely sculptured; two metatibial transverse carinae present posteriorly and posterior discontinuous longitudinal carina; metatibial apical spurs equal in length, length equal to the diameter of the tibial apex, protarsomere II long; basal metatarsomere equal to tarsomere II in length and width; in males pro- and mesotarsomeres I to IV enlarged, protarsomeres slightly wider than the mesotarsomeres and more than twice as wide as metatarsi; claw bifid, symmetrical, superior tooth longer and as wide as the inferior; distance between teeth shorter than the inferior tooth. *Abdomen*: ventrites bristled on disc, in males ventrite I to V slightly elevated along the midline; propygidium visible, glabrous; pygidium flat or convex, sub-trapezoidal, as wide as it is long; pygidial width not exceeding distance between spiracles of propygidium, pygidial disc bristled only on apex; pygidial apex in males quadrate. *Parameres*: width of basal region equal to the parameres together at its transverse midline, parameral split at the third portion; total length of parameres near three times the length of their apex; inner margins straight or slightly convergent; apex harpoon-like with lateral angle projecting straight downward (Fig. 77F). In lateral view, parameres strongly concave (Fig. 77G).

##### Type-locality.

BRAZIL. Teresópolis, Rio de Janeiro.

##### Geographical distribution.

BRAZIL (**MG**, RJ, **SP**, **PR**, **SC**, RS).

##### Remarks.

*Liogenys tibialis* resembles *L. punctaticollis* (Fig. 69A), *L. spiniventris* (Fig. 73A) and *L. testaceipennis* (Fig. 76A) in color, size, elongate body, sides almost parallel and metatibiae flattened, among other several characters that relate them, as seen in Cherman et al. (2016). The most closely related species of *L. tibialis* is *L. punctaticollis* which also show ventrites not furnished with projections and, thus, they both differ from the other two species of the clade. Frey (1969) synonymized *Liogenys tibialis* with *L. palmata* and later the same author (Frey 1974) synonymized *L. palmata* with *L. punctaticollis*, being the last one the senior synonym of the other two. After studying the primary types of the all three species, we verified that *L. punctaticollis* and *L. palmata* are indeed conspecific, but it differs in that *L. tibialis* has the clypeal lateral margin slightly convex in males, metatibial disc more coarsely sculptured; the elytra more shiny in females and parameres are also distinctive. *Liogenys tibialis* parameres are near three times the length of their apex, and the apex has the lateral angle projecting straight downward. Instead, *Liogenys punctaticollis* parameres are near five times the length of their apex, inner margins more separated and apex with lateral angle curved projecting almost perpendicular to parameres. Also, in males of *L. tibialis* the abdomen, although not produced, it is slightly elevated along the midline from ventrite I to V. We concluded that *L. tibialis* is a valid name. When Frey (1969) synonymized *L. tibialis* with *L. palmata*, he put an additional label under *L. tibialis* primary type: “CUM TYPO/COMPARATUM”. As this type specimen bears the labels written by Moser (1918), including the type-locality “Theresópolis” and Moser described the species based only on one specimen, this one is considered the holotype.

#### Liogenys unicolor

Taxon classificationAnimaliaColeopteraMelolonthidae

Evans, 2003

Figs 78 , 93

Hilarianus concolor Blanchard, 1851: 170 (orig. desc.); Lacordaire 1856: 270 (genus red.); Harold 1869a: 1141 (check.); Dalla Torre 1913: 319 (check.); Blackwelder 1944: 228 (check.).
Liogenys concolor : Frey 1974: 331 (not Blanchard 1851: 167) (n. comb.).
Liogenys unicolor Evans, 2003: 215 (replacement name); Evans and Smith 2005: 178 (check.); Evans and Smith 2009: 183 (check.)

##### Type material.

*Hilarianus concolor* syntype (MNHN): [white handwritten] “Capit^e^/des Mines”, [light green printed] “MUSÉUM PÁRIS/ [handwritten] Caple/des/Mines”, [red printed] “SYNTYPE”, [green handwritten] “H. concolor/ Cat Mus/ Brésil/ M. A. S^t^ Hilaire”. This type is here designated the **lectotype** [white, outlined in red, printed] “LECTOTYPE/*Hilarianus concolor*/Blanchard, 1851/ des. M. A. Cherman 2014”.

##### Non-type material.

BRAZIL. MG: Cruzeiro, EPDA-Peti, 13/X/1998, Vasconcellos col., 1 ex. (CEMT); MT: 1948, Kolug and Carvalho, 1 ex. (MNRJ); without locality, date and collector, 1 ex. (ZMHB).

##### Diagnosis.

**Figure 78.**
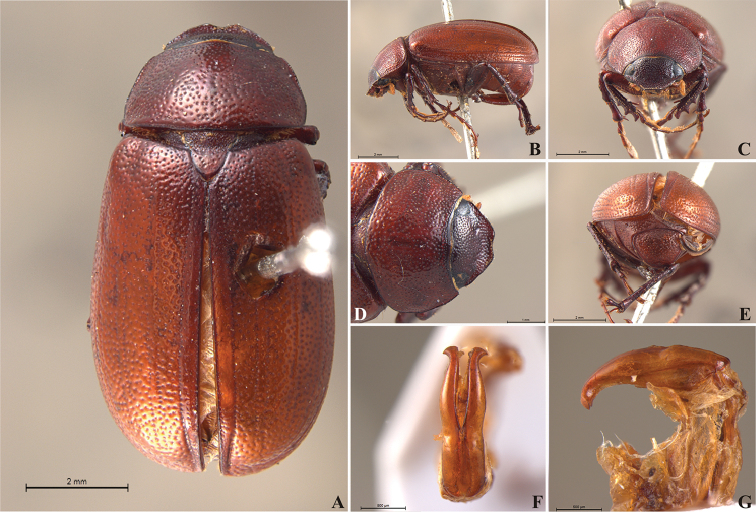
*Liogenys unicolor* Evans. **A** Dorsal view **B** Lateral view **C** Frontal view **D** Clypeus and pronotum **E** Pygidium **F** Parameres, dorsal view **G** Parameres, lateral view.

Body reddish brown; elongate, slightly wider on the posterior third; elytra uniform reddish brown as the pronotum; frons swollen, longer than clypeus; clypeus weakly emarginate; outer sides of anterior teeth follow the lateral margin of clypeus; canthus exceeding the outer margin of the eye; sensorial area not forming a fovea; pronotal maximum length equal to tarsomeres I, II and III together; anterior margin of pronotum apparently concave, pronotal anterior corners bent frontwards; basal apophysis of metacoxa not produced; inner margin of metatibia not carinated; in males pro- and mesotarsomeres I to IV weakly enlarged; pygidium convex, glabrous; parameres narrowed at the transverse midline and subapically; divergent; apex truncated and strongly curved downwards (Fig. 78F).

##### Redescription.

Length: 9.0–9.4 mm; width 5.0–5.1 mm. Reddish brown. *Head*: distance between eyes nearly three times wider than one eye; frons somewhat swollen, longer than clypeus; clypeal emargination weak, sub-angled, shallow and narrow; outer sides of anterior teeth follow the lateral margin of clypeus; lateral margin straight; canthus exceeding the outer margin of the eye; distal maxillary palpomere, maximum width less than twice width of apex; sensorial area not reaching the transverse midline of the palpomere and not forming a fovea; labium transversely carinated, as wide as it is long; antenna 10-articulated, lamellae lighter in color than flagellum and equal in length. *Thorax*: anterior margin of pronotum slightly produced medially; maximum length of pronotum equal to tarsomeres I, II and III together; disc glabrous, punctures very sparse; pronotal anterior corners bent frontwards; pronotal posterior corners rounded; pronotal convexity on lateral margins weak; proepisternum with short bristles; mesepisternum scaly; sides of metasternum with sparse long and short bristles; distance between meso- and metacoxae up to twice longer than the metacoxa; scutellum ogival, punctured only at the base. *Elytra*: shiny, glabrous, uniform reddish brown; elytra more than three times longer than the pronotum; elytral suture and elytron unicolored, distinctly elevated; four elytral ridges barely noticeable, the outer one slightly more noticeable than the others. *Legs*: procoxa, sparse scales on infra-carinal and outer surface; punctures visible at 12× magnification; three protibial teeth, middle and apical equal in size, the three teeth equally spaced; protibial inner apical spur present; mesofemural disc smooth, with a row of short bristles on the anterior and posterior margins; mesotibia cylindrical in cross section; disc finely sculptured; two mesotibial transverse carinae, the apical one complete; basal apophysis of metacoxa not produced; inner margin of metatibia not carinated or produced on apex; inner surface setose; metatibial disc coarsely sculptured; a complete metatibial transverse carina present posteriorly; metatibial apical spurs of different lengths, the longest equal in length to the diameter of the tibial apex; pro- and mesotarsomeres I to IV weakly enlarged, protarsomeres as wide as the mesotarsomeres, slightly wider than the metatarsi; basal metatarsomere up to one-half the length of tarsomere II and wider; claw bifid, symmetrical, superior tooth longer and as wide as the inferior; distance between teeth longer than the inferior tooth. *Abdomen*: Ventrites bristled on disc and scaly on sides; propygidium slightly visible, pygidium convex, sub-trapezoidal, wide; pygidial width not exceeding distance between spiracles of propygidium; pygidial disc glabrous; pygidial apex rounded. *Parameres*: width of basal region equal to the parameres together at its maximum width, parameral split at the third portion almost the midline, narrowed at this point and also subapically; inner margins of parameres divergent; apex truncated (Fig. 78F). In lateral view parameres convex, apex strongly curved downwards (Fig. 78G).

##### Type locality.

BRAZIL. “Capit.e des Mines” [Minas Gerais state].

##### Geographical distribution.

BRAZIL (**MT**, MG).

##### Remarks.

*Liogenys unicolor* resembles *L. macropelma* and shares with this species less frequent *Liogenys* characters, as the sensorial area of the maxillar distal palpomere flat, not forming a fovea; pronotum as long as protarsomeres I, II e III together, and pro- and mesotarsi weakly enlarged in males. Despite this, these species are not closely related according to Cherman et al. (2016) and instead, it is more closely to *Liogenys sinuaticeps*, which, in addition to the flat palpomere of the maxilla, shares features as the basal apophysis of metacoxa not produced and the pygidium glabrous. *Liogenys unicolor* holotype has 10-articulated antenna and a weakly but still emarginate clypeus, disagreeing with Blanchard (1851) who described the species with a 9-articulated antenna and rounded clypeus instead. Females remain unknown.

#### Liogenys cavifrons

Taxon classificationAnimaliaColeopteraMelolonthidae

Cherman sp. n.

http://zoobank.org/1CB6159E-7B64-48EE-BDBB-60BB25B485D9

Figs 79 , 89

##### Type-specimen.

Holotype male, pinned, with genitalia mounted. Original labels: [white printed] “Coleção/ M. Alvarenga”, [White, outlined, printed] “Mangabeira [Manjeiro]/MOCAJUBA PARA/BRASIL VII-1953/Orlando Rego”, [red printed] “HOLOTYPE”, [white printed] “DZUP/401710”. Paratypes (11) bearing the label [red printed] “PARATYPE”: Male paratype with the same data of the holotype [white printed] “DZUP/401711” (DZUP). Female paratype with the following data: [white printed] “CONCEIÇÃO ARAGUAIA/Pará Brasil VII-1959/M. Alvarenga”, [white printed] “DZUP/401712” (DZUP). Male and female paratypes with the following data: [white printed] “COLEÇÃO/CAMPOS SEABRA”, [White, outlined, printed] “Mangabeira [Manjeiro]/MOCAJUBA PARA/BRASIL VII-1953/Orlando Rego” (MNRJ). One male and two female paratypes with the following data: [white printed] “COLEÇÃO/CAMPOS SEABRA”, [White, outlined, printed] “Mangabeira [Manjeiro]/MOCAJUBA PARA/BRASIL VIII-1953/O. Rego” (MNRJ). Male paratype with the following data: [White printed] “COLEÇÃO/CAMPOS SEABRA”, [White, outlined, printed] “Mangabeira [Manjeiro]/MOCAJUBA PARA/BRASIL X-1953/O. Rego” (MNRJ). Female paratype with the following data: [white printed] “Cachimbo, Estado do Pará/Alt. 400 m., 14/21 - IX - 955/ L. Travassos and S. Oliveira col.” (CMNC). Male paratype with the following data: [white printed] “BRASIL: (MA), Mirador/Parque Est. Mirador/Base da Geraldina”, [White printed]“Armadilha Luminosa/21 - 25.viii.2006, F./Limeira-de-Oliveira”, [White printed]“154”, [White printed]“CZMT-CEMT/0000018031” (CEMT). Female paratype with the following data: [White printed] Distrito Federal/Planaltina. Embrapa Cerrados./Cerrado Nativo 15°36'16"S 47°44'16"W. 07-X-2005. light./C. Oliveira”, [White printed]“CZMT-CEMT/0000018030” (CEMT).

Holotype and two paratypes deposited at DZUP, Universidade Federal do Paraná, Curitiba. The other paratypes are deposited at the following institutions: CEMT, Setor de Entomologia da Coleção Zoológica, Universidade Federal de Mato Grosso, Cuiabá; CMNC, Canadian Nature Museum, Ontario; DZUP, Coleção Entomologica Pe. Jesus Santiago Moure, Universidade Federal do Parana, Curitiba and MNRJ, Museu Nacional do Rio de Janeiro, Rio de Janeiro.

##### Diagnosis.

**Figure 79.**
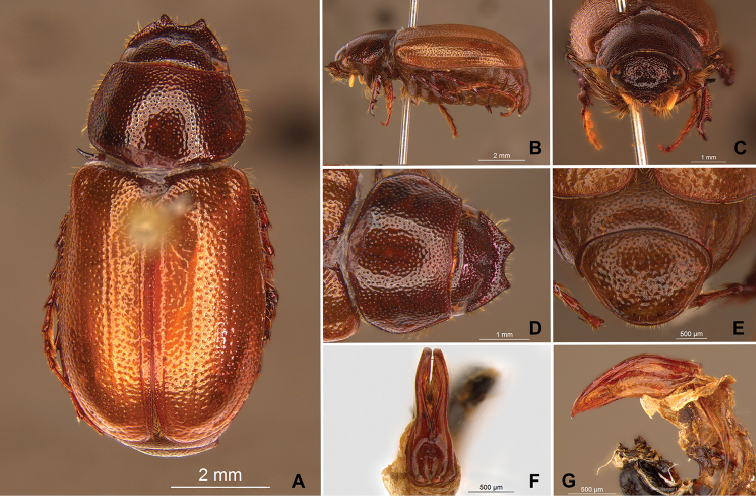
*Liogenys cavifrons* Cherman, sp. n. **A** Dorsal view **B** Lateral view **C** Frontal view **D** Clypeus and pronotum **E** Pygidium **F** Parameres, dorsal view **G** Parameres, lateral view.

Body brown; elongate; elytra yellowish brown, pronotum reddish brown; frons surface irregular, excavated medially; clypeal emargination deep, rounded and wide; outer sides of anterior teeth sub-parallel; pronotal posterior corners obsolete; mesotibia cylindrical in cross section; inner margin of metatibia not carinated on apex and inner surface glabrous; pygidium convex, sub-trapezoidal; pygidial width exceeding distance between spiracles of propygidium; in males pro- and mesotarsi weakly enlarged; male genitalia, basal region wider than the parameres together at its maximum width, parameral split at the third portion; parameral apex fusiform (Fig. 79F).

##### Holotype.

**Male.** Length: 8.8 mm; width: 4.2 mm. Yellowish brown. *Head*: distance between eyes nearly twice the width of one eye; frons swollen and excavated medially, longer than clypeus; clypeal emargination deep, rounded and wide; outer sides of anterior teeth sub-parallel; outer margin of anterior teeth less than one-half the length of the eye; clypeal lateral margin convex; canthus not exceeding the outer margin of the eye; distal maxillary palpomere, maximum width more than twice width of apex; fovea deep extending past the transverse midline of the palpomere; labium transversely carinated, as wide as it is long; antenna 10-articulated, lamellae lighter in color than the flagellum and equal in length. *Thorax*: anterior margin of pronotum slightly produced medially; maximum length of pronotum exceeding the length of tarsomeres I, II and III together, disc glabrous, punctures fine and sparse; pronotal posterior corners obsolete; proepisternum with long bristles; mesepisternum sparsely scaly; sides of metasternum with short bristles and few long ones on the anterior margin; distance between meso- and metacoxae up to twice the length of the metacoxa; scutellum ogival, finely punctured at the sides. *Elytra*: shiny, glabrous, uniform yellowish to testaceous, lighter in color than pronotum; elytra more than three times longer than the pronotum; elytral suture and elytron unicolored and scarcely elevated; all four elytral ridges barely noticeable. *Legs*: procoxa bristled and scarcely scaly on infra-carinal surface; punctures visible at 12× magnification; three protibial teeth, the apical the longest, distance between basal and middle teeth slightly shorter than between middle and apical; protibial inner apical spur present; mesofemural disc setose, abundant long bristles on the anterior margin; mesotibia cylindrical in cross section; disc finely sculptured, two mesotibial transverse carinae, the apical one complete; metacoxa with long bristles at the sides, basal apophysis of metacoxa produced beyond the outer margin of trochanter; inner margin of metatibia carinated excepting the apex; inner surface glabrous; metatibial disc finely sculptured; two metatibial transverse carinae present posteriorly; metatibial apical spurs equally long, exceeding the diameter of the tibial apex; protarsomere II long; protarsomeres I to IV slightly enlarged; basal metatarsomere and tarsomere II equal in size; claw bifid, symmetrical, superior tooth longer and narrower than the inferior; distance between teeth shorter than the inferior tooth. *Abdomen*: ventrites scarcely bristled on disc; propygidium glabrous; pygidium convex, sub-trapezoidal, wide; pygidial width exceeding distance between spiracles of propygidium; pygidial disc bristled only on apex; pygidial apex sub-rounded. *Parameres*: basal region wider than the parameres together at its maximum width, parameral split at the third portion; inner margins convergent; parameres apex fusiform (Fig. 79F). In lateral view parameres convex, strongly curved downwards (Fig. 79G).

**Female** paratype. Length: 8.5 mm; width: 4.5 mm. As the holotype except in the clypeal teeth that follow the lateral margin of clypeus and the clypeal lateral margin straight or slightly convex; metasternum scarcely scaly; scutellum punctures randomly distributed; pygidium flatter, longer or as wide as it is long and apex more angled. **Variation**. Male paratypes. Length: 8.8–8.9 mm; width: 4.2–4.3 mm. As the holotype except in the scutellum triangular to rounded and with punctures randomly distributed.

##### Etymology.

Adjective in the nominative singular. New Latin; from *cavus* (“hollow, sunken”) + *frōns* (“forehead, front”). The species name is due to the frons excavated medially, what distinguishes this species from the all other *Liogenys*.

##### Type-locality.

BRAZIL, Pará: Mocajuba, Mangabeira [Manjeiro] [49°30'00"W; 2°33'60"S], August 1953, Orlando Rego Col..

##### Geographical distribution.

BRAZIL (PA, MA, DF).

##### Remarks.

*Liogenys cavifrons* Cherman, sp. n. resembles *L. corumbana* (Fig. 61) in the size and shape of the body and differs from it by the pronotum and elytra slightly darker, clypeus not produced laterally and tarsi in males slightly enlarged. This species also resembles *L. acutidens* in color, differing from it in being smaller, having the clypeus not produced laterally, the elytral suture wider and the same color as the elytra; the scutellum more punctured; the pygidium glabrous and the tarsi in males slightly enlarged. The dimorphic features found commonly in *Liogenys* males as the abdomen ventrally concave; metatibia strongly carinated along the inner margin with inner surface bristled and pro- and mesotarsi enlarged, are absent or indistinct in *L. cavifrons* Cherman, sp. n.

#### Liogenys femella

Taxon classificationAnimaliaColeopteraMelolonthidae

Cherman sp. n.

http://zoobank.org/D3E291C0-7440-4775-BA9F-653724D77F14

Figs 80 , 93

##### Type-specimen.

Holotype female, pinned. Original label: [white, outlined black] “[handwritten] PIRAPORA/[printed]Minas Gerais BRASIL/[handwritten]1954”, [red printed] “HOLOTYPE” (MNRJ). Female paratype (1) with the same data of the holotype plus the label [red printed] “PARATYPE” (MNRJ). Holotype and paratype deposited at MNRJ, Museu Nacional do Rio de Janeiro, Rio de Janeiro.

##### Diagnosis.

Body brown; elongate; elytra light brown, pronotum reddish brown; clypeus quadridentate due to the tooth-like projection of the lateral margin; clypeal emargination deep and angled; outer sides of anterior teeth parallel; anterior margin of pronotum depressed throughout; pronotal posterior corners rounded; mesotibia sub-quadrate in cross section; inner margin of metatibia not carinated on apex and inner surface glabrous; pygidium slightly convex, sub-trapezoidal; glabrous, bristled only at the apical margin. No males known until now for this species

##### Holotype.

**Figure 80.**
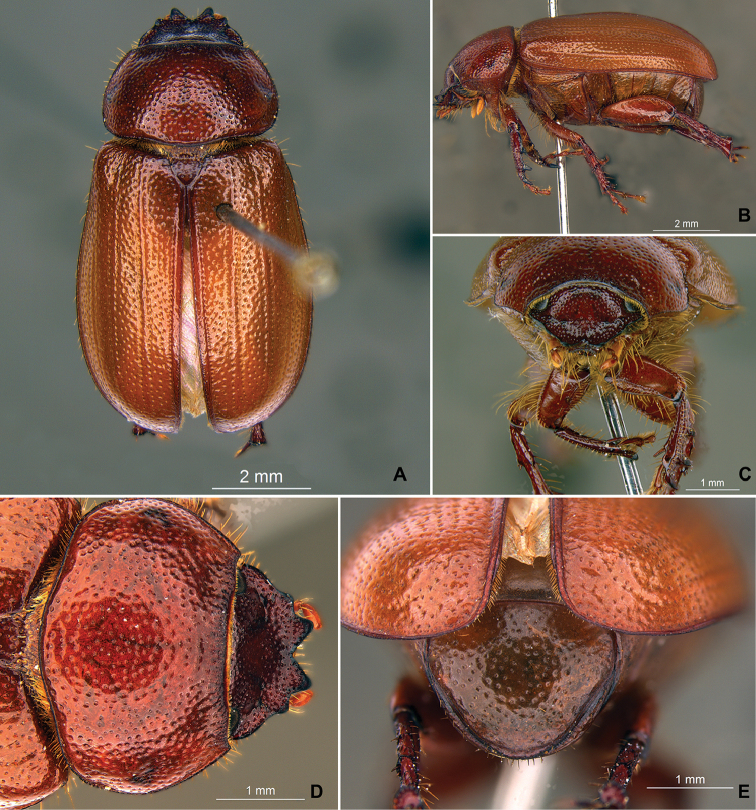
*Liogenys femella* Cherman, sp. n. **A** Dorsal view **B** Lateral view **C** Frontal view **D** Clypeus and pronotum **E** Pygidium.

**Female**. Length: 8.6 mm; width: 4.7 mm. Brownish. *Head*: distance between eyes nearly twice the width of one eye; frons equal in length to clypeus; clypeal emargination deep, sharp and wide; outer sides of anterior teeth parallel; outer margin of anterior teeth shorter than the eye; clypeal lateral margin convex and strongly produced forming a tooth-like projection; distance between lateral and anterior tooth equal to basal width of anterior tooth, distance between lateral tooth and anterior margin of eye longer than one eye, right angle between anterior and lateral teeth; canthus not exceeding the outer margin of the eye; distal maxillary palpomere, maximum width less than twice the width of apex; fovea shallow extending up to the transverse midline of the palpomere; labium transversely carinated, as wide as it is long; antenna 10-articulated, lamellae lighter in color than the flagellum and equal in length. *Thorax*: anterior margin of pronotum straight and depressed throughout (Fig. 80D); maximum length of pronotum exceeding the length of tarsomeres I, II and III together, disc glabrous, punctures coarse and sparse; pronotal posterior corners rounded; proepisternum with long bristles; mesepisternum sparsely scaly; sides of metasternum with short bristles and few long ones on the anterior margin; distance between meso- and metacoxae up to twice the length of the metacoxa; scutellum ogival, coarsely punctured and scarcely bristled. *Elytra*: shiny, glabrous, light brown, lighter in color than pronotum; elytra more than three times longer than the pronotum; elytral suture slightly darker than elytron and elevated; two pairs of inner ridges more noticeable than the two outer pairs. *Legs*: procoxa bristled on infra-carinal and outer surface, smooth at 12× magnification; three protibial teeth, middle and apical equal in size, the three teeth equally spaced; protibial inner apical spur present; mesofemural disc glabrous, with a row of long bristles on the anterior and posterior margins; mesotibia sub-quadrate in cross section; disc finely sculptured; two mesotibial transverse carinae, the apical one incomplete; metacoxa with short bristles at the sides, basal apophysis of metacoxa produced beyond the outer margin of trochanter; inner margin of metatibia carinated excepting the apex; apical inner surface glabrous; metatibial disc coarsely sculptured; two metatibial transverse carinae present posteriorly, the basal one reduced; metatibial apical spurs of different lengths, the longest exceeding the diameter of the tibial apex; protarsomere II long; pro- and mesotarsomeres I to IV cylindrical; basal metatarsomere shorter and slightly wider than tarsomere II; claw bifid, symmetrical, superior tooth longer and narrower than the inferior; distance between teeth shorter than the inferior tooth. *Abdomen*: ventrites scarcely bristled on disc; propygidium slightly visible, glabrous; pygidium slightly convex, sub-trapezoidal, as wide as it is long; pygidial width exceeding distance between spiracles of propygidium; pygidial disc glabrous, bristled only at the margin of the apex; pygidial apex rounded. Males remain unknown. **Variation: Female** paratype. Length: 8.6 mm; width: 4.7 mm. As the holotype except in the apical transverse carina complete in metatibia.

##### Etymology.

Noun in the nominative singular. From Latin *femella* (“girl”). The species name is due to the female type material, as some features mentioned in the description might be present only in females.

##### Type-locality.

BRAZIL, Minas Gerais: Pirapora [44°56'30"W; 17°20'42"S], 1954.

##### Geographical distribution.

BRAZIL (MG).

##### Remarks.

*Liogenys femella* Cherman, sp. n. resembles *L. parva* in the size and body shape, and in the quadridentate clypeus. They differ in the scutellum, more punctured and bristled in *L. femella*.

#### Liogenys piauiensis

Taxon classificationAnimaliaColeopteraMelolonthidae

Cherman sp. n.

http://zoobank.org/37A0EC0F-6F86-40FE-A32C-033B3C79F048

Figs 81 , 89

##### Type-specimen.

Holotype male, pinned, with genitalia mounted. Original labels: [White, outlined, printed] “TERESINA/PIAUI BRASIL/[handwritten]1. 1953/[printed]A. K. OLIVEIRA”, [red printed] “HOLOTYPE” (MNRJ). Paratypes (4): Male paratype and three female paratypes with the same data of the holotype (MNRJ), plus the label: [red printed] “PARATYPE”. Holotype and four paratypes deposited at MNRJ, Museu Nacional do Rio de Janeiro, Rio de Janeiro.

##### Diagnosis.

**Figure 81.**
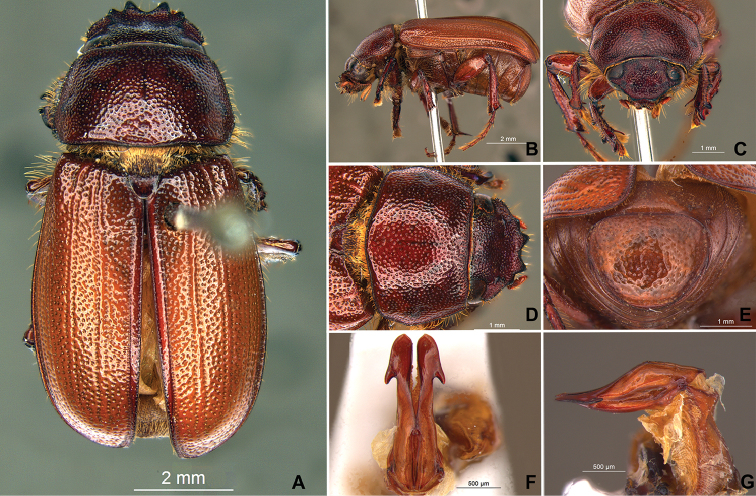
*Liogenys piauiensis* Cherman, sp. n. **A** Dorsal view **B** Lateral view **C** Frontal view **D** Clypeus and pronotum **E** Pygidium **F** Parameres, dorsal view **G** Parameres, lateral view.

Body brown; elongate; elytra brownish, pronotum slightly darker; clypeus quadridentate due to the tooth-like projection of the lateral margin; clypeal emargination deep, rounded; outer sides of anterior teeth parallel; clypeal lateral margin convex; anterior margin of pronotum depressed throughout; pronotal posterior corners sub-angled, obtuse (Fig. 81D); mesotibia sub-quadrate to cylindrical in cross section; pygidium convex, sub-quadrate; pygidial width exceeding distance between spiracles of propygidium; total length of parameres near three times the length of their apex; slightly narrowed medially; apex harpoon-like with lateral angle projecting straight downward (Fig. 81F).

##### Holotype.

**Male**. Length: 9.1 mm; width: 4.7 mm. Brownish. *Head*: distance between eyes nearly twice the width of one eye; frons equal to clypeus; clypeal emargination deep, rounded and wide; outer sides of anterior teeth parallel; outer margin of anterior teeth shorter than the eye; lateral margin convex and strongly produced forming a tooth-like projection; distance between lateral and anterior tooth equal to basal width of anterior tooth, distance between lateral tooth and anterior margin of eye longer than one eye, right angle between anterior and lateral teeth; canthus not exceeding the outer margin of the eye; distal maxillary palpomere, maximum width less than twice width of apex; fovea shallow extending up to the transverse midline of the palpomere; labium transversely carinated, as wide as it is long; antenna 10-articulated, lamellae lighter in color and longer than the flagellum. *Thorax*: anterior margin of pronotum straight and depressed throughout (Fig. 81D); maximum length of pronotum exceeding the length of tarsomeres I, II and III together; disc glabrous, punctures coarse and sparse; pronotal posterior corners sub-angled, obtuse; proepisternum with long bristles; mesepisternum scarcely scaly; sides of metasternum with short bristles and few long ones on the anterior margin; distance between meso- and metacoxae up twice the length of the metacoxa; scutellum sub-rounded, disc finely punctured at the base. *Elytra*: shiny, glabrous, light brown, lighter in color than pronotum; elytra more than three times longer than the pronotum; elytral suture slightly darker than elytron and slightly elevated; the two inner pairs of ridges and the outer one more noticeable than the third. *Legs*: procoxa bristled on infra-carinal and outer surface, smooth at 12× magnification; three protibial teeth, the middle the longest; distance between basal and middle teeth longer than between middle and apical; protibial inner apical spur present; mesofemural disc glabrous, a row of long bristles on the anterior margin; mesotibia sub-quadrate in cross section; disc coarsely sculptured, two mesotibial transverse carinae, the apical incomplete; metacoxa, scarcely bristled and finely punctured on the anterior or posterior margins, smooth on the metacoxal disc; basal apophysis of metacoxa produced beyond the outer margin of trochanter; inner margin of metatibia carinated towards apex, apical inner surface setose; metatibial disc finely sculptured; metatibial transverse carina present posteriorly and with posterior discontinuous longitudinal carina; metatibial apical spurs of different lengths, the longest one equal to the diameter of the tibial apex; protarsomere II long; pro- and mesotarsomeres I to IV enlarged, protarsomeres slightly wider than the mesotarsomeres; less than two-fold wider than metatarsi; the basal metatarsomere shorter and slightly wider than tarsomere II; claw bifid, symmetrical, superior tooth longer and narrower than the inferior; distance between teeth shorter than the inferior tooth. *Abdomen*: ventrites with few sparse bristles on disc; propygidium hidden by the elytra; pygidium convex, sub-quadrate, as wide as it is long; pygidial width exceeding distance between spiracles of propygidium; pygidial disc glabrous, bristled at the margin of the apex; very shiny, wrinkled, coarsely punctured; pygidial apex in males sub-quadrate. *Parameres*: basal region wider than the parameres together at its transverse midline; parameral split at the third portion; total length of parameres near three times the length of their apex; slightly narrowed medially; inner margins of parameres convergent; apex harpoon-like with lateral angle projecting straight downward (Fig. 81F). In lateral view parameres concave (Fig. 81G).

**Female** paratype. Length: 9.8 mm; width: 5.0 mm. As the holotype except in the lamellae and flagellum equal in length; protibial teeth equally spaced; metatibial apical transverse carina complete. **Variation**. **Male** paratype. Length: 9.0 mm; width: 4.7 mm, As the holotype.

##### Etymology.

Adjective in the nominative singular. Piauí (Brazilian State) + Latin suffix -*ensis* (“of or from a place”).The species name is a toponym derived from the State of the type-locality, Piauí

##### Type-locality.

BRAZIL, Piauí: Teresina [42°48'07"W; 5°05'22"S], Jan 1953, A. K. Oliveira Col.

##### Geographical distribution.

BRAZIL (PI).

##### Remarks.

**Figure 82.**
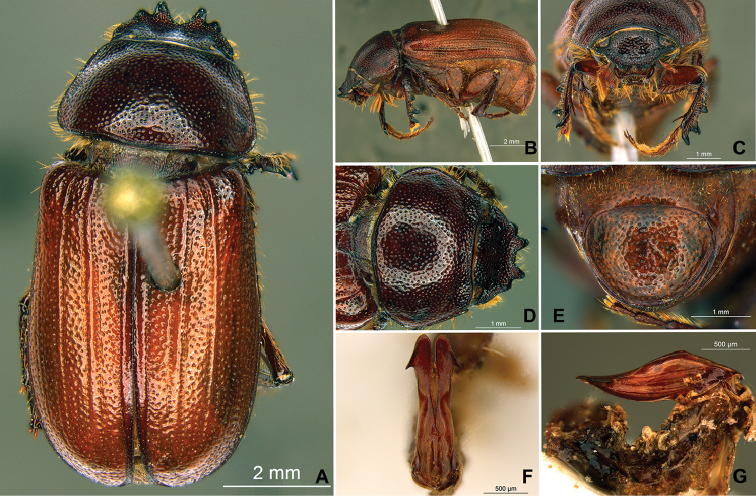
*Liogenys rotundicollis* Cherman, sp. n. **A** Dorsal view **B** Lateral view **C** Frontal view **D** Clypeus and pronotum **E** Pygidium **F** Parameres, dorsal view **G** Parameres, lateral view.

*Liogenys piauiensis* Cherman, sp. n. resembles *L. parva* in the size and shape of the body, as well as in the quadridentate clypeus. *Liogenys piauiensis* Cherman, sp. n. differs in the color of the elytra slightly darker, clypeal emargination slightly narrower; disc of metacoxa smooth (in common with *L. rotundicollis* Cherman, sp. n.) (Fig. 82), few punctures on metacoxal anterior margin and postero-external corner slightly produced; male genitalia with shape of parameres also distinctive, narrowed medially.

#### Liogenys rotundicollis

Taxon classificationAnimaliaColeopteraMelolonthidae

Cherman sp. n.

http://zoobank.org/D2E16FDA-CEE1-4A2B-B9E3-BD54A9EC4C92

Figs 82 , 89

##### Type-specimen.

Holotype male, pinned, with genitalia mounted. Original labels: [white printed] “BRASIL: PI: Canto do Buriti/18-22. xi. 1991. Amarante/Brandão Cancello & Ponte”, [red printed] “HOLOTYPE” (MZSP). Holotype deposited at MZSP, Museu de Zoologia da Universidade de São Paulo, São Paulo.

##### Diagnosis.

Body brown; elongate, sides parallel; elytra brown, pronotum darker; clypeus quadridentate due to the tooth-like projection of the lateral margin; clypeal emargination deep and rounded; outer sides of anterior teeth parallel; clypeal lateral margin convex; pronotal anterior margin depressed throughout; pronotal posterior corners obsolete; mesotibia sub-quadrate in cross section; metacoxal disc predominantly smooth, few punctures and bristles near the base of the femur; pygidium convex, wide; pygidial width exceeding distance between spiracles of propygidium; male genitalia, total length of parameres near five times the length of their apex; strongly narrowed medially; apex harpoon-like with lateral angle projecting straight downward (Fig. 82F).

##### Holotype.

**Male**. Length: 9.0 mm; width: 4.4 mm. Dark brown. *Head*: distance between eyes nearly twice the width of one eye; frons equal to clypeus; clypeal emargination deep, rounded and slightly wide, outer sides of anterior teeth parallel; outer margin of anterior teeth shorter than the eye; clypeal lateral margin convex and strongly produced forming a tooth-like projection; distance between lateral and anterior tooth equal to basal width of anterior tooth, distance between lateral tooth and anterior margin of eye longer than one eye, straight angle between anterior and lateral teeth; canthus not exceeding the outer margin of the eye; distal maxillary palpomere, maximum width nearly twice width of apex; fovea shallow extending up to the transverse midline of the palpomere; labium transversely carinated, as wide as it is long; antenna 10-articulated, lamellae lighter in color and longer than the flagellum. *Thorax*: anterior margin of pronotum straight and depressed throughout (Fig. 82D); maximum length of pronotum exceeding the length of tarsomeres I, II and III together; disc glabrous, punctures fine and sparse; pronotal posterior corners obsolete; proepisternum with long bristles; mesepisternum sparsely scaly; sides of metasternum with short and long bristles; distance between meso- and metacoxae up to twice the length of the metacoxa; scutellum ogival, finely punctured. *Elytra*: shiny, glabrous, brownish, slightly lighter in color than pronotum; elytra more than three times longer than the pronotum; elytral suture darker than elytron and elevated; the two inner pairs of ridges and the outer one more distinct than the third. *Legs*: procoxa bristled on infra-carinal and outer surface, smooth at 12× magnification; three protibial teeth, middle and apical equal in size; distance between basal and middle teeth slightly longer than between middle and apical; protibial inner apical spur present; mesofemural disc glabrous, bristled marginally, long bristles on the anterior margin and short in the posterior one; mesotibia sub-quadrate in cross section; disc coarsely sculptured, two mesotibial transverse carinae, the apical one incomplete; metacoxa glabrous, few punctures and bristles near the base of the femur; basal apophysis of metacoxa produced beyond the outer margin of trochanter; inner margin of metatibia carinated towards apex, apical inner surface setose; metatibial disc finely sculptured; metatibial transverse carina present posteriorly and posterior discontinuous longitudinal carina; metatibial apical spurs of different lengths, the longest one shorter than the diameter of the tibial apex; protarsomere II long; pro- and mesotarsomeres I to IV equally enlarged, less than twice as wide as metatarsi; basal metatarsomere slightly shorter than tarsomere II and equally wide; claw bifid, symmetrical, superior tooth longer and narrower than the inferior; distance between teeth shorter than the inferior tooth. *Abdomen*: ventrites scarcely bristled on disc; propygidium visible, bristled; pygidium convex, sub-quadrate, as wide as it is long; pygidial width exceeding distance between spiracles of propygidium; pygidial disc glabrous, bristled only on the margin of the apex, coarsely punctured, slightly depressed medially; pygidial apex sub-rounded. *Parameres*: basal region wider than the parameres together at its maximum width; parameral split at the third portion; total length of parameres near five times the length of their apex; strongly narrowed medially; inner margins of parameres straight; apex harpoon-like with lateral angle projecting straight downward (Fig. 82F). In lateral view parameres concave (Fig. 82G). Female remains unknown.

##### Etymology.

Adjective in the nominative singular. New Latin; from *rotundus* (“round”) + *collum* (“neck, stem”). The species name is due to the rounded appearance of the posterior margin of the pronotum, due to the posterior corners obsolete.

##### Type locality.

BRAZIL, Piauí: Canto do Buriti [42°56'39"W; 8°06'36"S], 18-22 Nov 1991, Amarante Brandão Cancello & Ponte Coll.

##### Geographical distribution.

BRAZIL (PI).

##### Remarks.

*Liogenys rotundicollis* Cherman, sp. n. resembles *L. parva* (Fig. 68A), *L. femella* Cherman, sp. n. (Fig. 80A) and *L. piauiensis* Cherman, sp. n. (Fig. 81A) in the size and shape of the body, as well as in the quadridentate clypeus. *Liogenys rotundicollis* Cherman, sp. n. differs mainly in the pronotal posterior corners obsolete; the clypeal emargination deeper, making teeth slightly longer; the metacoxal disc mainly smooth, scarcely bristled near the base of the femur and the postero-external corner more rounded and the parameres strongly narrowed medially. Females remain unknown.

#### Liogenys freyi

Taxon classificationAnimaliaColeopteraMelolonthidae

Cherman sp. n.

http://zoobank.org/C914E637-AD8C-4570-B145-D0936910BD6B

Figs 83 , 91

##### Type-specimen.

Holotype male, pinned, with genitalia mounted. Original labels: [white printed] “5”, [White handwritten] “Liogenys/? parvus Bl./ [printed] G. J. Arrow det.”, [green printed] “MUSEUM PARIS/CHACO DE SANTIAGO DEL ESTERO/BORDS DU RIO SALADO/ENV. D’ICAÑO/E. R. WAGNER 1910”, [red printed] “HOLOTYPE” (MNHN). Paratypes (8) bearing the label [red printed] “PARATYPE”: Female paratype labeled: [green printed] “CHACO DE SANTIAGO DEL ESTERO/LA PALISA/BORDS DU RIO SALADO/25 KIL. N. O. D’ICAÑO/E. R. WAGNER 1903”. Female paratype labeled. [green printed] “MUSEUM PARIS/CHACO DE SANTIAGO DEL ESTERO/ BORDS DU RIO SALADO/ENV. D’ICAÑO/E. R. WAGNER 1904”. Female paratype labeled. [green printed] “MUSEUM PARIS/CHACO DE SANTIAGO DEL ESTERO/LA PALISA DEL BRACHO/25 KIL. N. N. O. D’ICAÑO/LAGUNA MAMAITA/E. R. WAGNER 1904”. Female paratype labeled. [green printed] “MUSEUM PARIS/PROV. DE SANTIAGO/DEL ESTERO/BORDS DU RIO SALADO/ENV. D’ICAÑO. MISTOL PASO/E. R. WAGNER 1909” (MNHN). Two males and one female paratype labeled: [white printed] “S. del Estero/ Col. Wagner”. Males with genitalia mounted (MLPA). Male paratype labeled: [white handwritten] “Salta/ C. Olleros/ II.958. P. Dor”, “[esferic orange label]”, [pink printed] “Property/USNM”, [white printed] “Liogenys/ [handwritten] parvus Bl/[printed] det. G. Frey, 1968”, [yellow printed] “Museum Frey/Tutzing” Genitalia mounted (NHMB). Holotype and four paratypes deposited at MNHN, Muséum National d’Histoire Naturelle, Paris. Male paratype deposited at NHMB, Naturhistorisches Museum, Basel. Three paratypes deposited at MLPA, Museo de La Plata, La Plata.

##### Diagnosis.

**Figure 83.**
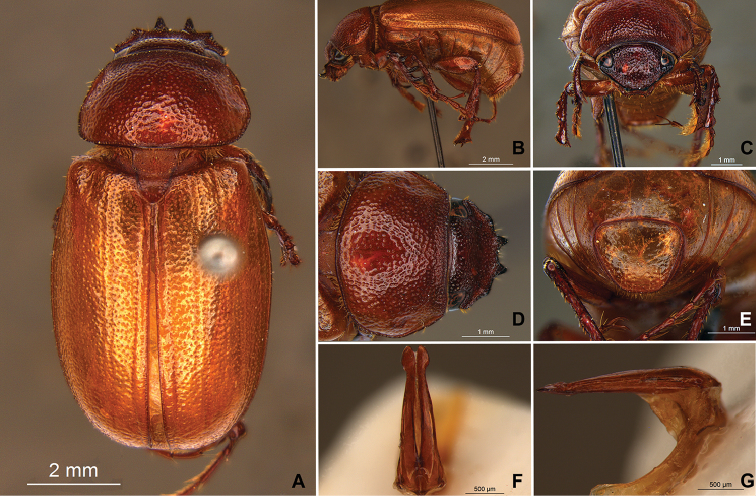
*Liogenys freyi* Cherman, sp. n. **A** Dorsal view **B** Lateral view **C** Frontal view **D** Clypeus and pronotum **E** Pygidium **F** Parameres, dorsal view **G** Parameres, lateral view.

Body light brown; elongate; elytra yellowish, pronotum reddish; clypeus quadridentate due to the tooth-like projection of the lateral margin; clypeal emargination deep, rounded and narrow; outer sides of anterior teeth parallel; clypeus bristled anteriorly; canthus exceeding the outer margin of the eye; pronotal anterior margin strongly depressed throughout; pronotal posterior corners rounded; pronotum and scutellum with inconspicuous bristles (50× magnification); mesotibia cylindrical in cross section; metacoxa scaly and smooth; male metafemur slightly produced medially on posterior margin; pygidium flat, sub-trapezoidal; pygidial disc bristled throughout, with both short and long erect bristles, punctured only on the sides; parameres, basal region very short; parameres narrowed subapically, apex spatula-like, slightly curved outwards, up to the level of the parameral basal margin (Fig. 83F).

##### Holotype.

**Male**. Length: 9.4 mm; width: 4.7 mm. Yellowish. *Head*: distance between eyes slightly more than twice the width of one eye; frons equal in length to clypeus; clypeus bristled anteriorly, emargination deep, rounded and narrow; outer sides of anterior teeth parallel; outer margin of anterior teeth as long as the eye; clypeal lateral margin convex and strongly produced forming a tooth-like projection; distance between lateral and anterior tooth longer than the basal width of anterior tooth, distance between lateral tooth and anterior margin of eye as long as one eye, right angle between anterior and lateral teeth; canthus exceeding the outer margin of the eye; distal maxillary palpomere, maximum width twice width of apex; fovea deep extending up to the transverse midline of the palpomere; labium transversely carinated, as wide as it is long; antenna 10-articulated, lamellae lighter in color and longer than the flagellum. *Thorax*: anterior margin of pronotum straight and strongly depressed throughout (Fig. 83D); maximum length of pronotum exceeding the length of tarsomeres I, II and III together; disc with inconspicuous bristles, punctures coarse and sparse, reticulated; pronotal posterior corners rounded; proepisternum bristled and scaly; mesepisternum scaly, as are the sides of metasternum, also with few long bristles on the anterior margin; distance between meso- and metacoxae up twice the length of the metacoxa; scutellum ogival, inconspicuous bristles, moderate coarsely punctured. *Elytra*: shiny, glabrous, yellowish, lighter in color than pronotum; elytra three times longer than the pronotum; elytral suture unicolored with the elytron and not elevated; the two pairs of inner ridges and the outer one more distinct than the third. *Legs*: procoxa bristled and scaly on infra-carinal and outer surface, punctures at 12× magnification; three protibial teeth, middle and apical equal in size, the three teeth equally spaced, protibial inner apical spur present; mesofemural disc glabrous, long bristles on the anterior margin; mesotibia cylindrical in cross section; disc coarsely sculptured, two mesotibial transverse carinae, the apical one complete; metacoxa smooth and scaly; basal apophysis of metacoxa produced beyond the outer margin of trochanter; male metafemur slightly produced medially on posterior margin; inner margin of metatibia carinated towards apex, apical inner surface setose; metatibial disc coarsely sculptured; two metatibial transverse carinae present posteriorly; metatibial apical spurs of different lengths, the longest one equal to the diameter of the tibial apex; protarsomere II long; pro- and mesotarsomeres I to IV enlarged, protarsomeres slightly wider than the mesotarsomeres; twice as wide as metatarsi; basal metatarsomere shorter and slightly wider than tarsomere II; claw bifid, symmetrical, superior tooth twice longer than the inferior and equally wide; distance between teeth longer than the inferior tooth. *Abdomen*: ventrites and propygidium both sparsely bristled on disc; pygidium flat, sub-trapezoidal, as wide as it is long; pygidial width not exceeding distance between spiracles of propygidium; pygidial disc bristled throughout, with both short and long erect bristles; disc smooth and punctured on sides, pygidial apex quadrate. *Parameres*: basal region very short; parameral split at 1/3; slightly wider than the parameres together at its transverse midline; inner margins straight; narrowed subapically; apex spatula-like, slightly curved outwards, up to the level of the basal outer margin (Fig. 83F). In lateral view parameres straight coplanar with basal region (Fig. 83G).

**Female** paratype (MNHN). Length: 10.4 mm; width: 5.2 mm. As the holotype except in the metafemur not medially produced on posterior margin; metacoxa longer and pygidium narrower. **Variation**. Female paratypes as the former. Male paratypes (NHMB and MLPA) length: 9.4 mm; width: 4.7 mm. As the holotype.

##### Etymology.

This species is dedicated to Georg Frey, as a reward for his enormous contribution to the knowledge of *Liogenys* in his taxonomic revision.

##### Type-locality.

ARGENTINA, Santiago del Estero: Bords du Rio Salado, Env. [environs] D’Icaño, Chaco de Santiago del Estero [Today nearby Añatuya, SE] [28°25'00.7"S, 63°00'56.7"W], E. R. Wagner, 1910.

##### Geographical distribution.

ARGENTINA (SA, SE).

##### Remarks.

*Liogenys freyi* Cherman, sp. n. resembles *L. parva* in the size and shape of the body, as well as in the quadridentate clypeus, as are *L. femella* Cherman, sp. n., *L. rotundicollis* Cherman, sp. n., and *L. piauiensis* Cherman, sp. n. *Liogenys freyi* Cherman, sp. n. differs mainly in the elytra lighter in color, clypeal emargination narrower; pronotum and scutellum with inconspicuous bristles; postero-external corner of metacoxa rounded and metacoxal disc smooth but scaly, as are the pro-, meso- and metasternum; pygidium flat, disc bristled throughout, with short and long erect bristles and the shape of parameres is also distinctive.

All these four *L. parva*-similar new species mentioned above, were erstwhile identified as *L. parva* by many taxonomists, constituting a species complex. The present work allowed their proper delimitation.

*Liogenys freyi* Cherman, sp. n. was actually first described by Frey (1969), but as a redescription of *L. parva* Blanchard (see *L. parva* remarks for more details). Unfortunately, we did not find the specimens used by Frey (1969) to redescribe the supposedly *L. parva*. Nevertheless, *L. freyi* Cherman, sp. n. paratype (NHMB) labeled from Salta, Argentina bears all the Frey's, original labels, including its identification as *L. parva*, and this specimen matches entirely with Frey's, description, so there are great chances of those type specimens mentioned by Frey (1969) also being *L. freyi* Cherman, sp. n.. In that case, when found, the locality of Formosa, Argentina will be considered a new record for this new species. Frey did not explain why he supposedly redescribed *L. parva* based on his own type series instead of Blanchard's, type series.

*Liogenys freyi* Cherman, sp. n. occurs in northern Argentina (Salta, Santiago del Estero and probably Formosa) (Fig. 91). We studied the other species from Argentina, mainly the northern ones and none of them seemed to be similar to *L. freyi* Cherman, sp. n. at all, excluding the possibility of a future case of synonymy.

#### Liogenys pseudosanctaecrucis

Taxon classificationAnimaliaColeopteraMelolonthidae

Cherman sp. n.

http://zoobank.org/A302FBC0-94C6-4281-B03F-54E88F6B81F4

Figs 84 , 93

##### Type-specimen.

Holotype male, pinned, genitalia mounted. Original labels: [white handwritten]“Me 27 (Det.)/Rio Verde, M. Grosso/10.64 [verse] Liogenys”, [white, outlined, printed] “H. and A. HOWDEN/COLLECTION/*ex.* A. Martinez coll.”, [red printed] “HOLOTYPE” (CMNC). Paratype (2) bearing the labels: [red printed] “PARATYPE”. Female paratype labeled: [white handwritten] “Me 27 (Ma)/Liogenys sp./Rio Verde, M. Grosso/10.64”, [White handwritten]“R. VERDE/MT-BRASIL/X-1964/A. MALLER”, [white printed]“DPT° ZOOL/UF-PARANÁ”, [white printed]“DZUP/401713”. Male Paratype bearing the labels: [white printed] “BRASIL: Distrito Federal/Planaltina. Embrapa Cerrados./Cerrado Nativo 15°36'16"S/ 47°44'16"W. 03-XI-2006. light./C. Oliveira”, [white printed] “CZMT-CEMT/0000018056”(CEMT). Holotype deposited at CMNC, Canadian Museum of Nature, Ottawa. Female paratype deposited in DZUP, Coleção Entomologica Pe. Jesus Santiago Moure, Universidade Federal do Paraná, Curitiba. Male paratype deposited in CEMT, Setor de Entomologia da Coleção Zoológica, Universidade Federal de Mato Grosso, Cuiabá.

##### Diagnosis.

**Figure 84.**
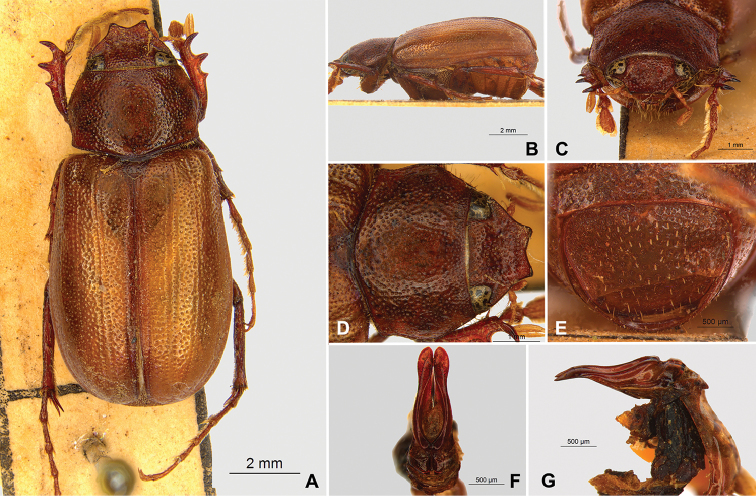
*Liogenys pseudosanctaecrucis* Cherman, sp. n. **A** Dorsal view **B** Lateral view **C** Frontal view **D** Clypeus and pronotum **E** Pygidium **F** Parameres, dorsal view **G** Parameres, lateral view.

Body brownish, elytra testaceous, pronotum darker; sides parallel; clypeal emargination sub-angled, deep and wide; outer sides of anterior teeth parallel; meso- and metatibia quadrate in cross section. In males, pygidial disc scaly throughout, erect-scaled; pygidial apex rounded; parameres deeply grooved across the basal region, inner margins forming an elevated flange, apex widened and rounded (Fig. 84F).

##### Holotype.

**Male**. Length: 10.7 mm; width 5.1 mm. *Head*: distance between eyes twice the width of one eye; frons shorter than clypeus; clypeal emargination deep, sub-angled and wide, outer sides of anterior teeth parallel; outer margin of anterior teeth equal to the eye; lateral margin slightly convex; canthus not exceeding the outer margin of the eye; distal maxillary palpomere, maximum width equal to width of apex; fovea deep, extending past the transverse midline of the palpomere; labium transversely carinated, as wide as it is long; antenna 10-articulated, lamellae longer than flagellum. *Thorax*: maximum length of pronotum exceeding the length of tarsomeres I, II and III together; disc glabrous, punctures coarse and sparse; pronotal posterior corners sharp, right-angled; proepisternum with long bristles, pro- and mesepisternum scaly, as are the sides of metasternum, also with few long bristles on the anterior margin; distance between meso- and metacoxae up to twice the length of the metacoxa; scutellum ogival, coarsely punctured except on the center. *Elytra*: shiny, glabrous, uniformly testaceous, pruinose on the posterior margin, lighter in color than the pronotum; elytra more than three times longer than the pronotum; elytral suture slightly darker than elytron and weakly elevated; three pairs of elytral ridges barely noticeable, except of the outer one, distinctly elevated. *Legs*: procoxa bristled on infra-carinal and outer surface; three protibial teeth, middle and apical equal in size, the three teeth equally spaced; protibial inner apical spur present; mesofemural disc glabrous, with a row of long bristles on the anterior and posterior margins; mesotibia quadrate in cross section; disc finely sculptured; basal apophysis of metacoxa produced beyond the outer margin of trochanter; inner margin of metatibia carinated towards apex, apical inner surface setose; metatibial disc finely sculptured; metatibial short transverse carina present posteriorly; metatibial apical spurs equal in length, slightly shorter than the diameter of the tibial apex; protarsomere II long; pro- and mesotarsomeres I to IV enlarged, protarsomeres slightly wider than the mesotarsomeres and more than twice wider than metatarsi; basal metatarsomere slightly wider and shorter than tarsomere II; claws bifid, symmetrical; superior tooth longer and narrower than inferior tooth, distance between teeth shorter than the inferior tooth. *Abdomen*: band of scales visible at the lowest magnification beneath the outer margin of elytra; ventrites bristled abundantly on disc and sides; propygidium bristled; pygidium flat, wide; pygidial width exceeding distance between spiracles of propygidium, pygidial disc with yellow erect scales throughout and deep umbilical coarse punctures; pygidial apex rounded. *Parameres*: basal region broadly grooved across the midline; basal region wider than parameres together at its maximum width, parameral split at 2/3; apex widened, edges rounded, inner margins convergent with a flange elevated, mainly in the basal region (Fig. 84F); in lateral view parameres concave; apex curved downwards apically (Fig. 84G).

**Female** paratype. Length: 10.7 mm; width 5.7 mm. As the holotype except in the body wider and shiny; clypeal emargination narrower; pronotal and elytral punctures slightly coarser, scutellum wider and triangular, pygidium sub-trapezoidal; pygidial apex with sharper edges and bristled instead of scaly, bristles more abundant and longer on apex.

##### Variation.

Male paratype. Length: 10.7 mm; width 5.2 mm. The paratype does not differ significantly from the holotype, except in the elytral outer ridge less conspicuous and the elytra shiny, not pruinose, on posterior margins.

##### Etymology.

Noun in the genitive case. Prefix from Ancient Greek ψευδής (pseudḗs, “false, lying”). The species name is due to the morphological similarity with *L. santaecrucis* and also their geographical distribution is nearby.

##### Type-locality.

BRAZIL, Mato Grosso do Sul: Rio Verde de Mato Grosso [18°55'33.2"S, 54°50'43.6"W], Oct 1964.

##### Geographical distribution.

BRAZIL (MS, DF).

##### Remarks.

*Liogenys pseudosanctaecrucis* Cherman, sp. n. shares with *L. santaecrucis* almost all of the external features, excepting the male pygidial disc scaly instead of bristled. The parameres differ in the more pronounced and broader groove across the basal region.

#### Liogenys grossii

Taxon classificationAnimaliaColeopteraMelolonthidae

Cherman sp. n.

http://zoobank.org/EC683038-11B0-4B38-B6BE-E06D470E6CF9

Figs 85 , 90

##### Type-specimen.

Holotype male, pinned, with genitalia mounted. Original labels: [white printed] “BRASIL. BA/Encruzilhada. Estr. SE/16. XII. 2012. Luz/ P. C. Grossi Coll”, [red printed] “HOLOTYPE” (CERPE). Paratypes (5) bearing the label [red printed] “PARATYPE”: Female paratype labeled: [white printed] “BRASIL, BA, Encruzilhada/15°34'35"S 40° 56'51"W/ 15.xii.2012, J.A.Rafael and/ E.J.Grossi, Arm. Luz, 850m” (INPA). Female paratype labeled. [White printed] “BRASIL. MG. Aguas/ Vermelhas. Faz. Faccino/ 12.XII. 2012. Luz/ P. C. Grossi Coll” (CERPE). Three female paratypes labeled: [white printed]“BRASIL: Bahia, Encruzilhada,/ Divisa, [long space] XI.1975”. (MNRJ). Holotype and one paratype deposited at CERPE, Coleção Entomologica da Universidade Federal Rural de Pernambuco, Recife. Female paratype deposited at INPA, Instituto Nacional de Pesquisa Amazônica, Manaus; and three female paratypes at MNRJ, Museu Nacional de Rio de Janeiro, Rio de Janeiro.

##### Diagnosis.

**Figure 85.**
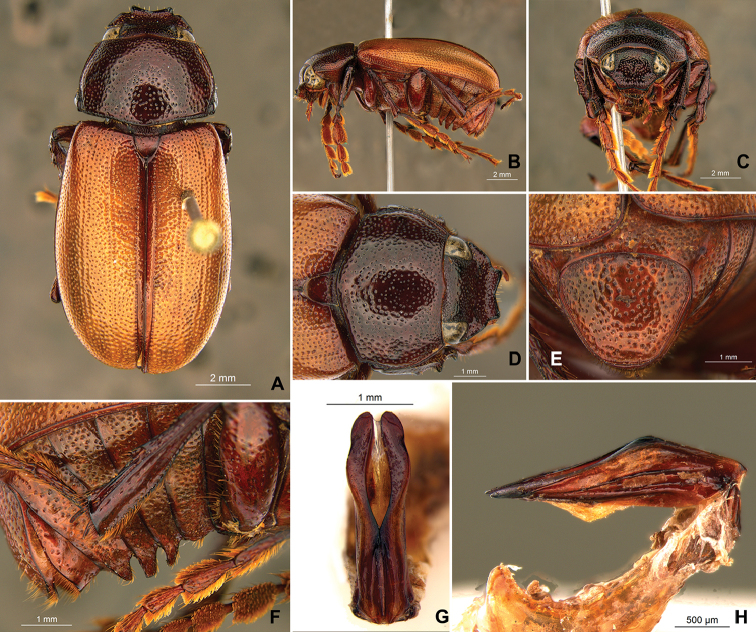
*Liogenys grossii* Cherman, sp. n. **A** Dorsal view **B** Lateral view **C** Frontal view **D** Clypeus and pronotum **E** Pygidium **F** Abdomen, lateral view **G** Parameres, dorsal view **H** Parameres, lateral view.

Body brownish; elongate; elytra testaceous, pronotum purplish red, sparsely punctured; clypeal emargination sharp and wide; outer sides of anterior teeth parallel; clypeal lateral margin convex, with a sub-angled tooth-like projection, right angle between outer side of anterior teeth and clypeal lateral projection; mesotibia quadrate in cross section; inner margin of male metatibia medially produced; ventrites III to V strongly produced medially (Fig. 85F); pygidium flat, as wide as it is long; pygidial disc bristled only on apex, coarsely punctured; male genitalia, basal region narrower than the width of the parameres together at its maximum width, parameres narrowed subapically, apical edges rounded (Fig. 85G).

##### Holotype.

**Male**. Length: 13.3 mm; width: 6.4 mm. Elytra testaceous, pronotum purplish red. *Head*: distance between eyes nearly twice the width of one eye; frons swollen, equal in length to clypeus; clypeal emargination sharp, shallow and wide; outer sides of anterior teeth parallel; outer margin of anterior teeth three times shorter than the eye; clypeal lateral margin convex, with a sub-angled tooth-like projection; distance between clypeal lateral projection and anterior margin of eye slightly shorter than one eye; distance between clypeal lateral projection and anterior tooth shorter than basal width of anterior tooth; right angle between outer side of anterior teeth and clypeal lateral projection; canthus not exceeding the outer margin of the eye; distal maxillary palpomere, maximum width as wide as the apex; fovea shallow, elongate, extending past the transverse midline of the palpomere; labium transversely carinated, as wide as it is long; antenna 10-articulated, lamellae slightly lighter in color and longer than flagellum. *Thorax*: anterior margin of pronotum slightly produced medially; maximum length of pronotum exceeding the length of tarsomeres I, II and III together; disc glabrous, punctures fine and very sparse; pronotal posterior corners sharp, almost right-angled; proepisternum with short bristles; mesepisternum scaly; sides of metasternum bristled; distance between meso- and metacoxae up to twice longer than the metacoxa; scutellum ogival, finely punctured at the base. *Elytra*: shiny, glabrous, uniformly testaceous, distinctly lighter in color than pronotum; elytra more than three times longer than the pronotum; elytral suture darker than elytron and distinctly elevated; all four elytral ridges barely noticeable. *Legs*: procoxa bristled on infra-carinal and outer surface; punctures visible at 12× magnification; three protibial teeth, middle and apical equal in size, the three teeth equally spaced; protibial inner apical spur present; mesofemural disc setose, scarcely bristled; mesotibia quadrate in cross section, disc coarsely sculptured; two mesotibial transverse carinae, the apical one incomplete; basal apophysis of metacoxa produced beyond the outer margin of trochanter; inner margin of metatibia carinated and abruptly medially produced towards apex; apical inner surface setose; metatibial disc finely sculptured; metatibial transverse carina present posteriorly; metatibia with posterior discontinuous longitudinal carina; metatibial spurs equal in length, length equal to the diameter of the tibial apex; protarsomere II long; pro- and mesotarsomeres I to IV enlarged, protarsomeres two-fold wider than the mesotarsomeres and more than three times wider than the metatarsi; basal metatarsomere longer than tarsomere II and as wide as; claw bifid, symmetrical, superior tooth longer and narrower than the inferior; distance between teeth shorter than the inferior tooth. *Abdomen*: ventrites bristled on disc and sides; ventrites III, IV and V strongly produced medially; propygidium visible, glabrous; pygidium flat; sub-quadrate, as wide as it is long; pygidial width not exceeding distance between spiracles of propygidium; pygidial disc bristled only on apex, coarsely punctured; pygidial apex quadrate. *Parameres*: basal region narrower than the parameres together at its maximum width, parameral split at the third portion; inner margins convergent; narrowed subapically; apical edges rounded (Fig. 85G). In lateral view parameres slightly concave (Fig. 85H).

**Female** paratype. Length: 13.9 mm; width: 7.2 mm. As the holotype except in the size bigger, body wider and darker, clypeal lateral margin weakly produced; ventrites not produced medially and pygidium convex.

##### Etymology.

Noun in the genitive case. The species is dedicated to its collector, Dr Paschoal Coelho Grossi, great Scarabaeoidea researcher and author of numerous species within the superfamily.

##### Type-locality.

BRAZIL, Bahia: Encruzilhada [40°56'51"W, 15°34'35"S], from light, 16 Dec 2012, P. C. Grossi Coll.

##### Geographical distribution.

BRAZIL (BA, MG).

##### Remarks.

*Liogenys grossii* Cherman, sp. n. resembles *L. spiniventris* (Fig. 73) in the size, shape and color of the body and elytra, shape of clypeus and mainly in the presence of medial projections on ventrites. The new species differs in the additional medial projection on ventrite and in the shape of the parameres (Fig. 85F), narrowed subapically.

#### Liogenys pseudospiniventris

Taxon classificationAnimaliaColeopteraMelolonthidae

Cherman sp. n.

http://zoobank.org/5A05EFDD-8D9B-4ED0-9E72-9A925B8581D1

Figs 86 , 90

##### Type-specimen.

Holotype male, pinned, with genitalia mounted. Original labels: [white, outlined, printed] “Morro da Garça/MG-Brasil/ 18- 20.X.1964/ Exp. Dep. Zool.”, [white, handwritten] “Liogenys/spiniventris/Mos/ [printed] det. G. Frey, 1970”, [red printed] “HOLOTYPE” (NHMB). Female Paratype (1) bearing the labels: [red printed] “PARATYPE”, [green printed] “Brasilien/Prov. S. Paulo/ Campinas/ Alwine Braatz V.” (ZMHB). Holotype deposited at NHMB, Naturhistorisches Museum, Basel. Female paratype deposited at ZMHB, Museum für Naturkunde der Leibniz Gemeinschaft Institut, Berlin.

##### Diagnosis.

**Figure 86.**
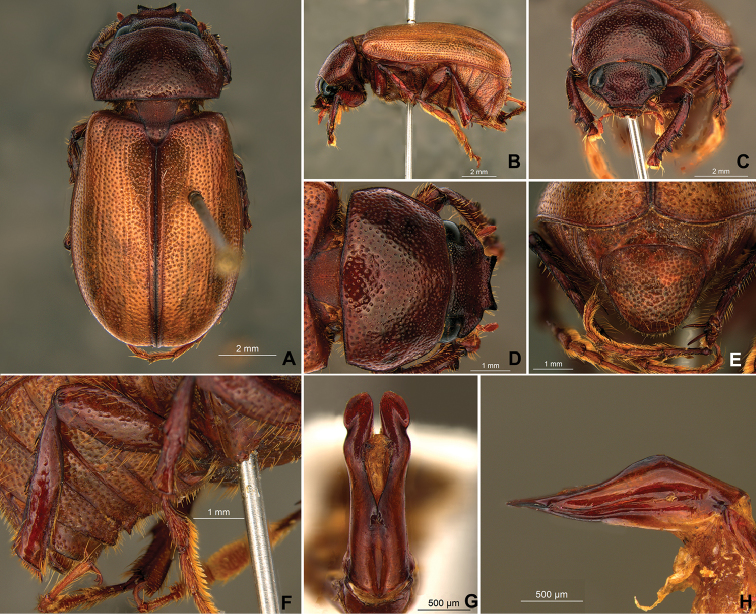
*Liogenys pseudospiniventris* Cherman, sp. n. **A** Dorsal view **B** Lateral view **C** Frontal view **D** Clypeus and pronotum **E** Pygidium **F** Abdomen, lateral view **G** Parameres, dorsal view **H** Parameres, lateral view.

Body brownish; elongate; elytra testaceous, pronotum purplish red, sparsely punctured; clypeal emargination sharp and wide; outer sides of anterior teeth sub-parallel, slightly divergent; clypeal lateral margin with a rounded tooth-like projection, acute angle between outer side of anterior teeth and clypeal lateral projection; canthus exceeding the outer margin of the eye; mesotibia quadrate in cross section; male metafemur medially produced on posterior margin; male metatibia slightly bent outwards, with inner margin medially produced towards apex; male ventrites IV and V strongly produced medially, the former is spine-like apically; pygidium flat, as wide as it is long, bristled throughout; male genitalia with basal region narrower than the parameres together at its maximum width, parameres strongly narrowed subapically (Fig. 86G).

##### Holotype.

**Male**. Length: 11.9 mm; width: 6.0 mm. Testaceous, pronotum purplish red. *Head*: distance between eyes nearly twice the width of one eye; frons equal in length to clypeus; clypeal emargination sharp, shallow and wide; outer sides of anterior teeth sub-parallel, slightly divergent; outer margin of anterior teeth more than twice shorter than the eye; clypeal lateral margin convex, with a rounded tooth-like projection; distance between clypeal lateral projection and anterior margin of eye slightly shorter than one eye; distance between clypeal lateral projection and anterior tooth shorter than basal width of anterior tooth; angle between outer side of anterior teeth and clypeal lateral projection slightly acute; canthus exceeding the outer margin of the eye; distal maxillary palpomere, maximum width slightly wider than the apex; fovea shallow, elongate, extending past the transverse midline of the palpomere; labium transversely carinated, as wide as it is long; antenna 10-articulated, lamellae lighter in color and longer than flagellum. *Thorax*: anterior margin of pronotum slightly produced medially; maximum length of pronotum exceeding the length of tarsomeres I, II and III together; disc weakly sulcated posteriorly, glabrous, punctures moderately coarse and sparse; pronotal posterior corners sharp, obtuse-angled; proepisternum with short and a few long bristles; mesepisternum scaly; sides of metasternum bristled; distance between meso- and metacoxae up to twice longer than the metacoxa; scutellum ogival, finely punctured at the sides. *Elytra*: shiny, glabrous, uniformly testaceous, distinctly lighter in color than pronotum; elytra more than three times longer than the pronotum; elytral suture darker than elytron and distinctly elevated; all four elytral ridges barely noticeable. *Legs*: procoxa bristled on infra-carinal and outer surface; smooth at 12× magnification; three protibial teeth, the middle the longest, the three teeth equally spaced; protibial inner apical spur present; mesofemural disc setose, scarcely bristled; mesotibia quadrate in cross section, disc finely sculptured; two mesotibial transverse carinae, the apical complete; metacoxal disc bristled; basal apophysis of metacoxa produced beyond the outer margin of trochanter; metafemur medially produced on posterior margin; metatibia slightly bent outwards, inner margin of metatibia carinated and medially produced towards apex, apical inner surface setose; metatibial disc finely sculptured; two metatibial transverse carinae present posteriorly and posterior discontinuous longitudinal carina; metatibial spurs equal in length, shorter than the diameter of the tibial apex; pro- and mesotarsomeres I to IV enlarged, mesotarsomeres less than three times wider than the metatarsi; basal metatarsomere longer than tarsomere II and as wide as; claw bifid, symmetrical, superior tooth longer and narrower than the inferior; distance between teeth shorter than the inferior tooth. *Abdomen*: ventrites bristled abundantly on disc and sides; ventrites IV and V strongly produced medially, the former is spine-like apically; propygidium visible and bristled; pygidium flat; sub-quadrate, as wide as it is long; pygidial width not exceeding distance between spiracles of propygidium; pygidial disc with erect bristles throughout; pygidial apex quadrate. *Parameres*: basal region narrower than the width of the parameres together at its maximum width, parameral split at 2/3; inner margins of parameres convergent; strongly narrowed subapically, apical edges rounded (Fig. 86G). In lateral view parameres concave (Fig. 86H).

**Female paratype**. Length: 13.9 mm; width: 7.2 mm. As the holotype except in the size bigger, frons more swollen, clypeus lateral margin weakly produced, metafemur and metatibia not produced on inner margin and ventrites not produced medially.

##### Etymology.

Adjective in the nominative singular. Prefix from Ancient Greek ψευδής (pseudḗs, “false, lying”); compound noun from Latin *spīna* (“thorn”) + *ventris* genitive from *venter* (abdomen). Species named in reference to the ventral feature alike with *L. spiniventris*. These species share the ventrites IV and V medially furnished with projections.

##### Type-locality.

BRAZIL, Minas Gerais: Morro da Garça [44°36'09"W, 18°32'49"S], 18–20 Oct 1964.

##### Geographical distribution.

BRAZIL (MG, SP).

##### Remarks.

*Liogenys pseudospiniventris* Cherman, sp. n. resembles *L. spiniventris* (Fig. 73) in the size, shape and color of the body and elytra, shape of clypeus and mainly in the presence of medial projections of ventrites. The new species differs from *L. spiniventris* mainly in the pygidial disc bristled throughout; male ventrite IV projection spine-like apically and in the shape of the parameres, strongly narrowed subapically. *Liogenys pseudospiniventris* Cherman, sp. n. female paratype is not from the same collection as the holotype and also differs in the type-locality. However, many features common to male and female were observed and allowed the matching, as the color of elytra and pronotum; frons swollen, shape of the clypeus, pronotal disc weakly sulcated posteriorly, density and distribution of punctures; pronotal posterior corners sharp; mesotibia quadrate in cross section and pygidial disc bristled throughout.

#### Liogenys sulcoventris

Taxon classificationAnimaliaColeopteraMelolonthidae

Cherman sp. n.

http://zoobank.org/57162FCF-26E6-4F37-BB30-1602B0BED159

Figs 87 , 90

##### Type-specimen.

Holotype male, pinned, wih genitalia mounted. Original labels: [white printed]“Coleção/A.M.BELLO”, [white printed] “Nova Friburgo/RJ - BRASIL/XII - 2002/ Col: E. Grossi”, [white printed] “CZMT-CEMT/0000018010”, [red printed] “HOLOTYPE” (CEMT). Paratypes (6) bearing the label [red printed] “PARATYPE”: Female paratype labeled: [white printed] “Macaé de Cima/Nova Friburgo/ RJ - BRASIL/ I-2006/ Leg. B. Miller”, [white printed] “CZMT-CEMT/0000018011”. Female paratype labeled: [white printed] “Macaé de Cima/Nova Friburgo/ RJ - BRASIL/ I-2006/ Leg. B. Miller”, [white printed] “CZMT-CEMT/0000018013” (CEMT). Two male and two female paratypes labeled: [white printed]“BRASIL. RJ. N. Friburgo/Macaé de Cima/XI. 2005” “1400m./-22.3752S; -42.4957W/P. C. Grossi Coll.” (CERPE). Holotype and two paratypes deposited at CEMT, Setor de Entomologia da Coleção Zoológica, Universidade Federal de Mato Grosso, Cuiabá. Four paratypes deposited at CERPE, Coleção Entomologica da Universidade Federal Rural de Pernambuco, Recife.

##### Non-type material.

BRAZIL. MG: Mar de Espanha, 6/II/1910, J. F. Zikan col., 1 ex. (CEIOC); Virgínia, Faz. Dos Campos, 1500m, without date, J. F. Zikan col., 1 ex. (MNRJ); Passa Quatro: Faz dos Campos, 12/XI/1917, J. F. Zikan col., 1 ex. (MNRJ); without date, J. F. Zikan col., 1 ex. (CEIOC); ES: Itaguaçú, 14/V/1964, C. Elias col., 1 ex. (DZUP); Santa Teresa, 6/XI/1966, C.T. and C. Elias col., 1 ex. (DZUP); Alegre. Faz. Jerusalém, 24/IX/1914, J. F. Zikan col., 1 ex. (CEIOC); RJ: Rio de Janeiro, 1844, M de Castelnau col., 1 ex. (MNHN); Nova Friburgo, without date and collector, Hüsing Halle leg., PARATYPE Liogenys palmata Burm, 1855 G.Frey 1967/68 det., 1 ex. (MLUH); Copacabana: XI/1996, A. Bello col., 1 ex.; XI/2009, A. Bello col., 1 ex.; XI/2009, A. Bello col., 1 ex. (CEMT); Visconde de Mauá, XII/1991, A. Bello col., 2 ex. (CEMT); SP: Pindamonhangaba, Eugênio Lefevre, [22°49'32"S, 45°37'43"W], 29/IX/1963, alt. 1161.7 m, Exp.Dep. Zool. col., Liogenys palmata Burm, 1855 G. Frey 1967/69 det., 2 ex. (MZSP); Eugênio Lefevre trav., alt. 1161.7 m, without date, Lopes et Oiticica col., 1 ex. (MNRJ); São Miguel Arcanjo. Parque Estadual Carlos Botelho, 24°04'01"S, 47°59'40"W, 25/XI/2011, 762 m, E. Bovy col., tapir faeces, 1 ex. (CEMT); PR: Castro, 21/XII/1955, Laroca col., 1 ex. (DZUP); Arapoti, X/1937, A. Maller col. 2 ex. (MNRJ); Guarapuava, I/1960 Schneider col., 1 ex. (MNRJ); without locality, without date, Metzler leg., 1 ex.; Hilarianus anguliceps Moser det., Metzler leg., 1 ex. (SDEI); without date, Exp.Dep. Zool. col., Hüsing Halle leg., PARATYPE Liogenys palmata Burm, 1855 G.Frey 1967/68 det., 1 ex. (MHLU); without date and collector, Liogenys testaceipennis Moser Frey 1968 det., 1 ex. (NMHB); 5/XI/1920, J. F. Zikan col., 1 ex.; 14/XI/1919, J. F. Zikan col., 1 ex. (CEIOC).

##### Diagnosis.

**Figure 87.**
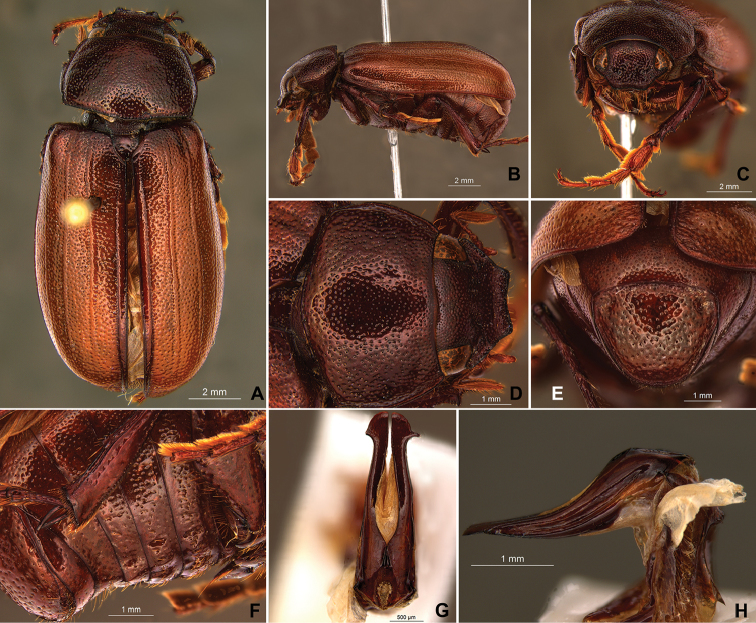
*Liogenys sulcoventris* Cherman, sp. n. **A** Dorsal view **B** Lateral view **C** Frontal view **D** Clypeus and pronotum **E** Pygidium **F** Abdomen, lateral view **G** Parameres, dorsal view **H** Parameres, lateral view.

Body brownish; elongate; elytra testaceous to brownish, pronotum reddish or dark brown; clypeal emargination rounded, shallow and wide; outer sides of anterior teeth sub-parallel; canthus exceeding the outer margin of the eye; male mesotibia quadrate in cross section; male metafemur strongly produced medially on posterior margin; metatibia slightly bent outwards; inner margin of male metatibia medially produced towards apex; claw bifid, symmetrical, superior tooth as long as and narrower than the inferior; male ventrites I, II and III sulcated medially, IV slightly elevated; pygidium convex or flat, as wide as it is long, pygidial disc bristled only on apex; male genitalia, width of basal region equal to the parameres together at its transverse midline; total length of parameres more than five times the length of their apex; inner margins convergent; apex harpoon-like with lateral angle curved projecting perpendicularly to parameres (Fig. 87G).

##### Holotype.

**Male**. Length: 14.7 mm; width: 7.2 mm. Brownish. *Head*: distance between eyes nearly twice the width of one eye; frons equal in length to clypeus, finely and densely punctured; clypeal emargination rounded, shallow and wide; outer sides of anterior teeth sub-parallel, slightly convergent; outer margin of anterior tooth more than three times shorter than the eye; clypeal lateral margin slightly convex; canthus exceeding the outer margin of the eye; distal maxillary palpomere, maximum width nearly the width of apex; fovea shallow, extending up to the transverse midline of the palpomere; labium transversely carinated, as wide as it is long; antenna 10-articulated, lamellae lighter in color and longer than flagellum. *Thorax*: anterior margin of pronotum slightly produced medially; maximum length of pronotum exceeding the length of tarsomeres I, II and III together; disc glabrous, punctures fine and sparse; pronotal posterior corners sharp, almost right-angled; proepisternum with short bristles; mesepisternum scaly; sides of metasternum bristled, a few long ones on the anterior margin; distance between meso- and metacoxae up to twice longer than the metacoxa; scutellum ogival, finely punctured along the margins. *Elytra*: shiny, glabrous, uniform light brown to testaceous, pronotum dark brown; elytra more than three times longer than the pronotum; elytral suture slightly darker than elytron and distinctly elevated; two pairs of inner ridges more noticeable than the two outer pairs. *Legs*: procoxa scaly on infra-carinal and outer surface; punctures visible at 12× magnification; three protibial teeth, middle and apical equal in size, the three teeth equally spaced; protibial inner apical spur present; mesofemural disc setose, scarcely bristled; mesotibia quadrate in cross section; disc coarsely sculptured, mesotibial apical carina incomplete; basal apophysis of metacoxa produced beyond the outer margin of trochanter; metafemur strongly produced medially on posterior margin; inner margin of metatibia carinated and medially produced towards apex, apical inner surface setose; metatibial disc finely sculptured; two metatibial transverse carina present posteriorly and posterior discontinuous longitudinal carina; metatibial apical spurs of different lengths, the longest shorter than the diameter of the tibial apex; protarsomere II long; pro- and mesotarsomeres I to IV enlarged, protarsomeres two-fold wider than the mesotarsomeres and more than twice than the metatarsi; basal metatarsomere and tarsomere II equal in size; claw bifid, symmetrical, superior tooth as long as and narrower than the inferior; distance between teeth shorter than the inferior tooth. *Abdomen*: ventrites bristled on disc; ventrites I, II and III sulcated medially, IV slightly elevated; propygidium visible, bearing few short bristles; pygidium convex, sub-quadrate, as wide as it is long; pygidial width not exceeding distance between spiracles of propygidium, pygidial disc bristled only on apex; pygidial apex quadrate. *Parameres*: width of basal region equal to the parameres together at its midline, parameral split at 2/3; total length of parameres more than five times the length of their apex; inner margins convergent; apex harpoon-like with lateral angle curved projecting perpendicularly to parameres (Fig. 87G). In lateral view parameres strongly concave (Fig. 87H).

**Female** paratype. Length: 16.3 mm; width 8.3 mm. As the holotype except in the size bigger, body darker; frons more swollen, clypeal lateral margin straight; elytra pruinose; head punctures coarser; scutellum barely punctured at the base; ventrites smooth.

##### Variation.

Male paratypes 14.6–14.8 mm; width: 7.2 mm. As the holotype except in the pygidium, convex or flat; female paratypes as the former.

##### Etymology.

Adjective in the nominative singular. From Latin *sulcus* (“furrow, ditch, track”) + *ventris* genitive from *venter* (“abdomen”). The species is named in reference to the shape of the male ventrites sulcate medially

##### Type-locality.

BRAZIL, Rio de Janeiro: Nova Friburgo [42°29'45"W; 22°22'31"S], Dec 2002, E. Grossi Coll..

##### Geographical distribution.

BRAZIL (MG, ES, RJ, SP, PR).

##### Remarks.

*Liogenys sulcoventris* Cherman, sp. n. resembles *L. punctaticollis* (Fig. 69) and *L. tibialis* (Fig. 77) in the size, shape and color of the body and elytra and in the shape of clypeus. The new species differs mainly in the head punctures which are fine and dense; pronotal punctures denser and clypeal emargination rounder. In males the ventrites I, II and III are sulcated medially and the ventrite IV is slightly elevated (Fig. 87F) (in *L. tibialis* ventrites I to IV are elevated and in *L. puncaticollis* they are smooth); the pygidium is wider apically and the shape of parameres is distinct, more than five times the length of their apex, which is harpoon-like with lateral angle curved projecting perpendicularly to parameres. Within the type series (MLUH) of *L. palmata* (junior synonym of *L. punctaticollis*), there are two paratypes that match with *L. sulcoventris* Cherman, sp. n. and they are also from Nova Friburgo. However, the type-locality is not a conclusive data, as *L. punctaticollis* also occurs at the same locality.

**Figure 88.**
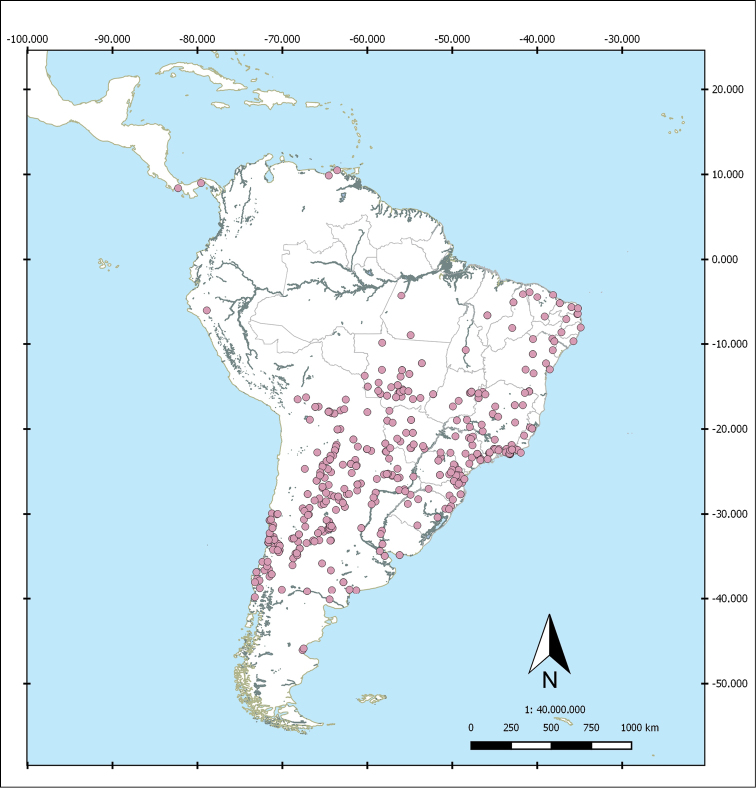
Geographical distribution of *Liogenys* Guérin-Méneville.

**Figure 89.**
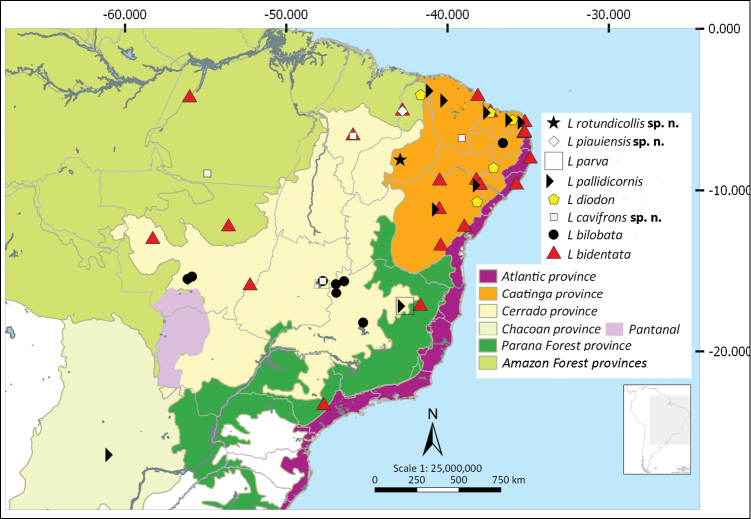
Distribution records of eight species of *Liogenys*. Biomes based on Morrone (2014).

**Figure 90.**
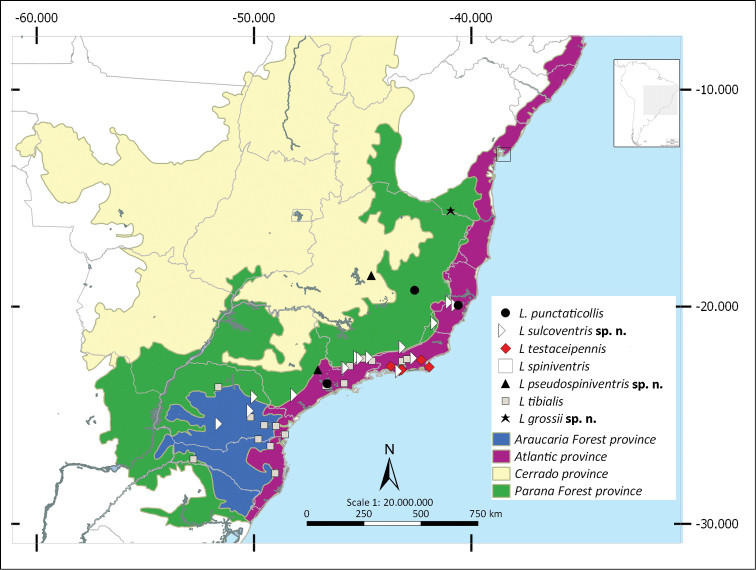
Distribution records of seven species of *Liogenys*. Biomes based on Morrone (2014).

**Figure 91.**
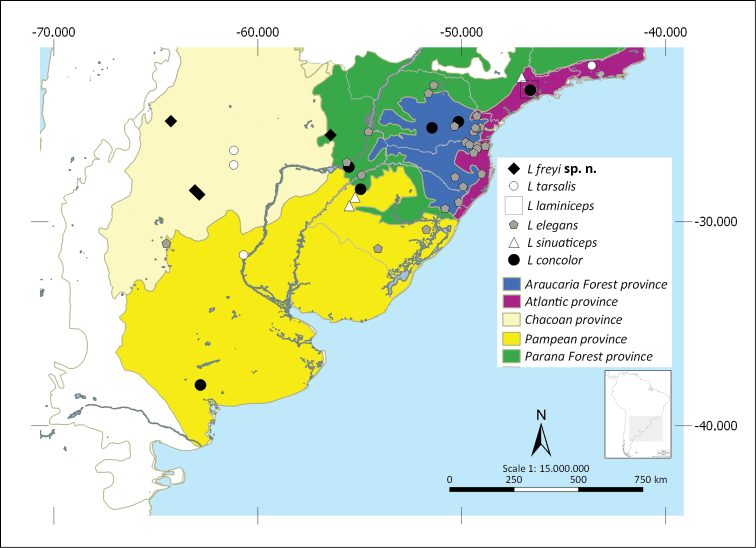
Distribution records of six species of *Liogenys*. Biomes based on Morrone (2014).

**Figure 92.**
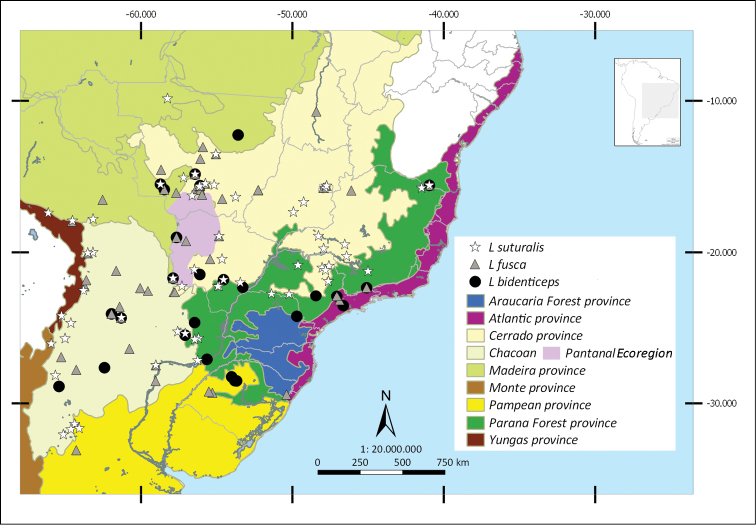
Distribution records of three species of *Liogenys*. Biomes based on Morrone (2014).

**Figure 93.**
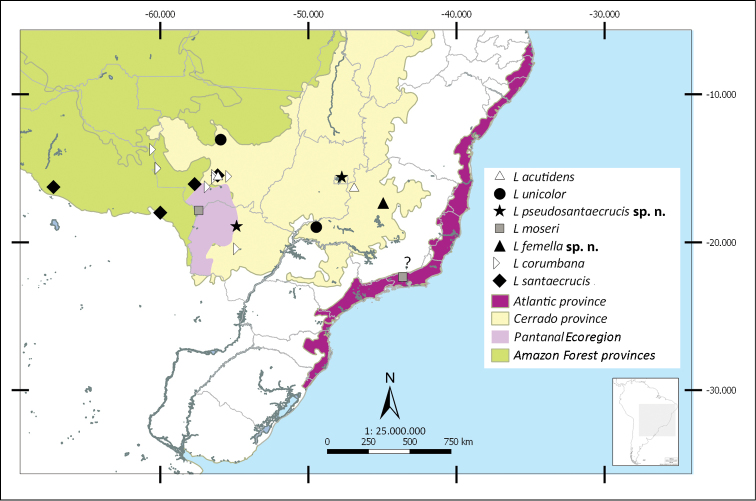
Distribution records of seven species of *Liogenys*. Biomes based on Morrone (2014).

## Conclusions

*Liogenys* Guérin-Méneville, 1831 is recognized by the following features: frons plus clypeus forming a concavity; clypeus emarginate and toothed anteriorly, with frontoclipeal impressions, clypeal lateral margin sinuous and sometimes toothed; protarsomere I shorter than protarsomere II and pygidial disc with umbilicate punctures. Twenty-three Brazilian *Liogenys* are redescribed: *L. acutidens* Moser, *L. bidentata* Burmeister, *L. bidenticeps* Moser, *L. bilobata* Frey, *L. concolor* Blanchard, *L. corumbana* Moser, *L. diodon* Burmeister, *L. elegans* Nonfried, *L. fusca* Blanchard, *L. laminiceps* Moser; *L. moseri* Frey, *L. pallidicornis* Blanchard, *L. parva* Blanchard, *L. punctaticollis* (Blanchard), *L. rufocastanea* Moser, *L. santaecrucis* Blanchard, *L. sinuaticeps* Moser, *L. spiniventris* Moser, *L. suturalis* (Blanchard), *L. tarsalis* Moser; *L. testaceipennis* Moser, *L. tibialis* Moser and *L. unicolor* Evans. Nine new species are described: *L. cavifrons* Cherman, sp. n., *L. femella* Cherman, sp. n., *L. piauiensis* Cherman, sp. n., *L. rotundicollis* Cherman, sp. n., *L. pseudosanctaecrucis* Cherman, sp. n., *L. freyi* Cherman, sp. n., *L. grossii* Cherman, sp. n., *L. pseudospiniventris* Cherman, sp. n. and *L. sulcoventris*, Cherman sp. n. Five junior subjective synonyms are proposed: *L. bicuspis* Moser of *L. bidenticeps*; *L. forsteri* Frey of *L. elegans*; *L. obesa* Burmeister of *L. concolor*; *L. peritryssoidea* Keith of *L. santaecrucis*; and *L. seabrai* Martínez of *L. testaceipennis* Moser. *Liogenys tibialis* Moser is Stat. Rest. Nineteen lectotypes are designated: *Liogenys argentina* Moser, *L. bicuspis* Moser, *L. bidentata* Burmeister, *L. bidenticeps* Moser, *L. brasiliensis* Moser, *Liogenys concolor* Blanchard, *L. corumbana* Moser, *L. cuyabana* Moser, *L. elegans* Nonfried, *L. fusca* Blanchard, *L. moseri* Frey, *L. obesa* Burmeister, *L. pallidicornis* Blanchard, *L. parva* Blanchard, *L. rufocastanea* Moser, *L. santaecrucis* Blanchard, *Hilarianus suturalis* Blanchard, *L. tarsalis* Moser and *Hilarianus concolor* Blanchard [for *Liogenys unicolor* Evans]. New geographical records are registered for the following nineteen species: *L. acutidens* (Brazil: MG); *L. bidentata* (Brazil: PA, MA, CE, PI, RN, PE, AL, SE, BA, MG, GO, MT); *L. bidenticeps* (Brazil: BA, PR; Paraguay, Argentina); *L. bilobata* (Brazil: PB, MT, DF, SP); *L. concolor* (Argentina); *L. corumbana* (Brazil: MT); *L. diodon* (Brazil: PI, CE, PE, SE, GO); *L. elegans* (Brazil: PR, SC, Paraguay, Argentina), *L. fusca* (Brazil: TO, BA, MG, SP; Bolivia; Argentina: SA, TU, FO, CH, CR, CO); *L. moseri* (Brazil: MT); *L. pallidicornis* (Brazil: CE, RN, SE, BA; Argentina); *L. parva* (Brazil: MG); *L. punctaticollis* (Brazil: ES, MG, SP); *L. sinuaticeps* (Brazil: SP); *L. santaecrucis* (Bolivia: LP); *L. suturalis* (Brazil: BA, SP, PR; Paraguay, Bolivia, Argentina); *L. tarsalis* (Argentina); *L. tibialis* (Brazil: MG, SP, PR, SC); *L. unicolor* (Brazil: MT).

## Supplementary Material

XML Treatment for Liogenys

XML Treatment for Liogenys acutidens

XML Treatment for Liogenys bidentata

XML Treatment for Liogenys bidenticeps

XML Treatment for Liogenys bilobata

XML Treatment for Liogenys concolor

XML Treatment for Liogenys corumbana

XML Treatment for Liogenys diodon

XML Treatment for Liogenys elegans

XML Treatment for Liogenys fusca

XML Treatment for Liogenys laminiceps

XML Treatment for Liogenys moseri

XML Treatment for Liogenys pallidicornis

XML Treatment for Liogenys parva

XML Treatment for Liogenys punctaticollis

XML Treatment for Liogenys rufocastanea

XML Treatment for Liogenys santaecrucis

XML Treatment for Liogenys sinuaticeps

XML Treatment for Liogenys spiniventris

XML Treatment for Liogenys suturalis

XML Treatment for Liogenys tarsalis

XML Treatment for Liogenys testaceipennis

XML Treatment for Liogenys tibialis

XML Treatment for Liogenys unicolor

XML Treatment for Liogenys cavifrons

XML Treatment for Liogenys femella

XML Treatment for Liogenys piauiensis

XML Treatment for Liogenys rotundicollis

XML Treatment for Liogenys freyi

XML Treatment for Liogenys pseudosanctaecrucis

XML Treatment for Liogenys grossii

XML Treatment for Liogenys pseudospiniventris

XML Treatment for Liogenys sulcoventris

## Supplementary materials

Supplementary material 1 Builder Lucid 3.3 key to New World Diplotaxini genera Data type: species data This dataset is made available under the Open Database License (http://opendatacommons.org/licenses/odbl/1.0/). The Open Database License (ODbL) is a license agreement intended to allow users to freely share, modify, and use this Dataset while maintaining this same freedom for others, provided that the original source and author(s) are credited.Mariana Alejandra Cherman, Kleber Makoto Mise, Miguel Angel Morón, Fernando Z. Vaz-de-Mello, Lúcia Massutti de Almeida
